# Metabotropic Glutamate Receptor–Dependent Synaptic Plasticity in Age‐Related Neurodegenerative Disorders

**DOI:** 10.1155/np/3455379

**Published:** 2026-05-14

**Authors:** Shaik Basha, Aradhika Vijeev, Spandana S. Nadig, Kripa Agarwal, Vibhuti Meharchandani, Aparna Ramakrishna Pai, Krishna Kishore Mahato

**Affiliations:** ^1^ Department of Biophysics, Manipal School of Life Sciences, Manipal Academy of Higher Education, Manipal, India, manipal.edu; ^2^ Manipal School of Life Sciences, Manipal Academy of Higher Education, Manipal, India, manipal.edu; ^3^ Department of Neurology, Kasturba Medical College, Manipal Academy of Higher Education, Manipal, India, manipal.edu

**Keywords:** Alzheimer’s disease, metabotropic glutamate receptors, neurodegeneration, Parkinson’s disease

## Abstract

Synaptic plasticity is a fundamental property of the nervous system that underpins learning, memory, and adaptive behavior across the lifespan. Disruption of plasticity mechanisms is increasingly recognized as a unifying feature of age‐related neurodegenerative and neuropsychiatric disorders. While classical models of long‐term potentiation (LTP) and long‐term depression (LTD) have primarily emphasized ionotropic glutamate receptors (iGluRs), emerging evidence identifies metabotropic glutamate receptors (mGluRs) as central regulators of synaptic stability, metaplasticity, and activity‐dependent translational control. This review synthesizes molecular, synaptic, circuit‐level, and translational evidence to position mGluR–dependent plasticity as a context‐sensitive signaling framework that governs excitatory–inhibitory balance across cortical and subcortical networks. This review examines subtype‐specific contributions of Groups I, II, and III mGluRs to LTP and LTD, highlighting their roles in intracellular calcium dynamics, protein synthesis–dependent plasticity, and neuron–glia interactions. Particular emphasis is placed on receptor localization, intracellular signaling pathways, and region‐specific cortical plasticity, which collectively determine how mGluR signaling shapes functional outcomes across distributed brain circuits. This review further discusses how dysregulation of mGluR–mediated plasticity contributes to synaptic and circuit dysfunction in Alzheimer’s disease (AD), Parkinson’s disease (PD), schizophrenia, autism spectrum disorder (ASD), Fragile X syndrome (FXS), and epilepsy, with attention to disease stage–specific and context‐dependent alterations revealed by electrophysiological, molecular, and receptor imaging studies. Finally, emerging translational strategies, including subtype‐selective biomarkers, allosteric and pathway‐biased modulation, and circuit‐targeted interventions, are evaluated for their potential to restore adaptive plasticity while limiting maladaptive network remodeling. Collectively, this review reframes mGluR–dependent synaptic plasticity as a dynamic and integrative regulator of neuronal circuit function in aging and disease, providing a conceptual and mechanistic foundation for the development of precision neuroplasticity‐based therapeutics.

## 1. Introduction

Neuroplasticity, derived from the Greek plastikós and Latin plasticus, refers to the capacity of the brain to undergo structural and functional reorganization in response to intrinsic and extrinsic stimuli. First conceptualized by William James and later formalized by Konorski, neuroplasticity is now recognized as a fundamental property of the nervous system that underpins learning, memory, and behavioral adaptability across the lifespan [1, 2]. Beyond its role in normal cognition, plasticity is increasingly understood as a dynamic and context‐dependent process that can either support functional recovery or contribute to maladaptive circuit remodeling under pathological conditions, including cerebrovascular injury, epilepsy, and neurodegenerative disorders (NDDs). Despite its central importance, the molecular logic by which plasticity is engaged, constrained, or distorted in aging and disease remains incompletely defined, reflecting the complexity of synaptic signaling networks and their sensitivity to cumulative cellular stressors [1, 2].

Seminal experimental work in the mid‐20th century overturned the long‐standing view of the adult brain as a static organ, demonstrating instead that neuronal circuits retain a remarkable capacity for experience‐dependent reorganization. Studies by Bach‐y‐Rita, Penfield, and Merzenich collectively established that cortical and subcortical networks can be reshaped throughout life in response to sensory input, injury, and environmental demands [3]. However, while such plasticity is adaptive in healthy systems, accumulating evidence indicates that in aging and NDDs, these same mechanisms may become dysregulated, leading to network instability, impaired information processing, and progressive cognitive decline. Unlike developmental plasticity, which occurs within a permissive and growth‐supportive environment, adult plasticity operates within a constrained and often degenerating cellular landscape, raising critical questions regarding the balance between compensatory and pathological remodeling in aging brains [3].

At a mechanistic level, neuroplasticity emerges from the coordinated interplay of synaptic remodeling, intracellular signaling cascades, neuromodulatory influences, and activity‐dependent gene expression. These processes collectively give rise to long‐term potentiation (LTP) and long‐term depression (LTD), widely regarded as cellular substrates of learning and memory [4]. In neurodegenerative conditions such as Alzheimer’s disease (AD), Parkinson’s disease (PD), and related disorders, these plasticity mechanisms are disrupted by distinct pathological drivers, including amyloid‐β accumulation, tau pathology, and α‐synuclein aggregation [5–7]. However, despite extensive experimental work, current models remain limited in their ability to integrate molecular alterations with synaptic, circuit‐level, and behavioral outcomes [8]. Moreover, heavy reliance on animal models, methodological heterogeneity, and the scarcity of robust translational biomarkers continue to hinder the extrapolation of mechanistic insights to human disease [9, 10]. These limitations highlight the need for receptor‐ and pathway‐specific frameworks capable of bridging fundamental synaptic biology with clinical neurodegeneration.

Within this context, metabotropic glutamate receptors (mGluRs) have emerged as critical regulators of synaptic plasticity, acting not as direct mediators of fast synaptic transmission but as integrative signaling hubs that modulate intracellular pathways, synaptic stability, and network excitability [11]. mGluRs are organized into three groups (I–III), and they exert distinct yet overlapping effects on excitatory and inhibitory transmission, synaptic homeostasis, and circuit‐level dynamics. Their spatial distribution across pre‐, post‐, and perisynaptic compartments, along with their coupling to diverse intracellular signaling cascades, enables them to function as context‐sensitive modulators of plasticity rather than static regulators of excitation. Increasing evidence indicates that dysregulation of mGluR signaling contributes directly to the synaptic dysfunction observed in NDDs, influencing processes such as calcium signaling, protein synthesis–dependent plasticity, and neuron–glia communication [12–16].

Despite growing recognition of their importance, existing literature on mGluRs remains fragmented, often focusing on individual receptor subtypes, isolated signaling pathways, or specific disease models without integrating these findings into a unified conceptual framework. In particular, key gaps persist in understanding how mGluR–dependent plasticity is shaped by aging, how receptor subtype–specific signaling translates into circuit‐level dysfunction, and how these processes evolve across disease stages. Furthermore, the interaction between neuronal and astrocytic mGluR signaling, and its contribution to adaptive versus maladaptive plasticity, remains incompletely resolved. Addressing these gaps is essential for developing targeted therapeutic strategies that can selectively restore physiological plasticity while minimizing pathological remodeling. The present review addresses these challenges by synthesizing molecular, synaptic, cellular, and translational evidence to construct an integrative framework of mGluR–dependent synaptic plasticity in age‐related NDDs. By systematically examining receptor localization, intracellular signaling mechanisms, neuron–glia interactions, and circuit‐level plasticity across brain regions, this work advances beyond descriptive summaries to provide a cohesive and hypothesis‐driven perspective. In doing so, it highlights mGluRs as dynamic regulators of synaptic state and identifies key mechanistic nodes that may be leveraged for precision therapeutic intervention in neurodegenerative disease.

## 2. Methodology

### 2.1. Literature Search Strategy

This review employed a comprehensive and integrative literature search strategy to identify experimental, clinical, and translational studies investigating mGluR–dependent synaptic plasticity in physiological and pathological contexts. Systematic searches were conducted across major biomedical databases, including PubMed, Scopus, Web of Science, and Google Scholar. To ensure comprehensive coverage, reference lists of key review articles and seminal studies were manually screened, enabling the inclusion of both foundational and recent advances relevant to LTP, LTD, and neurodegenerative and neuropsychiatric disorders.

### 2.2. Search Terms

Search queries incorporated combinations of controlled vocabulary and free‐text terms, including synaptic plasticity, LTP, LTD, mGluRs, mGluR1, mGluR subtype 5 (mGluR5), Group II mGluRs, cortical plasticity, metaplasticity, protein synthesis–dependent plasticity, NDDs, AD, PD, schizophrenia, autism spectrum disorder (ASD), Fragile X syndrome (FXS), epilepsy, positron emission tomography (PET) imaging, and translational biomarkers.

### 2.3. Time Frame

The primary literature focused spanned publications from 1990 to 2025, capturing both foundational discoveries in synaptic plasticity and recent advances in molecular signaling, neuroimaging, and translational neuroscience. Earlier landmark studies were included where necessary to provide historical and conceptual context.

### 2.4. Language Criteria

Only peer‐reviewed articles published in English were included to ensure consistency in interpretation and scientific rigor.

### 2.5. Inclusion Criteria

Studies were included based on the following criteria:

• Investigation of mGluR–dependent mechanisms underlying LTP, LTD, or metaplasticity.
• Examination of synaptic plasticity within cortical or subcortical circuits.
• Provision of mechanistic, electrophysiological, molecular, imaging, or behavioral evidence.
• Relevance to neurodegenerative or neuropsychiatric disorders.
• Inclusion of translational perspectives, such as biomarkers, imaging approaches, or therapeutic modulation.

### 2.6. Exclusion Criteria

Studies were excluded if they:

• Focused exclusively on ionotropic glutamate receptors (iGluRs) without involvement of mGluRs.
• Lacked mechanistic or functional relevance to synaptic plasticity.
• Were purely descriptive without experimental or translational insight.
• Consisted of abstracts, conference proceedings, or non‐peer‐reviewed sources.

### 2.7. Study Selection and Validation

All selected studies were critically evaluated for relevance, methodological rigor, and consistency with original findings. Citation accuracy was verified through direct consultation of primary source articles rather than reliance on secondary references.

### 2.8. Data Organization and Synthesis

Included studies were thematically categorized into the following domains:

i. Molecular and intracellular signaling mechanisms of mGluR–dependent LTP (mGluR–LTP) and LTD.
ii. Region‐specific cortical and subcortical plasticity.
iii. Translational and imaging‐based evidence.
iv. Disease‐specific dysregulation across neurodegenerative and neuropsychiatric conditions.

Given the heterogeneity of experimental designs, species, and outcome measures, findings were synthesized using a mechanistic–integrative approach rather than formal meta‐analysis.

### 2.9. Identification of Knowledge Gaps

The literature analysis identified several key gaps, including limited in vivo human evidence for mGluR–dependent plasticity, insufficient understanding of developmental and disease stage–specific signaling dynamics, incomplete integration of neuron–glia interactions, and challenges in translating preclinical findings into effective clinical interventions.

### 2.10. Novelty and Concept Framework

This review advances beyond descriptive summarization by integrating receptor subtype–specific signaling, translational control mechanisms, and region‐specific cortical plasticity into a unified conceptual framework. In this context, mGluRs are positioned as dynamic and conditional regulators of synaptic state, rather than static modulators of excitatory transmission.

### 2.11. Theoretical and Translational Significance

From a theoretical perspective, this work refines existing models of synaptic plasticity by incorporating metaplastic and translational dimensions of mGluR signaling. From a translational standpoint, it informs the development of subtype‐selective biomarkers and precision therapeutic strategies aimed at restoring adaptive plasticity while minimizing maladaptive network remodeling.

### 2.12. Limitations

This review is subject to several limitations, including reliance on heterogeneous experimental paradigms and disease models, which preclude quantitative meta‐analysis. Additionally, species differences and limited longitudinal human data constrain direct clinical extrapolation of mechanistic findings.

## 3. Structural Organization and Subcellular Distribution of mGluRs

mGluRs exhibit a precisely organized spatial distribution within neuronal membranes that critically shapes their functional roles in synaptic signaling and plasticity. Unlike iGluRs that are typically concentrated within the postsynaptic density (PSD), mGluRs are predominantly localized in perisynaptic and extrasynaptic membrane domains, where they are strategically positioned to detect glutamate spillover during periods of intense synaptic activity. This spatial arrangement enables mGluRs to function primarily as modulatory regulators of synaptic transmission rather than direct mediators of fast excitatory signaling, thereby linking their localization to slower and integrative forms of synaptic plasticity such as mGluR–dependent LTP and LTD. Increasing evidence from high‐resolution imaging, immunohistochemistry, and electron microscopy indicates that the distribution of mGluR subtypes is tightly regulated across neuronal compartments, including dendritic spines, axonal terminals, and glial‐associated membrane regions, and that subtype‐specific localization patterns further determine how individual receptors engage intracellular signaling pathways. The studies summarized in Table 1 collectively demonstrate that mGluRs are segregated from ionotropic receptors at the nanoscale level and occupy distinct synaptic, perisynaptic, and extrasynaptic domains in a cell type‐, circuit‐, and developmentally specific manner.

**Table 1:** Experimental evidence for the structural and subcellular organization of mGluRs.

Study	Model/brain region	Methods	mGluR subtypes	Subcellular localization	Key findings
[17]	Adult rhesus monkey; subthalamic nucleus (STN)	Double‐immunofluorescence, immunoperoxidase (LM + EM), preembedding immunogold EM, postembedding GABA immunogold	mGluR1a, mGluR5 (Group I)	Mainly postsynaptic dendrites; ~60%–70% intercellular, ~30%–40% membrane‐bound; >90% extrasynaptic (perisynaptic), minimal in synapse; mGluR1a also in glia (more than mGluR5)	mGluR1a and mGluR5 have similar subcellular distribution; mainly extrasynaptic/perisynaptic near both GABAergic and glutamatergic synapses, so functional differences are not due to location alone
[18]	Rat; hippocampus (CA1, CA3, dentate gyrus)	Immunoperoxidase LM, pre‐embedding immunogold EM, quantitative ultrastructural analysis	mGluR1a, mGluR5 (Group I)	Predominantly postsynaptic dendritic spines, shafts and somatodendritic membranes; enriched perisynaptically, sprse in synaptic core, with lower extrasynaptic presence; also in interneuron somatodendritic regions	Group I mGluRs are enriched perisynaptically in an annular pattern around synapses, with density decreasing outward; separated from the synaptic core, supporting a modulatory role
[19]	Rat: developing neocortex and hippocampus (prenatal → adult stages)	Immunohistochemistry (LM), pre‐embedding immunogold EM, developmental expression analysis	mGluR1α, mGluR5 (Group I)	Localized in neuronal stomata, dendrites and neurophil (neocortex and hippocampus); enriched perisynaptically and extrasynaptically, minimal in synaptic core; developmental shift: mGluR1α (neuropil → somata), mGluR5 (somata → neuropil)	Group I mGluRs show developmentally regulated, subtype‐specific expression; mGluR1α in GABAergic neurons, mGluR5 in both pyramidal and interneurons; redistributed supports roles in differentiation
[20]	Rat; hippocampus (CA1, CA3, dentate gyrus; perforant path, mossy fiber, Schaffer collateral pathways)	Immunohistochemistry (LM), immunogold and immunoperoxidase EM, pathway‐specific lesion analysis	mGluR1, mGluR5 (Group I); mGluR2, 4a, 7a, 7b, 8 (Groups II and III)	Group I (mGluR1/5): postsynaptic dendrites and omatic elements within the neuropil. Groups II/III: mainly presynaptic—Group III at active zones, Group II (mGluR2) in preterminal axons (extrasynaptic)	Clear segregation: Group I are postsynaptic, Groups II/III presynaptic; Group III at active zones, mGluR2 in preterminal axons; pathway‐ and target‐specific distribution enables precise control of neurotransmitter release
[21]	Cultured hippocampal neurons (rat/mouse); dendritic spines (excitatory synapses)	Live‐cell imaging, superresolution microscopy (SMLM), single‐molecule tracking (uPAINT), molecular manipulation (FKBP–FRB system), Ca^2+^ imaging (GCaMP6f)	mGluR5 (Group I)	Organized in dynamic nanodomains within the ~200 nm perisynaptic zone; excluded from the synaptic core but near its edge; show transient mobility; positioning regulated by C‐terminal interactions	mGluR5 is transiently confined to dynamic perisynaptic nanodomains; C‐terminal domain controls synaptic exclusion and organization; synaptic recruitment enhances Ca^2+^ signaling, showing position‐dependent modulation

At the level of individual excitatory synapses, Scheefhals and MacGillavry [22] examined the functional organization of glutamate receptors at the postsynaptic membrane and showed that ionotropic and metabotropic receptors occupy distinct subsynaptic compartments. Electron microscopy and immunogold labeling demonstrated that AMPA and NMDA receptors (NMDARs) are predominantly concentrated within the PSD directly opposite the presynaptic vesicle release site, whereas Group I mGluRs (mGluR1 and mGluR5) are largely excluded from the PSD and instead accumulate in an annular perisynaptic domain approximately 100–200 nm surrounding the PSD. Computational and experimental work indicated that glutamate released from synaptic vesicles generates a steep and transient concentration gradient in the synaptic cleft, such that receptors located closest to the release site, particularly AMPA receptors within PSD nanodomains aligned with presynaptic active zones, have the highest activation probability during single vesicle release events, whereas mGluRs in the perisynaptic zone are less efficiently engaged by isolated synaptic events. Because glutamate concentrations decline rapidly away from the release site and are further limited by uptake through glutamate transporters, Group I mGluRs are proposed to respond preferentially during high‐frequency activity or glutamate spillover, allowing them to act as integrators of sustained synaptic activity rather than mediators of fast transmission. The authors also described molecular mechanisms that may regulate receptor positioning within the postsynaptic membrane, including interactions between AMPA receptors and scaffolding proteins such as PSD‐95, and binding of Group I mGluRs to Homer proteins via their C‐terminal domains (CTDs), as well as possible contributions from steric constraints within the densely packed PSD and cytoskeletal barriers at the perisynaptic border. Together, these observations highlight that nanoscale segregation of iGluRs and mGluRs within synaptic and perisynaptic compartments is a key structural determinant of receptor activation probability and provides a framework for understanding how receptor localization contributes to synaptic signaling and plasticity [22].

Building on this conceptual framework, Scheefhals et al. [21] used live‐cell imaging, super‐resolution microscopy, and single‐molecule tracking to define the nanoscale organization and mobility of mGluR5 in hippocampal neurons. Gated stimulated emission depletion (gSTED) microscopy and immunolabeling relative to PSD markers such as PSD‐95 and Homer1c showed that surface mGluR5 is enriched in dendritic spines but largely excluded from the PSD core, instead localizing to the perisynaptic zone surrounding the PSD at distances of approximately 200 nm from the synaptic center. Super‐resolution single‐molecule localization microscopy revealed that mGluR5 is further organized into discrete perisynaptic nanodomains positioned approximately 200–300 nm from the PSD border, rather than being uniformly distributed in the spine membrane. Single‐molecule tracking indicated that receptor mobility is heterogeneous across synaptic subregions, with many trajectories confined within the perisynaptic zone and only a minority entering the PSD, and receptors in perisynaptic regions exhibiting reduced diffusion and transient confinement, consistent with local retention mechanisms. Mechanistic analyses implicated the intracellular CTD of mGluR5 in maintaining this perisynaptic confinement, as CTD truncation increased receptor mobility and produced a more homogeneous membrane distribution, while also permitting greater receptor entry into the PSD. An inducible recruitment system used to artificially relocate mGluR5 into synapses increased synaptic calcium responses, suggesting that its normal perisynaptic positioning shapes how synaptic signals are transduced. These findings support the view that mGluR5 resides in dynamic perisynaptic nanodomains rather than within the synaptic core, and that its nanoscale distribution and mobility constrain receptor activation patterns and thereby influence synaptic signaling [21].

While these studies define the fine‐scale organization of Group I receptors at individual synapses, anatomical work by Shigemoto et al. [20] extended the analysis to the level of hippocampal circuits and afferent pathways. Using subtype‐specific antibodies against mGluR1–mGluR8 in combination with immunohistochemistry, lesion experiments, and electron microscopy, the authors showed a clear compartmental segregation of receptor groups, with Group I mGluRs (mGluR1 and mGluR5) primarily localized to postsynaptic elements in dendritic fields of principal neurons and Groups II/III receptors (mGluR2, mGluR4a, mGluR7a, mGluR7b, and mGluR8) predominantly associated with presynaptic structures. Distinct laminar patterns of immunoreactivity corresponded closely to the termination zones of major hippocampal afferents: for example, strong labeling for mGluR2 and mGluR7a was detected in medial perforant path terminal zones, whereas mGluR8 was prominent in lateral perforant path projections in the dentate gyrus and CA3, and mossy fibers and Schaffer collaterals (SCs) showed subtype‐specific combinations of Groups II and III receptors. Lesion experiments targeting the entorhinal cortex (EC) or hippocampal neuronal populations confirmed that many of these receptor signals originated from defined afferent projections. Ultrastructural analysis further showed that Group II receptors, such as mGluR2, were typically localized to preterminal segments of axons rather than directly at synaptic release sites, whereas Group III receptors were frequently concentrated within presynaptic active zones of axon terminals, indicating that different presynaptic mGluR subtypes occupy distinct positions along axons and terminals that are likely to support differential control of neurotransmitter release. In addition, target cell‐specific differences were reported, particularly for mGluR7 variants, where receptor localization depended on the identity of the postsynaptic neuron contacted by the presynaptic axon. Collectively, these observations indicate that mGluR subtypes show highly specific and pathway‐dependent distributions within hippocampal circuits, with postsynaptic Group I receptors largely associated with dendritic compartments and presynaptic Group II/III receptors positioned along axonal and terminal domains that modulate synaptic transmission [20].

Developmental studies further demonstrate that the structural organization and subcellular localization of Group I mGluRs are dynamically regulated during corticogenesis. López‐Bendito [19] examined the expression of mGluR1α and mGluR5 from embryonic stages through postnatal development using light microscopy, confocal double‐labeling, and electron microscopy, and found that mGluR5 expression appears first at embryonic day E18 with moderate labeling in the hippocampus and strong diffuse immunoreactivity in neocortical marginal zone, subplate, and cortical plate, including Cajal–Retzius cells and pioneer neurons of the marginal zone. At the same embryonic stage, mGluR1α expression was weak and diffuse, with no clearly labeled neuronal somata in the developing cortex or hippocampus, but during the first postnatal week, mGluR1α became strongly expressed in Layer I neurons resembling Cajal–Retzius cells, and double‐labeling experiments showed that approximately 98% of reelin‐positive Cajal–Retzius cells coexpressed mGluR1α. As development progressed and Cajal–Retzius cells disappeared, mGluR1α expression shifted to cortical interneurons distributed across Layers II–VI, whereas mGluR5 became predominantly associated with pyramidal neurons and neuropil regions, with labeling concentrated in dendritic compartments rather than somata. In the hippocampus, mGluR5 immunoreactivity was particularly strong in strata oriens and radiatum of CA1 and CA3, whereas the stratum pyramidale showed little or no staining. Electron microscopy indicated that both mGluR1α and mGluR5 were localized exclusively to postsynaptic elements, including dendritic shafts and spines, with immunogold particles positioned at perisynaptic and extrasynaptic membrane sites adjacent to, but not within, the PSD, and no presynaptic labeling was detected for either receptor. These findings show that Group I mGluRs undergo subtype‐specific and cell type‐specific changes in expression during development and are preferentially positioned in perisynaptic and extrasynaptic membrane domains of dendrites and spines, in keeping with a modulatory role in synaptic signaling during cortical and hippocampal maturation [19].

Consistent with this, Luján et al. [18] provided a detailed ultrastructural analysis of Group I mGluR localization in the adult rat hippocampus using immunocytochemistry with pre‐embedding and postembedding electron microscopy. Light microscopic data revealed distinct laminar patterns of expression, with mGluR5 immunoreactivity in principal neurons across hippocampal regions, particularly in strata oriens and radiatum of CA1, and weaker labeling in CA3 and the dentate gyrus molecular layer, whereas mGluR1 showed strong labeling in interneurons and pyramidal cells of CA3 and in dentate granule cells, with little or no labeling in CA1 pyramidal neurons. Ultrastructural analysis demonstrated that both mGluR1 and mGluR5 were predominantly localized to postsynaptic dendritic shafts and spines receiving excitatory synapses, with immunogold particles consistently located outside the core PSD and concentrated in perisynaptic and extrasynaptic regions of the plasma membrane, often forming an annular distribution around Type I synapses. Quantitative analysis of immunogold particles on dendritic spines in CA1 stratum radiatum showed that the highest density of mGluR5 labeling occurred within approximately 60 nm of the edge of the synaptic specialization, with receptor density decreasing at greater distances along the spine membrane, consistent with a perisynaptic enrichment zone surrounding glutamate release sites. Similar perisynaptic distributions were observed at perforated synapses, where labeling surrounded the discontinuous edges of the PSD, and receptors were also detected along somatic and dendritic membranes outside synaptic junctions, indicating the existence of both perisynaptic and extrasynaptic receptor pools. These observations reinforce the conclusion that Group I mGluRs are segregated from iGluRs within hippocampal synapses, with iGluRs occupying the PSD and mGluRs positioned primarily in surrounding perisynaptic domains, and suggest that this arrangement supports preferential activation of metabotropic receptors by glutamate spillover during intense or repetitive synaptic activity [18].

Work in other basal ganglia structures indicates that similar principles of perisynaptic and extrasynaptic positioning of Group I mGluRs extend beyond the hippocampus. Kuwajima et al. [17] analyzed the subcellular and subsynaptic localization of mGluR1a and mGluR5 in monkey subthalamic nucleus (STN) neurons using double‐immunofluorescence confocal microscopy, immunoperoxidase labeling, and pre‐embedding immunogold electron microscopy, and reported that both receptor subtypes are strongly coexpressed in neuronal somata and neuropil elements, with most neurons showing immunoreactivity for both receptors. Astrocytic cell bodies showed strong mGluR1a immunoreactivity but lacked detectable mGluR5 labeling, indicating cell type‐specific differences in receptor expression. At the ultrastructural level, both receptors were localized predominantly in postsynaptic dendritic structures, including dendritic shafts and occasional spine‐like protrusions, whereas presynaptic axon terminals rarely displayed labeling, and dendrites and synaptic elements were often ensheathed by glial processes that also contained mGluR1a. High‐resolution immunogold analysis showed that approximately 60%–70% of immunogold particles for mGluR1a and mGluR5 were located intracellularly within dendritic compartments, with 30%–40% associated with the plasma membrane; among the membrane‐associated particles, more than 90% were at extrasynaptic sites and only a small fraction at synaptic or perisynaptic positions relative to PSDs. When receptors were associated with synapses, labeling was most often detected at the edges of both symmetric (GABAergic) and asymmetric (glutamatergic) synapses, and quantitative analysis indicated that perisynaptic receptor labeling occurred more frequently than expected from a random membrane distribution, suggesting selective enrichment near synaptic borders. These findings show that mGluR1a and mGluR5 in STN neurons exhibit largely similar subcellular and subsynaptic localization patterns characterized by predominant dendritic expression and strong enrichment in perisynaptic and extrasynaptic plasma membrane domains, a distribution compatible with activation by glutamate spillover or nonsynaptic release rather than by direct synaptic transmission [17].

Across these studies, converging evidence from different brain regions, developmental stages, and methodological approaches indicates that mGluRs are organized in a highly structured manner at the level of synapses, dendrites, axons, and glial interfaces, with Group I receptors primarily occupying postsynaptic perisynaptic and extrasynaptic domains and Group II/III receptors distributed along presynaptic elements. This compartmentalized distribution provides a structural substrate by which specific mGluR subtypes are differentially engaged during distinct activity patterns and glutamate signaling regimes, thereby setting the stage for their distinct contributions to mGluR–dependent forms of LTP, LTD, and other modes of synaptic plasticity that are discussed in subsequent sections. At the same time, most available data derive from fixed‐tissue preparations and selected brain regions, and often lack direct correlation with in vivo activity states, leaving open questions about how dynamic receptor trafficking, neuromodulatory context, and disease‐related remodeling alter these spatial arrangements overtime. Future work combining high‐resolution structural approaches with live‐cell and in vivo imaging, as well as subtype‐selective genetic and pharmacological tools, will be important to define how these localization principles are maintained or disrupted across circuits, developmental stages, and pathological conditions, and how such changes translate into specific forms of synaptic plasticity.

## 4. Intracellular Signaling Mechanisms of mGluRs

Activation of mGluRs initiates diverse intracellular signaling cascades that regulate neuronal excitability, synaptic transmission, and long‐term synaptic plasticity. As Class C G‐protein–coupled receptors, mGluRs transduce glutamatergic signals into intracellular biochemical responses through subtype‐specific coupling to heterotrimeric G proteins. Group I mGluRs (mGluR1 and mGluR5) primarily couple to Gq/11 proteins, activating phospholipase Cβ (PLCβ) and generating inositol‐1,4,5‐trisphosphate (IP_3_) and diacylglycerol (DAG), whereas Groups II and III receptors couple mainly to Gi/o proteins that inhibit adenylate cyclase and reduce cyclic AMP signaling. Beyond these canonical pathways summarized in Table 2, mGluR activation engages kinase cascades, receptor phosphorylation, modulation of ion channel activity, and regulation of receptor trafficking. These signaling processes collectively shape synaptic responsiveness and neuronal excitability by controlling membrane conductances, intracellular calcium dynamics, and protein phosphorylation networks, thereby providing a biochemical substrate for mGluR–dependent forms of LTP, LTD, and metaplasticity.

**Table 2:** Intracellular signaling pathways engaged by mGluR activation.

Study	mGluR subtype	Experimental model	Intracellular signaling pathway	Functional outcome
[23]	Groups I–III mGluRs	Excitatory synapses (pre‐ and postsynaptic neurons)	Group I: Gq/11‐coupled, postsynaptic (perisynaptic) Group II: Gi/o‐coupled, axonal/perisynaptic Group III: Gi/o‐coupled, presynaptic (active zone); positioning determines signaling	Group I: modulate postsynaptic excitability and plasticity (LTP/LTD) Groups II/III: inhibit neurotransmitter release; modulate synaptic transmission/plasticity
[24]	Group I (mGluR5)	Striatal neurons (rat; in vitro + ex vivo slices)	Gq → PLCβ → IP_3_/Ca^2+^ signaling; ERK1 directly binds mGluR5 C‐terminal and phosphorylates Ser1126 (distal CT)	ERK–dependent phosphorylation enhances mGluR5 efficacy in IP_3_ production, modulating receptor signaling
[25]	Group I (mGluR5)	Spinal dorsal horn neurons + HEK293T cells (in vitro and ex vivo)	mGluR5 → ERK activation (independent of canonical Gq/PKC); ERK phosphorylates GlyR α1ins at Ser380 → promotes ubiquitination (Lys379) → receptor endocytosis	Suppression of glycinergic inhibitory transmission via α1ins downregulation contributes to pain sensitization
[26]	mGlu3 (Group II; interacts with mGlu5)	Hippocampal CA1 pyramidal neurons; mouse trace fear conditioning + ex vivo slices	mGlu3 activation → coordinated mGlu5–dependent signaling → endocannabinoid (CB1)–mediated suppression of GABAergic inhibition (disinhibition) → metaplastic shift toward LTP	Enhances hippocampal LTP and rescues schizophrenia‐like cognitive deficits (improved associative learning)
[27]	mGluR6 (Group III)	Rat embryonic cortical neural stem cells (NSCs, in vitro)	ERK1/2 → Cyclin D1/CDK2 (cell cycle promotion); ↓ p38 MAPK (antiapoptotic)	Promotes NSC proliferation (G1→S transition) and inhibits apoptosis
[28]	Group I (mGluR1/5)	Entorhinal cortex Layer III pyramidal neurons (rat brain slices)	Gq/11 → PLCβ → PIP_2_ depletion + DAG → TRPC5 activation; inhibition of GIRK channels (PKC‐ and Ca^2+^‐independent)	Direct excitation of pyramidal neurons and enhanced neuronal excitability (supports persistent firing/memory processes)

At a mechanistic level, the intracellular signaling capacity of mGluRs is closely linked to their structural organization and posttranslational regulation. Suh et al. [29] systematically examined the molecular mechanisms regulating trafficking and intracellular signaling of mGluRs, emphasizing the role of posttranslational modifications and receptor–protein interactions in controlling receptor localization and signaling efficiency. mGluRs possess a large extracellular ligand‐binding domain and an intracellular CTD that serves as a platform for phosphorylation and protein–protein interactions regulating receptor trafficking and signaling. Group I receptors couple primarily to Gαq/11 proteins, activating phospholipase C (PLC) and generating IP_3_ and DAG, which promote intracellular Ca^2+^ release and PKC activation, whereas Groups II and III receptors couple predominantly to Gi/o proteins, suppressing adenylate cyclase and protein kinase A (PKA) signaling. Receptor trafficking and signaling are further regulated by phosphorylation mediated by PKC, PKA, Ca^2+^/calmodulin–dependent kinase II (CaMKII), mitogen‐activated protein kinases (MAPKs/extracellular signal‐regulated kinase [ERK]), and G‐protein‐coupled receptor kinases (GRKs), which influence desensitization, internalization, and signaling competence. For example, PKC–dependent phosphorylation of Group I receptors regulates agonist‐induced desensitization and internalization, while CaMKII binding to the mGlu1 CTD promotes phosphorylation‐dependent endocytosis and attenuates IP_3_–mediated signaling. Scaffold proteins and interacting partners, including Homer, calmodulin, Siah‐1A ubiquitin ligase, and tamalin, assemble receptor‐associated signaling complexes that control surface expression and coupling to intracellular pathways. Collectively, these mechanisms indicate that mGluR signaling is dynamically regulated by phosphorylation, scaffold‐mediated interactions, and trafficking processes that determine receptor availability and signaling output at synapses [29].

The importance of this framework for shaping intracellular signaling is further supported by work on how receptor positioning at excitatory synapses constrains coupling to local effectors. Bodzęta et al. [23] reviewed the trafficking, positioning, and signaling functions of mGluRs at excitatory synapses, highlighting how receptor localization influences engagement with intracellular signaling pathways. Group I mGluRs (mGluR1 and mGluR5) couple predominantly to Gq/11 proteins, whereas Group II (mGluR2 and mGluR3) and Group III receptors (mGluR4, 6, 7, and 8) couple mainly to Gi/o proteins, consistent with their canonical roles in PLC activation versus adenylate cyclase inhibition. Electron microscopy and super‐resolution imaging indicate that Group I mGluRs are enriched in perisynaptic regions surrounding the PSD, positioning them to respond to strong synaptic activity and modulate postsynaptic excitability and plasticity. Group II receptors are distributed along axons and perisynaptic regions but are largely excluded from active zones, whereas Group III receptors are enriched at presynaptic active zones where they regulate neurotransmitter release. Receptor positioning influences coupling to local signaling effectors and synaptic machinery, including voltage‐gated calcium channels (VGCCs), potassium channels, and vesicle release components. In addition, intracellular mechanisms controlling receptor trafficking, such as secretory pathway processing, dimerization, glycosylation, and interactions with Homer, Tamalin, and Norbin, regulate surface expression and signaling competence. These observations indicate that the intracellular signaling output of mGluRs is tightly coupled to their dynamic trafficking and spatial organization at synapses [23].

Several experimental studies further delineate how specific kinases and ion channels interface with individual mGluR subtypes to regulate synaptic signaling and excitability. Jin et al. [24] investigated the role of ERK (ERK1), a MAPK family member, in regulating intracellular signaling and functional activity of mGlu5. In vitro binding assays showed that ERK1 selectively binds to the CT1 region of the mGlu5 CTD, and pull‐down experiments from rat striatal tissue confirmed association of endogenous ERK1 with mGlu5. Active ERK1 phosphorylated the C‐terminal tail of mGlu5 at a serine residue (S1126) within an ERK consensus motif, and co‐immunoprecipitation revealed constitutive serine phosphorylation of mGlu5 under basal conditions. Pharmacological inhibition of ERK signaling with the MEK inhibitor U0126 significantly reduced mGlu5–dependent IP_3_ production in striatal slices following stimulation with the Group I agonist DHPG. These findings indicate that ERK1 interacts with the mGlu5 CTD, phosphorylates specific serine residues, and modulates the efficiency of downstream phosphoinositide signaling in striatal neurons, providing a mechanism by which activity‐dependent kinase activation can regulate mGluR–driven second‐messenger production [24]. Furthermore, Zhang et al. [25] examined another ERK–dependent pathway through which mGluR5 regulates inhibitory glycinergic neurotransmission in the spinal dorsal horn. In spinal cord slices, brief application of DHPG reduced glycine receptor–mediated inhibitory postsynaptic currents without affecting GABA Type A receptor–mediated IPSCs, indicating selective modulation of glycinergic transmission. This effect was mediated specifically by mGluR5, as it was blocked by the antagonist MPEP, whereas inhibition of mGluR1 had no effect. Suppression of glycinergic currents did not depend on canonical G‐protein or PKC signaling, as intracellular GDP‐β‐S and PKC inhibitors did not prevent DHPG–induced depression, but required ERK activation, since MEK inhibitors abolished the reduction in glycinergic responses. The signaling cascade selectively targeted the α1ins splice variant of the glycine receptor α1 subunit, which contains an eight amino acid insertion in its intracellular loop. In heterologous systems, mGluR5 activation reduced currents mediated by α1ins but not α1 or α3L, indicating subtype‐specific regulation. Activated ERK interacted with the intracellular loop of α1ins via a D‐docking motif generated by the insertion and phosphorylated α1ins at Ser380; mutation of this residue prevented mGluR5–mediated inhibition of α1ins currents. Ser380 phosphorylation promoted ubiquitination of α1ins at Lys379, facilitating interaction with endocytic adaptor proteins and receptor internalization. Together, these findings indicate that mGluR5 can engage an ERK–dependent pathway that regulates inhibitory synaptic transmission by promoting phosphorylation‐dependent ubiquitination and endocytosis of α1ins‐containing glycine receptors in dorsal horn neurons, thereby influencing excitatory inhibitory balance [25].

Group II mGluRs also recruit distinct intracellular signaling mechanisms that interact with other glutamatergic and neuromodulatory systems to shape plasticity. Dogra et al. [26] investigated how activation of mGlu3 regulates hippocampal synaptic plasticity and cognition. Systemic administration of the mGlu2/3 agonist LY379268 enhanced associative learning in trace fear conditioning, and this effect was abolished by the selective mGlu3 negative allosteric modulator (NAM) VU0650786, indicating a specific contribution of mGlu3. In hippocampal slices, mGlu3 activation enhanced theta‐burst stimulation–induced LTP at CA3–CA1 synapses, whereas pharmacological inhibition of mGlu3 prevented this enhancement and shifted synaptic responses away from LTP and toward LTD. This metaplastic effect required functional coupling between mGlu3 and mGlu5, as the mGlu5 antagonist MTEP blocked mGlu3–mediated potentiation of LTP. Additional experiments showed that mGlu3 activation suppressed inhibitory postsynaptic currents onto CA1 pyramidal neurons via an endocannabinoid (eCB)‐mediated disinhibition mechanism involving CB1 receptors. Conditional deletion of mGlu3 from hippocampal pyramidal neurons abolished both LTP enhancement and the associated improvement in associative learning, indicating a requirement for postsynaptic neuronal mGlu3. These findings show that mGlu3 engages intracellular signaling pathways that interact with mGlu5–dependent and eCB signaling to regulate hippocampal synaptic plasticity and circuit function, thereby influencing cognitive processes [26].

Beyond mature synapses, mGluR–dependent intracellular signaling also contributes to developmental and stem cell–related processes. Zhang et al. [27] investigated how mGluR 6 (mGluR6) regulates the biological functions of rat embryonic neural stem cells (NSCs). Overexpression of mGluR6 in primary cortical NSCs increased cell viability, neurosphere diameter, and the proportion of cells in S phase, whereas siRNA–mediated knockdown reduced proliferation and neurosphere growth and increased G0/G1 phase occupancy, indicating a positive role in NSC proliferation and cell‐cycle progression. Apoptosis assays showed that mGluR6 overexpression decreased early and late apoptotic cell populations, while receptor silencing increased apoptosis. Western blot analyses revealed that mGluR6 overexpression increased ERK1/2 phosphorylation and upregulated cyclin D1 and CDK2, whereas knockdown reduced these components. In contrast, phosphorylated p38 MAPK decreased with mGluR6 overexpression and increased after knockdown, suggesting differential regulation of MAPK signaling branches. These findings indicate that mGluR6 modulates intracellular signaling in NSCs by activating the ERK1/2–cyclin D1/CDK2 pathway to promote cell cycle progression and by suppressing p38 MAPK signaling associated with apoptosis, thereby influencing the proliferation and survival of embryonic NSCs [27].

In cortical circuits, coupling of Group I mGluRs to specific ionic effectors provides another mechanism linking intracellular signaling to excitability changes relevant for plasticity induction. Lei et al. [28] examined how activation of Group I mGluRs (mGluR1 and mGluR5) modulates the excitability of Layer III pyramidal neurons in the medial EC. Application of the Group I agonist DHPG produced robust increases in action potential firing, membrane depolarization, inward holding currents, increased input resistance, and prolonged membrane time constants, indicating modifications of intrinsic membrane conductances. Antagonist experiments showed that both mGluR1 and mGluR5 contribute to these responses, with combined blockade abolishing the inward current. Current–voltage analysis revealed two complementary ionic mechanisms: activation of a nonselective cation conductance and suppression of inwardly rectifying potassium (Kir) channels. Genetic and pharmacological data implicated TRPC5 channels in the cationic current, as DHPG‐evoked currents were reduced in TRPC5 knockout (KO) mice but unchanged in TRPV1 or TRPC4 KOs, while inhibition of GIRK (Kir3) channels contributed to the depolarizing response. These effects required G‐protein activation and PLCβ signaling, but not intracellular Ca^2+^ release or PKC activity. Additional experiments indicated that PLCβ–mediated depletion of phosphatidylinositol‐4,5‐bisphosphate (PIP2) contributes to GIRK channel suppression, whereas DAG signaling participates in TRPC5 activation. Together, these findings demonstrate that Group I mGluRs increase neuronal excitability in entorhinal neurons through G‐protein–dependent PLCβ pathways that concurrently activate TRPC5 channels and inhibit GIRK channels, thereby modulating membrane conductance and firing properties [28].

While current evidence delineates core intracellular pathways downstream of mGluR activation, several challenges constrain a complete understanding of how these signaling mechanisms govern synaptic plasticity across circuits and disease states. A major limitation is that most mechanistic insights derive from reductionist preparations, heterologous systems, acute slices, or fixed tissue, which only partially capture the dynamic, activity‐dependent coupling of mGluRs to G‐proteins, PLC and adenylate cyclase, MAPK cascades, and ion channel or trafficking machinery in vivo. In addition, subtype‐ and splice variant–specific signaling, for example, distinct CTD phosphorylation codes, differential engagement of ERK1/2 versus p38 MAPK, or variant‐specific interactions with scaffolds, remains incompletely mapped in native synapses, particularly in defined neuronal versus glial compartments and across development and aging. The context‐dependence of mGluR signaling represents a further challenge: the same receptor can bias networks toward LTP, LTD, or metaplastic shifts depending on neuromodulatory tone, prior activity history, and receptor cross talk, yet systematic frameworks linking specific intracellular states to defined plasticity outcomes are still lacking. Future work will likely require combining subtype‐selective genetic and pharmacological tools with high‐resolution imaging of signaling events, quantitative phosphoproteomics, and in vivo recordings to track how G‐protein coupling, kinase activation, and ion channel regulation evolve during plasticity induction and consolidation in identified circuits. A key opportunity is to extend these approaches to astrocytes and other glial cells, where mGluR–dependent pathways may regulate gliotransmitter release, calcium signaling, and metabolic support, thereby shaping neuron–glia plasticity in parallel with neuronal signaling. In translational terms, dissecting pathway‐ and cell type––pecific mGluR signaling may enable the development of biased ligands and allosteric modulators that selectively engage beneficial intracellular routes, while minimizing adverse effects arising from global receptor blockade or activation. Addressing these challenges will be essential for linking the intracellular mechanisms summarized here to region‐specific plasticity, astrocytic mGluR functions, and mGluR dysregulation in NDDs, and for ultimately translating intracellular pathway knowledge into precise therapeutic strategies targeting maladaptive circuit remodeling.

## 5. Astrocytic mGluRs and Neuron–Glia Plasticity

Astrocytic mGluRs constitute a key interface between neuronal activity, gliotransmission, and circuit remodeling, with distinct mGluR subtypes engaging specific intracellular pathways that differentially shape synaptic and network‐level plasticity across development, health, and disease. In line with this, recent work has begun to dissect how Group I (mGluR1/5) versus Groups II/III (mGluR2/3 and mGluR4/6/7/8) receptors on astrocytes regulate Ca^2+^ dynamics, glutamate uptake, and channel‐mediated glutamate release to tune thresholds for LTP/LTD, interneuron recruitment, and large‐scale reorganization after injury or neurotoxic stress. These converging findings are summarized in Table 3, which provides an overview of astrocytic mGluR subtypes, underlying intracellular mechanisms, and their contributions to synaptic and network‐level plasticity, to support a model in which astrocytic mGluR signaling operates as a bidirectional control system for synaptic strength and excitability, with developmental programs being re‐engaged in adult pathology to drive maladaptive neuron–glia plasticity.

**Table 3:** Overview of astrocytic mGluR subtypes, underlying intracellular mechanisms, and their contributions to synaptic and network‐level plasticity.

Study	Model/system	mGluR subtypes	Astrocytic mechanism	Functional outcome
[30]	Mixed cortical neuron–astrocyte coculture	mGluR5	Astrocytic mGluR5 participates in glutamate sensing and intracellular Ca^2+^ signaling	mGluR5 inhibition decreases neuronal activity and attenuates injury‐induced increases in neuronal clustering and global efficiency; astrocyte activity and topology remain largely unchanged
[31]	Acute brain slice imaging in GFAP–GFP astrocytes	mGluR5	Activation of astrocytic mGluR5 induces intracellular Ca^2+^ oscillations, leading to Ca^2+^‐dependent gliotransmission	mGluR5–dependent gliotransmission induces NMDA receptor–mediated slow inward currents (SICs), causing depolarization and increased firing of medium spiny neurons
[32]	Acute and organotypic hippocampal slices to induce NMDAR–LTD	Not the focus; mGluRs blocked (MCPG) to isolate astrocyte‐driven mechanisms independent of presynaptic mGluR signaling	Astrocytic activation increases intracellular Ca^2+^, leading to Ca^2+^‐dependent glutamate release that activates neuronal NMDA receptors	Astrocyte‐driven gliotransmission induces NMDA receptor–dependent slow inward currents (SICs) and is sufficient to trigger long‐term synaptic depression
[33]	Acute brain slices; dnSNARE transgenic mice	Group I mGluRs	Astrocytic mGluR activation → Ca^2+^ signaling in astrocytic microdomains → SNARE–dependent	Astrocyte‐derived ATP is essential for mGluR–dependent LTD; impairment of gliotransmission reduces LTD, while ATP analog (ATPγS) rescues it
[34]	Acute hippocampal slices; electrophysiology; astrocyte‐specific stimulation via PAR1 activation	Not the focus; astrocytic activation mediated via PAR1 (GPCR), not mGluRs	GPCR (PAR1) activation astrocytic intracellular Ca^2+^ elevation opening of bestrophin‐1 (Best1)	Astrocytic glutamate release enhances NMDA receptor–dependent synaptic plasticity by lowering the threshold for LTP
[35]	Acute mouse hippocampal slices	mGluR5–dependent	Postsynaptic Ca^2+^ rise, nitric oxide (NO) release, astrocyte activation, Ca^2+^‐dependent gliotransmitter release	Astrocytes drive a developmental switch from presynaptic t‐LTD to presynaptic t‐LTP; astrocytic signaling is required for mGluR5–dependent t‐LTP
[36]	Acute hippocampal slices	mGluR1	Astrocytic glutamate transporters (GLT‐1 and GLAST) restrict extracellular glutamate and limit access to perisynaptic mGluR1	Transporter inhibition enhances mGluR1–mediated excitation of O‐LM interneurons; both GLT‐1 and GLAST contribute to restricting receptor activation
[37]	In vivo mouse model of neuropathic pain; somatosensory cortex (S1)	mGluR5	Injury‐induced transient reemergence of astrocytic mGluR5 during the induction phase, enhanced astrocytic Ca^2+^ signaling → Ca^2+^‐dependent upregulation of multiple synaptogenic molecules	Astrocytic mGluR5 initiates and promotes aberrant excitatory synaptogenesis and synaptic remodeling in S1, contributing to persistent neuropathic pain (mechanical allodynia)
[38]	Primary cultured human astrocytes and glioma cells	mGluR3 (Group II); mGluR5 (Group I)	Activation of mGluR3 (via DCG‐IV) upregulates glutamate transporters (GLAST/EAAT1 and GLT‐1/EAAT2)	mGluR3 activation enhances astrocytic glutamate uptake capacity
[39]	Primary cultured rat astrocytes and C6 glioma cells	Group II (mGluR3) and Group III mGluRs	Activation of Group II mGluRs enhances astrocytic glutamate uptake and reduces extracellular glutamate accumulation under neurotoxic conditions	Reduced astrocyte‐mediated glutamate toxicity and attenuation of neuronal apoptosis, indicating a role in neuroprotection
[40]	Mouse cortical neuron–astrocyte mixed cultures and pure astrocyte cultures	Group II mGluRs (mGluR2/3)	Activation of Group II mGluRs induces astrocyte‐dependent release of proteinaceous neuroprotective factors requiring de novo protein synthesis	Conditioned medium from activated astrocytes reduces NMDA–induced neuronal death
[41]	Acute hippocampal slices (mouse CA1)	Group II mGluRs (mGluR2/3; with specific involvement of mGluR3)	Activation of mGluR2/3 induces Ca^2+^‐independent glutamate release from astrocytes via TREK‐1 channels and modulates synaptic glutamate time course	Regulation of synaptic glutamate dynamics and prolongation of excitatory synaptic responses without altering presynaptic release probability

During development, astrocytic mGluR signaling contributes critically to the formation and refinement of synaptic circuits. Kim et al. [42] synthesize evidence demonstrating that astrocytic Group I mGluRs, particularly mGluR5, mediate intracellular Ca^2+^ signaling that underlies neuron–glia communication at the tripartite synapse. During early postnatal development, activation of astrocytic mGluR5 by synaptically released glutamate induces robust somatic Ca^2+^ transients, which in turn promote the release of gliotransmitters and synaptogenic molecules such as thrombospondins (TSP‐1/2), thereby facilitating synapse formation, maturation, and refinement. This mGluR5–dependent Ca^2+^ signaling is markedly downregulated in the mature brain, coinciding with reduced astrocyte‐driven synaptogenesis and stabilization of neural circuits, but can be reactivated under pathological conditions, where reactive astrocytes reexpress mGluR5 and exhibit enhanced Ca^2+^ oscillations. Such re‐emergent signaling has been observed in multiple disease models, including epilepsy, ischemia, AD, and neuropathic pain, where astrocytic mGluR5 activation is associated with altered synaptic remodeling, partly via reexpression and release of synaptogenic factors such as TSP‐1 that can drive excitatory synapse formation and circuit reorganization [42]. Consistent with this view, Falcón‐Moya et al. [35] demonstrate at hippocampal CA3–CA1 synapses that astrocytes are essential for a developmental switch from spike‐timing–dependent LTD (t‐LTD) to presynaptic t‐LTP, providing direct experimental evidence that astrocytes are integral to developmental transitions in plasticity. This switch, observed between postnatal weeks 3 and 5, is characterized by a transition from NMDAR–dependent presynaptic t‐LTD to an mGluR5–dependent, NMDAR–independent presynaptic t‐LTP. t‐LTP induction requires postsynaptic Ca^2+^ influx via L‐type VGCCs (L‐VGCCs) and release from intracellular stores, followed by nitric oxide production as a retrograde messenger, and critically depends on astrocytic Ca^2+^‐dependent signaling and vesicular gliotransmission, as pharmacological inhibition of astrocyte metabolism, intracellular Ca^2+^ buffering, or disruption of vesicular release all abolished t‐LTP [35]. Astrocytes in this context release both adenosine and glutamate, which act synergistically: astrocyte‐derived adenosine activates presynaptic A1 receptors to reduce baseline glutamate release probability, while astrocyte‐derived glutamate activates presynaptic mGluRs necessary for t‐LTP induction, indicating that astrocytes coordinate gliotransmitter release with presynaptic receptor signaling to enable a developmental switch from depression to potentiation [35].

Beyond developmental circuit formation, astrocytic mGluR signaling exerts a sustained influence on synaptic plasticity by directly contributing to, and in some cases being required for, the induction and expression of LTD. Navarrete et al. [32] show in acute and organotypic hippocampal slices that astrocytic signaling is not merely modulatory but required for the induction and expression of NMDAR–dependent LTD at CA3–CA1 synapses. Low‐frequency stimulation elicits robust astrocytic Ca^2+^ elevations accompanied by increased astrocyte‐to‐neuron communication through glutamate release, evidenced by enhanced slow inward currents in CA1 neurons, and interference with astrocytic Ca^2+^ signaling or SNARE–dependent vesicular release significantly impairs LTD without affecting LTP [32]. Optogenetic activation of astrocytes alone is sufficient to induce LTD, which requires postsynaptic NMDAR activation and leads to AMPA receptor internalization, while astrocytic p38α MAPK is identified as a critical mediator whose selective deletion in astrocytes abolishes LTD and prevents LFS–induced increases in astrocytic glutamate release [32]. Complementing this glutamate‐based mechanism, Lalo and Pankratov [33] describe a distinct but convergent astrocytic pathway in which Group I mGluR activation in hippocampal and neocortical slices triggers Ca^2+^ signaling predominantly via mGluR1 in astrocytic microdomains, leading to SNARE–dependent exocytotic ATP release that engages postsynaptic P2X receptors and contributes to mGluR–dependent LTD. Disruption of astrocytic exocytosis or P2 receptor blockade significantly impairs DHPG‐ and paired‐pulse low‐frequency stimulation‐induced LTD, while a nonhydrolyzable ATP analog restores LTD, and paired‐pulse analyses support a predominantly postsynaptic expression mechanism involving AMPA receptor internalization [33]. These findings collectively indicate that astrocytic Group I mGluR signaling, through Ca^2+^‐dependent glutamate and ATP release, constitutes a critical astrocyte‐mediated arm of LTD expression that complements canonical neuronal mechanisms [33].

Astrocytic receptor–driven gliotransmission also modulates synaptic plasticity thresholds and the recruitment of specific glutamate receptor pools, thereby shaping the conditions under which LTP or LTD can occur. Park et al. [34] show that activation of astrocytic G protein–coupled receptors, specifically protease‐activated receptor 1 (PAR1), induces Ca^2+^‐dependent glutamate release through Bestrophin‐1 channels localized at astrocytic microdomains surrounding synapses in hippocampal CA1. PAR1 activation elevates extracellular glutamate in a Best1‐dependent manner and selectively enhances postsynaptic NMDAR activity, particularly GluN2A–containing receptors, without significantly altering AMPAR–mediated currents or presynaptic release probability [34]. This astrocyte‐derived glutamate increases synaptic glutamate concentrations and enhances NMDAR–dependent responses, thereby lowering the threshold for LTP induction and enabling subthreshold stimulation protocols to induce LTP, while attenuating LTD induced by low‐frequency stimulation; these effects are abolished in Best1‐deficient conditions or by NMDAR blockade [34]. In a related context, D’Ascenzo et al. [31] demonstrate in nucleus accumbens slices that activation of astrocytic mGluR5 induces intracellular Ca^2+^ oscillations that trigger glutamate release, leading to NMDAR–dependent slow inward currents in medium spiny neurons. Immunohistochemical analysis localizes mGluR5 predominantly to astrocytic processes, and Group I mGluR stimulation induces astrocytic Ca^2+^ transients that are attenuated by mGluR5 antagonism; photolysis‐induced Ca^2+^ elevations in astrocytes evoke slow inward currents in adjacent neurons, establishing a causal link between astrocytic Ca^2+^ signaling and neuronal excitation [31]. Pharmacological characterization indicates that astrocyte‐derived glutamate preferentially activates NR2B‐containing extrasynaptic NMDARs, distinct from synaptic NR2A–containing receptors, and that neuronal activity recruits astrocytic mGluR5 to generate prolonged Ca^2+^ oscillations and sustained gliotransmission lasting minutes beyond the initial stimulus [31]. This temporally extended astrocytic glutamate release does not directly modulate fast synaptic transmission but provides a parallel, mGluR5–dependent excitatory pathway that can modulate neuronal excitability in a state‐dependent manner, as depolarized “up‐state” potentials enhance the amplitude of slow inward currents and increase action potential firing probability in medium spiny neurons [31]. Together, these studies show that astrocytic GPCR and mGluR5 signaling can tune plasticity by regulating NMDAR activation and the temporal integration of excitatory signals at both synaptic and extrasynaptic sites [31].

Astrocytes also influence neuron–glia plasticity by controlling the spatial profile and duration of glutamate signaling through high‐affinity transporters that constrain mGluR activation on interneurons and other neuronal populations. Huang et al. [36] report that astroglial glutamate transporters tightly regulate mGluR1–mediated slow EPSCs in oriens–lacunosum moleculare interneurons in hippocampal slices, thereby modulating inhibitory circuit dynamics. Pharmacological inhibition of the astrocytic transporter GLT‐1 with dihydrokainate markedly increases mGluR1–dependent slow EPSC amplitude, and broader transporter inhibition with TBOA produces an even larger enhancement, while genetic analyses implicate astrocytic GLT‐1 and GLAST, but not neuronal EAAC1, as the primary determinants of restricted mGluR1 activation. These transporters act as a competitive barrier limiting glutamate diffusion to perisynaptic mGluRs near astrocytic processes, and their inhibition leads to pronounced depolarization and sustained firing of O‐LM interneurons, with subsequent increases in delayed inhibitory postsynaptic currents onto CA1 pyramidal neurons, effects abolished by mGluR1 antagonism [36]. At the level of glutamate homeostasis, Peterson and Binder [43] highlight in a review that astrocytes predominantly express mGluR3 and mGluR5, which differentially regulate glutamate uptake and signaling at the tripartite synapse and are particularly relevant in epilepsy. Astrocytic mGluR5 acutely enhances glutamate uptake by increasing GLT‐1 activity, whereas prolonged activation downregulates GLT‐1 and GLAST, impairing glutamate clearance and potentially elevating neuronal excitability, while mGluR3 activation upregulates GLT‐1 and GLAST expression and enhances glutamate clearance. Both receptors exhibit altered expression patterns in epileptic tissue, with mGluR5 upregulated in reactive astrocytes and mGluR3 showing region‐ and stage‐dependent changes; dysregulated mGluR5 signaling is associated with enhanced astrocytic Ca^2+^ activity, increased gliotransmitter release, and DHPG–induced ictal‐like activity, whereas mGluR3 activation is linked to reduced excitotoxicity [43]. These observations indicate that astrocytic mGluR–transporter coupling is a key determinant of extracellular glutamate dynamics that shapes interneuron‐driven inhibitory plasticity and overall network excitability, particularly under epileptogenic conditions.

Aronica et al. [38] first provided direct evidence that astrocytic Group II mGluRs, specifically mGluR3, exert subtype‐specific control over glutamate transporter expression in human glial cells. Using RT‑PCR and western blot analyses in cultured human astrocytes and glioma cells, they showed that mGluR3 mRNA, but not mGluR2, is expressed in astrocytes, whereas mGluR5 represents the predominant Group I subtype. Functionally, selective activation of Group II mGluRs with DCG‑IV led to a significant upregulation of the astrocytic glutamate transporters GLAST (EAAT1) and GLT‑1 (EAAT2) under growth factor–enriched conditions, while activation of mGluR5 with DHPG produced the opposite effect, reducing transporter expression. These effects were receptor‐specific, as they were blocked by selective antagonists (EGLU for Group II and MPEP for mGluR5). Mechanistically, the DCG‑IV–induced increase in GLT‑1 expression was associated with activation of MAPK/ERK and PI3K/Akt signaling, as indicated by increased phosphorylation of ERK1/2 and Akt and attenuation by pathway inhibitors. Under serum‐free conditions, DCG‑IV selectively enhanced GLT‑1 expression without significantly altering GLAST, pointing to context‐dependent regulation. Overall, this study demonstrates that astrocytic mGluR3 activation positively modulates glutamate transporter expression and thereby supports extracellular glutamate homeostasis, in contrast to the opposing effects mediated by mGluR5 [38].

Building on this transporter‐centered mechanism, Yao et al. [39] examined whether astroglial Group II and III mGluRs can preserve glutamate homeostasis and neuronal viability under toxic stress. In primary cultured rat astrocytes and C6 glioma cells, they found that activation of Group II (DCG‑IV) and Group III (L‑AP4) mGluRs significantly enhanced glutamate uptake that had been reduced by MPP^+^ exposure, a neurotoxin used to model Parkinsonian neurodegeneration. This enhancement was receptor‐specific, as it was abolished by the Group II antagonist APICA and the Group III antagonist MSOP. In parallel, MPP^+^ treatment increased extracellular glutamate and decreased astrocytic uptake capacity, whereas Groups II/III mGluR activation reversed these effects and reduced extracellular glutamate accumulation. Functionally, conditioned medium from MPP^+^‐treated astrocytes induced neuronal apoptosis in midbrain neuron cultures, but this effect was attenuated when astrocytes were pretreated with Groups II or III agonists, indicating a neuroprotective influence mediated via astrocytic mechanisms rather than direct neuronal actions. Consistent with the Aronica et al.’s [38] study, C6 cells expressed mGluR3 mRNA but not mGluR2, supporting a prominent role of mGluR3 within Group II signaling in astroglial cells [39]. Collectively, these findings extend the transporter regulation observed by Aronica et al. [38] to a pathological context, suggesting that astroglial Group II mGluRs, including mGluR3, help maintain glutamate homeostasis and limit excitotoxicity by enhancing uptake under neurotoxic conditions.

Complementing these in vitro observations, Bruno et al. [40] investigated how Group II mGluR activation in mixed cortical neuron–astrocyte cultures contributes to neuroprotection through glial–neuronal interactions using mouse cortical cocultures. They showed that selective Group II agonists (DCG‑IV, 4C3HPG, and L‑CCG‑I) significantly reduced neuronal death induced by a brief NMDA exposure, and that this effect was abolished by the Group II antagonist PCCG‑IV, confirming receptor specificity. Neuroprotection was observed not only during coapplication with NMDA but also when agonists were applied 6–20 h before the excitotoxic insult, indicating a delayed and sustained mechanism that required new protein synthesis, as shown by its attenuation with cycloheximide. Immunocytochemistry revealed mGlu2/3 expression in both neurons and astrocytes, but functional experiments in pure astrocyte cultures demonstrated that conditioned medium collected 2–20 h after transient Group II agonist exposure conferred significant protection when transferred to NMDA–treated mixed cultures. This effect was absent when conditioned medium was collected immediately after agonist application or when protein synthesis was inhibited, and it was lost upon heat treatment, consistent with involvement of astrocyte‑derived, heat‑labile protein factors [40]. Together with the uptake‐enhancing actions reported by Aronica et al. [38] and Yao et al. [39], these results support a model in which astrocytic Group II mGluR activation, likely including mGluR3, contributes to neuroprotection through both improved glutamate handling and the production of astrocyte‐derived protective signals that require de novo protein synthesis.

More recently, Roh et al. [41] focused on how astrocytic Group II mGluRs, particularly mGluR3, influence the temporal profile of synaptic glutamate at hippocampal CA1 synapses. Using electrophysiology, pharmacology, and glutamate imaging, they showed that activation of mGluR2/3 with LY379268 significantly prolonged the decay time course of AMPAR–mediated EPSCs in the presence of cyclothiazide, indicating altered glutamate kinetics at the synapse. This effect occurred without changes in presynaptic release probability or postsynaptic receptor properties, as paired‐pulse ratio and mEPSC parameters were unchanged, suggesting a nonneuronal origin. Pharmacological blockade of mGluR2/3 with LY341495 abolished PAR1‑mediated modulation of synaptic glutamate levels, and shRNA–mediated knockdown of mGluR3 similarly prevented the reduction in kynurenate sensitivity, implicating mGluR3 specifically in this process. Using iGluSnFR–based glutamate imaging in cultured astrocytes and slices, the study further showed that mGluR2/3 activation induces rapid glutamate release from astrocytes without a detectable rise in intracellular Ca^2+^, and that this release is reduced by knockdown of the TREK‑1 channel. Collectively, these findings indicate that astrocytic mGluR2/3, and mGluR3 in particular, can also regulate the temporal profile of synaptic glutamate through a TREK‑1‑dependent, Ca^2+^‑independent glutamate release mechanism, complementing the transporter‐based modulation and neuroprotective actions described in earlier studies [41].

At the level of network organization, astrocytic mGluR signaling contributes to the coordination of neuron–glia functional connectivity and its reconfiguration after injury. Schroeder et al. [30] use mixed cortical neuron–astrocyte cultures with high‐speed calcium imaging and multilayer network modeling to investigate how mGluR5–dependent signaling influences neuron–astrocyte connectivity under basal conditions and following mechanical injury. mGluR5 is expressed in both neurons and astrocytes, with lower detectable intensity in astrocytes, and is positioned within the tripartite synapse to detect synaptic glutamate, trigger Ca^2+^ elevations, and promote gliotransmitter release that modulates presynaptic release probability [30]. Pharmacological inhibition of mGluR5 reduces neuronal activity without altering astrocytic Ca^2+^ event rates, suggesting differential sensitivity of neuronal versus astrocytic activity to receptor blockade, while network analysis indicates that mechanical injury increases clustering and efficiency in neuronal networks without significantly changing astrocytic network topology [30]. Pre‐inhibition of mGluR5 attenuates injury‐induced alterations in neuronal network organization, and multilayer network analysis shows that neuron–astrocyte functional connectivity is not strictly defined by spatial proximity, with astrocytic processes participating in distinct functional communities independent of their parent cell, indicating compartmentalized astrocytic signaling within neuron–glia networks [30]. These findings support the view that astrocytic mGluR5 signaling operates through Ca^2+^‐dependent gliotransmission to influence neuronal network dynamics and plasticity‐related reconfiguration after injury, even when astrocytic network topology per se remains relatively stable.

Finally, pathological conditions can reactivate developmentally downregulated astrocytic mGluR pathways to drive maladaptive neuron–glia plasticity and persistent circuit dysfunction. Danjo et al. [37] show in a partial sciatic nerve ligation model that mGluR5, largely absent in healthy adult astrocytes, is transiently re‐expressed in astrocytes of the contralateral primary somatosensory cortex during the early induction phase of neuropathic pain, in a temporally restricted window approximately 3–7 days postinjury. Astrocytic mGluR5 activation in this period drives robust Ca^2+^ signaling that is abolished by pharmacological inhibition or astrocyte‐specific mGluR5 deletion and induces expression of synaptogenic molecules such as thrombospondin‐1, Glypican‐4, and Hevin, which are upregulated in wild‐type but not in astrocyte‐specific mGluR5 KO mice [37]. These molecules promote excessive excitatory synapse formation, as indicated by increased colocalization of presynaptic and postsynaptic markers in S1 cortex, and are associated with sustained increases in neuronal activity, reflected by elevated c‐fos expression up to 1 month after injury, despite the transient mGluR5 expression [37]. Behavioral analyses show that astrocyte‐specific deletion of mGluR5 abolishes nerve injury‐induced mechanical allodynia, supporting a causal relationship between astrocytic mGluR5 signaling, synaptic reorganization, and pain‐related network dysfunction [37]. Together with the developmental and epilepsy‐related data discussed above, these findings support a model in which astrocytic mGluR1/5 and mGluR3 signaling, coupled to Ca^2+^‐dependent gliotransmission and glutamate transport, bidirectionally regulate synaptic strength and network excitability, and can be reengaged in adult pathology to drive large‐scale synaptic remodeling and persistent changes in neuron–glia plasticity.

A coherent body of work now indicates that astrocytic mGluRs are not passive modulators but active determinants of synaptic and network plasticity; however, several conceptual and technical limitations constrain how firmly these conclusions can be generalized, and they define clear priorities for future research. Collectively, the studies summarized in Section 5 and in Table 3 converge on the view that Group I receptors (mGluR1/5) orchestrate Ca^2+^‐dependent gliotransmission and synaptogenic programs that are most prominent during development but can be pathologically reengaged in the adult brain, whereas Group II/III receptors, especially mGluR3, primarily support glutamate homeostasis, neuroprotection, and the fine‐tuning of synaptic glutamate kinetics via transporter regulation and TREK‑1–dependent, Ca^2+^‑independent release. This dualistic framework provides a mechanistic explanation for how astrocytes can bidirectionally regulate LTP/LTD thresholds, interneuron‐driven inhibitory plasticity, and large‐scale circuit remodeling after injury, and it highlights astrocytic mGluRs as attractive, glia‐specific therapeutic entry points to restore excitatory–inhibitory balance in epilepsy, neuropathic pain, and neurodegenerative disease. At the same time, most available data derive from acute or organotypic rodent slice preparations, simplified cocultures, or reductionist injury and toxin models that only partially recapitulate the spatiotemporal complexity, species specificity, and chronicity of human CNS disorders; in addition, many studies rely on global pharmacological manipulations or non‐cell‐type‐specific genetic tools that cannot fully disentangle astrocytic from neuronal mGluR contributions, nor resolve astrocyte heterogeneity across brain regions and disease stages. Another major limitation is that current work typically interrogates single receptor subtypes or signaling arms in isolation, whereas in vivo astrocytes integrate convergent GPCR inputs (for example, PAR1, μ‐opioid receptors, and mGluR2/3) and neuromodulatory pathways, engage both vesicular and channel‐mediated glutamate release mechanisms (Best1, TREK‑1, and SWELL1), and undergo dynamic transcriptional and metabolic reprograming that is only beginning to be mapped at single‐cell resolution. In the future, a key priority will be to combine cell‐type‐ and even subcellular‐compartment‐specific genetic tools, high‐speed in vivo Ca^2+^ and glutamate imaging, and multilayer network analyses to define how astrocytic mGluR1/5 and mGluR3 signaling operate within intact circuits over developmental and disease time scales, including sex‐ and region‐specific effects that may underlie differential vulnerability of structures such as hippocampus, anterior cingulate cortex (ACC), and primary somatosensory cortex in epilepsy, chronic pain, and affective disorders. Parallel efforts are needed to systematically profile downstream signaling hubs (for example, MAPK/ERK, PI3K/Akt, p38 MAPK, and transcriptional programs) engaged by astrocytic mGluRs in human iPSC–derived astrocytes and ex vivo human tissue and to integrate these pathways with astrocyte metabolic and redox states that are increasingly recognized as modulators of neuron–glia interactions and plasticity. Finally, translational studies must address the gap between promising preclinical data and the modest clinical performance of mGluR–targeting drugs by developing astrocyte‐biased allosteric modulators, ligand–antibody or gene‐therapy approaches that restrict mGluR5 or mGluR3 manipulation to defined astrocyte populations, and biomarker strategies (for example, imaging or CSF signatures of astrocytic activation) that allow patient stratification and on‐target monitoring in vivo, thereby enabling the rational design of glia‐centered interventions that harness or normalize astrocytic mGluR signaling to restore synaptic integrity and prevent network destabilization in CNS disease.

## 6. Classical Ionotropic Plasticity

Classical ionotropic synaptic plasticity emerges from the interplay between fast glutamatergic transmission and activity‐dependent receptor modulation, providing the canonical framework within which slower, modulatory mGluR–dependent forms of plasticity are engaged. In contrast to mGluRs, iGluRs such as AMPA and NMDARs mediate rapid excitatory postsynaptic currents and act as primary detectors and transducers of brief synaptic events, with their subunit composition, conductance properties, and trafficking dynamics critically shaping the amplitude and temporal profile of synaptic responses. Within this framework, AMPA receptors are chiefly responsible for generating fast depolarization, whereas NMDARs serve as coincidence detectors that couple patterns of synaptic activity to Ca^2+^‐dependent intracellular cascades, thereby providing the classical entry point for LTP and LTD induction. However, the contribution of iGluRs to plasticity extends beyond simple ion flux: subunit‐specific organization, nanoscale localization, and scaffold‐mediated signaling complexes collectively determine how ionotropic signaling is integrated over space and time and how it ultimately intersects with metabotropic pathways.

The study by Reiner et al. [44] illustrates these principles by leveraging optical tools to probe the functional diversity and subcellular distribution of both ionotropic and mGluRs within synaptic microdomains. Using photo‐uncaging and photoswitchable ligands, the authors show that iGluR activation occurs on millisecond timescales and can be spatially confined to pre‐, post‐, or extrasynaptic compartments, emphasizing that classical fast transmission is implemented within a highly compartmentalized synaptic architecture. This work further demonstrates that receptor subunit diversity and heteromerization, together with precise spatial targeting, contribute to variability in synaptic responses and complicate the interpretation of traditional pharmacological manipulations, thereby providing a mechanistic basis for how local ionotropic signaling sets the initial conditions for downstream plasticity processes. By establishing that both ionotropic and metabotropic receptors are embedded in a shared but spatially structured environment, this study helps bridge classical iGluR–based models of plasticity with the broader mGluR signaling framework developed in subsequent sections [44]. Building on this synaptic‐scale view, work by Kita et al. [45] demonstrates how AMPA receptor subunit composition, particularly the presence of GluA4, governs synaptic strength and information processing in defined cerebellar circuits. Using GluA4 KO mice, the authors report an approximately 80% reduction in excitatory postsynaptic currents at mossy fiber–granule cell synapses, indicating that GluA4–containing AMPARs are major determinants of fast excitation at these inputs, whereas other cerebellar synapses, such as parallel fiber–Purkinje cell and mossy fiber–Golgi cell synapses, are relatively spared. Despite partial compensatory adjustments, including reduced tonic inhibition and enhanced NMDAR–mediated currents, synaptic efficacy, spike fidelity, and temporal precision during high‐frequency stimulation remain impaired, leading to diminished expansion coding in granule cell networks and slower associative learning in behavioral assays. These findings link subunit‐specific AMPAR composition to synaptic reliability and circuit‐level computation, illustrating how modifications in ionotropic receptor makeup can alter the substrates upon which LTP/LTD and, by extension, mGluR–dependent metaplasticity operate [45].

At the level of individual receptor channels, Iacobucci and Popescu [46] refine the classical view of NMDAR function by demonstrating that extracellular Ca^2+^ not only permeates the channel but also modulates its conductance properties through ion–ion interactions. Using single‐channel electrophysiology, the authors show that NMDARs exhibit reduced unitary conductance in the presence of extracellular Ca^2+^, attributable to a voltage‐independent channel block in which Ca^2+^ partially impedes Na^+^ flux via competition within the pore rather than classical occlusion. Voltage‐ramp and voltage‐step protocols reveal that Ca^2+^ influences both conductance states and open probability, indicating that the magnitude and temporal profile of NMDAR–mediated currents are dynamically shaped by extracellular ionic conditions. This work complements circuit‐level studies by highlighting that the Ca^2+^ signals driving downstream plasticity pathways are not fixed properties of the receptor but depend on the local ionic milieu, thereby introducing another layer of regulation through which classical ionotropic mechanisms interface with intracellular cascades engaged by mGluRs [46]. These biophysical and subunit‐specific insights converge in the review by Youn et al. [47] which synthesizes the roles of iGluRs and VGCCs in the induction and expression of LTP, particularly in spinal dorsal horn circuits. The authors describe how AMPA receptors provide the initial depolarization necessary to relieve the voltage‐dependent Mg^2+^ block of NMDARs, allowing Ca^2+^ influx that initiates kinase activation, receptor phosphorylation, and activity‐dependent AMPAR trafficking to the postsynaptic membrane. They further emphasize that Ca^2+^ entry via VGCCs, including L‐type and N‐type channels, can complement or, under certain conditions, partially substitute for NMDAR–mediated Ca^2+^ in both induction and maintenance phases of LTP. In this model, the kinetics and distribution of AMPA, NMDA, and kainate receptors, together with VGCCs, yield temporally structured Ca^2+^ signals that govern the threshold and direction of synaptic strength changes, providing a canonical framework within which receptor trafficking, scaffold interactions, and metabotropic modulation can act [47].

Finally, the study by Zheng and Keifer [48] links ionotropic receptor function to activity‐dependent structural remodeling by dissecting the mechanisms of subunit‐specific AMPAR trafficking during associative learning, with a focus on the scaffolding protein SAP97. In an in vitro model of classical conditioning, the authors identify a temporally ordered, two‐stage process in which PKA–dependent formation of a SAP97–AKAP/PKA–GluA1 complex promotes early insertion of GluA1–containing AMPARs into the PSD via interactions with PSD95, followed by a later phase in which GluA4–containing AMPARs are delivered through a SAP97–KSR1/PKC–GluA4 complex linked to PKC and ERK activation. Disruption of SAP97 expression reduces both PSD95 association and surface expression of GluA1 and GluA4, while imaging shows increased colocalization of these subunits with SAP97 and PSD95 during conditioning, supporting a model in which scaffold‐assembled kinase complexes coordinate subunit‐specific AMPAR delivery to synapses. This temporally structured trafficking provides a mechanistic substrate for the expression and consolidation of LTP–like changes and illustrates how classical ionotropic plasticity is embedded within a broader scaffold‐ and kinase‐dependent signaling architecture that can be modulated by mGluR activation in subsequent sections [48]. Taken together, these studies support a view of classical ionotropic plasticity in which fast iGluR–mediated transmission, subunit‐specific receptor composition, dynamic channel biophysics, and scaffold‐dependent trafficking converge to generate the Ca^2+^ signals and receptor redistribution that underlie canonical LTP and LTD. At the same time, several limitations qualify this framework: most evidence derives from reduced preparations, specific brain regions, and genetically or pharmacologically manipulated systems, which may not fully capture how ionotropic mechanisms operate within intact, behaving networks or across diverse cell types and developmental stages. In addition, current data only partially resolve how variability in extracellular ionic milieu, receptor subunit expression, and scaffold availability shapes synapse‐to‐synapse heterogeneity in plasticity rules, and they often stop short of directly linking ionotropic changes to defined patterns of mGluR–dependent plasticity in vivo. Future work combining cell type–specific genetic strategies, high‐resolution imaging of receptor nanodomains and trafficking, and in vivo recordings during physiologically relevant learning paradigms will be important to clarify how ionotropic receptor dynamics set the boundary conditions for mGluR signaling, metaplasticity, and neuron–glia interactions. By systematically integrating ionotropic and metabotropic contributions at molecular, synaptic, and circuit levels, such studies are expected to refine models of plasticity induction and expression and to better inform therapeutic strategies that aim to modulate mGluR–dependent processes without disrupting the core ionotropic mechanisms that maintain baseline synaptic transmission.

## 7. mGluR–Mediated Synaptic Plasticity

**Figure 1:**
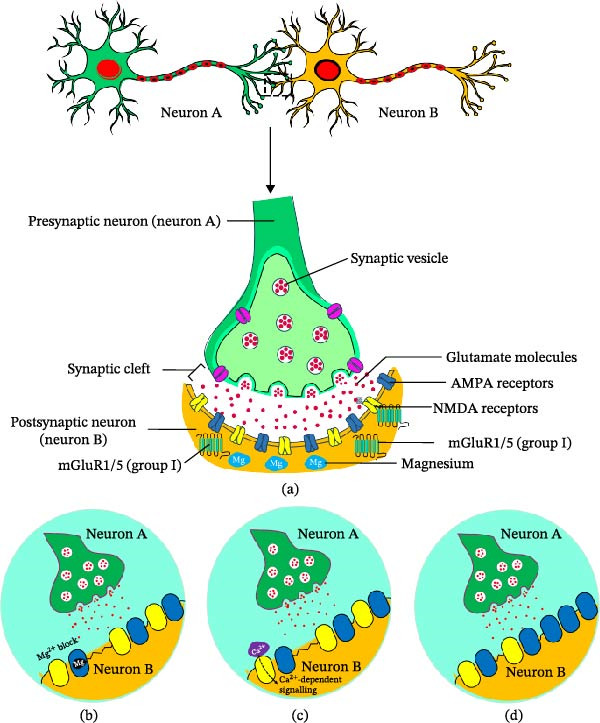
Synaptic transmission and long‐term potentiation (LTP) mechanisms. (a) Schematic representation of synaptic communication, where the axon terminal of neuron A (presynaptic neuron) forms a synapse with the dendrite of neuron B (postsynaptic neuron). (b) Upon activation of neuron A, glutamate is released into the synaptic cleft, activating postsynaptic AMPA receptors, which generate membrane depolarization, and engaging perisynaptic metabotropic glutamate receptors (mGluRs; particularly Group I mGluR1/5), which initiate intracellular signaling cascades. The resulting depolarization relieves the voltage‐dependent Mg^2+^ block of NMDA receptors. (c) Continued glutamate release from the same presynaptic terminal activates NMDA receptors, allowing Ca^2+^ influx into the postsynaptic neuron. The increase in intracellular Ca^2+^ triggers downstream signaling pathways that promote the recruitment and insertion of additional AMPA receptors into the postsynaptic membrane. (d) The increased density of AMPA receptors strengthens synaptic transmission, leading to enhanced synaptic potentiation (long‐term potentiation [LTP]) upon subsequent presynaptic stimulation.

mGluRs play a central role in regulating synaptic plasticity by linking extracellular glutamatergic signaling to intracellular biochemical pathways that shape neuronal function. Unlike iGluRs, mGluRs are G protein–coupled receptors distributed across both pre‐ and postsynaptic compartments, where their localization critically determines their functional impact on neurotransmitter release, dendritic excitability, and synaptic strength. Based on sequence homology and signaling properties, mGluRs are classified into three groups (I–III), each engaging distinct intracellular cascades, including Gq/11–mediated activation of PLC and Ca^2+^ mobilization (Group I) or Gi/o–mediated inhibition of cAMP signaling (Groups II/III). These signaling pathways interact with key regulators of synaptic plasticity, such as NMDARs, voltage‐sensitive Ca^2+^ channels (VSCCs), and intracellular Ca^2+^ stores, thereby influencing the induction and maintenance of LTP and LTD. The coordinated interaction between ionotropic and mGluRs during synaptic plasticity is illustrated in Figure 1. In this framework, glutamate released from a single presynaptic terminal activates postsynaptic AMPA receptors to generate rapid depolarization, while simultaneously engaging perisynaptic mGluRs that initiate slower intracellular signaling cascades. The resulting depolarization relieves the voltage‐dependent Mg^2+^ block of NMDARs, permitting Ca^2+^ influx into the postsynaptic neuron. Importantly, mGluRs do not directly mediate fast excitatory transmission but instead modulate intracellular signaling, receptor trafficking, and plasticity thresholds, thereby shaping the induction and persistence of LTP and LTD. Furthermore, emerging evidence indicates that mGluR–dependent plasticity is not solely determined by the magnitude of Ca^2+^ influx but rather by the spatial and temporal coordination of multiple signaling pathways and receptor subtypes. In this context, understanding the subtype‐specific distribution and intracellular mechanisms of mGluR signaling is essential for interpreting their roles in synaptic plasticity and their broader implications in neurological disorders. To improve clarity and systematically address these aspects, the key features of representative studies, including mGluR subtype specificity, receptor localization, pharmacological modulators, intracellular signaling pathways, and associated plasticity outcomes, are summarized in Table 4. The following studies provide a mechanistically organized overview of how distinct mGluR subtypes regulate synaptic plasticity through coordinated intracellular signaling networks. In particular, Group I mGluRs (mGluR1 and mGluR5) are predominantly postsynaptic and regulate Ca^2+^‐dependent signaling and LTP induction, whereas Groups II and III receptors are largely presynaptic and modulate neurotransmitter release and plasticity thresholds.

**Table 4:** mGluR subtype‐specific mechanisms underlying synaptic plasticity.

Study	Brain region/model	mGluR subtypes	Receptor localization	Agonist/antagonist	Intracellular mechanism	Plasticity outcome
[49]	Rat hippocampus (CA2; SC–CA2, EC–CA2)	Group III (mGluR4, mGluR7 predominant; mGluR6, mGluR8 lower)	Classically presynaptic; however, no presynaptic changes were detected, and plasticity is mechanistically postsynaptic	(RS)‐CPPG, UBP1112	Group III mGluR inhibition increases ERK/MAPK phosphorylation and reduces STEP, leading to protein synthesis–dependent signaling; requires NMDA receptors	Enables late‐LTP at SC‐CA2 (normally resistant) and supports STC, converting EC‐CA2 early‐LTP into late‐LTP
[50]	Rat hippocampus (CA3–CA1 Schaffer collateral synapses; CA1 pyramidal neurons)	Group I mGluR1 (mGluR5 not required)	Postsynaptic (dendritic spines; perisynaptic)	DHPG: used in mechanistic assays (not for LTP induction) Antagonists: YM298198 (mGluR1), MPEP (mGluR5), L‐689560 (NMDAR), Nimodipine/Mibefradil/Ni^2+^ (VSCC blockers)	mGluR1 activation inhibits SK channels, relieving NMDAR inhibition and enhancing function; LTP requires sequential Ca^2+^ entry via NMDARs then VSCCs, not total Ca^2+^ level	Hebbian LTP requires mGluR1, NMDARs, and VSCCs; shows modified STDP‐ needs postsynaptic bursts, and anti‐causal pairing does not induce LTD
[51]	Rat hippocampal slice cultures (Wistar); TPC1/TPC2 knockout experiments in mice	Group I mGluR1 (mGluR5 not involved)	Postsynaptic (dendritic spines; microdomain coupling between acidic stores and ER)	Agonists/tools: glutamate; NAADP‐AM (↑ intracellular NAADP) Antagonists: JNJ16259685, LY341495, Ned‐19, ryanodine, GPN, bafilomycin A1, okadaic acid	mGluR1 → NAADP–mediated Ca^2+^ release (acidic stores via TPC1/2) → RyR amplification → SK channel inhibition → depolarization; IP_3_–independent, microdomain signaling	mGluR1–dependent LTP (STDP) requires NAADP signaling, acidic store Ca^2+^ release (TPC1/2), and SK channel inhibition; loss of TPCs shifts LTP to LTD
[52]	Mouse primary hippocampal neurons (cultured; glutamate stimulation and bicuculline‐induced network activity)	Not directly tested (mGluR involvement inferred from prior literature)	Not determined	Agonist: glutamate; Antagonists: Ned‐19, BZ194; bafilomycin A1; bicuculline	Glutamate triggers intracellular Ca^2+^ rise partly via NAADP, involving acidic store release; TPC1/2 and TRPML1 are suggested, not directly tested	Modulates Ca^2+^ dynamics (not LTP/LTD): NAADP antagonists reduce amplitude/AUC of glutamate Ca^2+^ signals and increase frequency but lower amplitude of bicuculline‐induced oscillations
[53]	Rat nucleus accumbens (acute slices, in vivo infusion, immunohistochemistry)	mGluR5 (Group I)	Predominantly cytoplasmic in neuronal soma with colocalization with PKCε; surface versus intracellular pools assessed via biotinylation (not spatially resolved)	Agonist: DHPG; PKCε activator: Tat‐ψεRACK; PKCε inhibitors: εV1‐2, Myr‐εV1‐2	mGluR5 (Gq/11→PLC) increases PKCε (Ser729) and ERK1/2 phosphorylation; PKCε regulates trafficking‐activation promotes internalization, inhibition increases surface expression; phosphorylation‐dependent	No direct LTP/LTD; activity‐dependent mGluR5 trafficking‐DHPG induces internalization; PKCε activation promotes internalization, inhibition increases surface expression; relevant to adaptive/addiction plasticity
[54]	Rat nucleus accumbens (NAc); in vivo microdialysis, cocaine self‐administration and reinstatement models; CB1‐KO mice	mGluR5 (Group I)	Primarily postsynaptic; localized in striatal medium spiny neurons (MSNs), not astrocytes	Antagonist: MPEP; CB1 antagonist: SR141716A (rimonabant); blockers: ω‐conotoxin GVIA (N‐type Ca^2+^ channel), TTX	mGluR5 is tonically active in MSNs; MPEP reduces eCB‐CB1 signaling, increasing glutamate release; this activates presynaptic mGluR2/3, suppressing cocaine‐induced glutamate signaling	Reduces cocaine self‐administration and reinstatement; increases basal glutamate; attenuates cocaine‐evoked glutamate via mGluR2/3 feedback; no direct LTP/LTD measured

At a functional level, mGluR–mediated plasticity emerges from the coordinated actions of distinct receptor subtypes that operate at different synaptic compartments. Presynaptic receptors primarily regulate neurotransmitter release probability, whereas postsynaptic receptors control intracellular Ca^2+^ dynamics and downstream signaling pathways that determine plasticity outcomes. At the network level, one way in which mGluRs influence synaptic plasticity is by setting the baseline threshold conditions under which LTP can be expressed. Dasgupta et al. [49] investigated the role of Group III mGluRs in regulating synaptic plasticity in the hippocampal CA2 region, with a particular focus on receptor distribution, subtype contribution, and intracellular signaling mechanisms. The study demonstrated that Group III mGluRs, predominantly mGluR4 and mGluR7, are highly expressed in CA2, with a largely presynaptic localization where they negatively regulate neurotransmitter release via Gi/o protein–mediated inhibition of the cAMP signaling pathway. Using pharmacological antagonists ((RS)‐CPPG and UBP1112), the authors showed that inhibition of Group III mGluRs enables the induction of NMDAR–dependent and protein synthesis–dependent LTP at SC–CA2 synapses, which are otherwise resistant to canonical LTP. Importantly, this effect was not attributed to enhanced presynaptic release but rather linked to postsynaptic mechanisms, including activation of ERK/MAPK signaling and concomitant downregulation of STEP, a phosphatase known to negatively regulate ERK activity. Furthermore, Group III mGluR inhibition supported synaptic tagging and capture (STC), allowing weakly stimulated EC–CA2 synapses to express persistent LTP when paired with strong SC–CA2 stimulation, indicating a role in associative plasticity. Collectively, the findings suggest that Group III mGluRs (particularly mGluR4 and mGluR7) act as modulatory gates that constrain LTP induction in CA2 through suppression of cAMP–dependent and ERK–mediated intracellular pathways, thereby influencing both the threshold and persistence of synaptic plasticity [49].

In contrast to this presynaptic gating by Group III receptors, several studies have highlighted how postsynaptic Group I mGluRs shape the induction and expression of LTP by coordinating distinct Ca^2+^ sources and ion channel modulators at hippocampal synapses. Tigaret et al. [50] investigated the mechanisms underlying spike‐timing–dependent plasticity (STDP) at hippocampal SC–CA1 synapses, with a specific focus on the role of Group I mGluRs, particularly mGluR1, in coordinating intracellular Ca^2+^ signaling. The study demonstrates that mGluR1 is postsynaptically localized in dendritic spines and is activated during coincident pre‐ and postsynaptic activity, where it modulates synaptic function through Gq/PLC–dependent signaling pathways. Functionally, mGluR1 activation was found to be essential for LTP induction, as selective pharmacological blockade with YM298198 abolished LTP, whereas inhibition of mGluR5 had no effect. Mechanistically, mGluR1 does not directly contribute to fast Ca^2+^ transients but instead facilitates LTP indirectly by enhancing NMDAR function through inhibition of SK (small conductance Ca^2+^‐activated K^+^) channels, thereby removing a negative feedback constraint on NMDAR–mediated Ca^2+^ influx. In addition, LTP induction required the coordinated activation of multiple Ca^2+^ sources, including NMDARs and VSCCs, rather than being determined solely by the magnitude of Ca^2+^ transients within dendritic spines. Notably, the study shows that the amplitude of spine Ca^2+^ signals does not correlate with the direction or magnitude of synaptic plasticity, indicating that temporal coordination and source‐specific Ca^2+^ signaling are more critical determinants. Together, these findings establish that mGluR1 acts as a modulatory regulator of Hebbian plasticity by facilitating NMDAR function and integrating multiple intracellular signaling pathways that govern LTP induction at hippocampal synapses [50].

Building on the concept that Group I mGluRs integrate distinct Ca^2+^ sources, Foster et al. [51] investigated the role of the Group I mGluR mGluR1 in hippocampal synaptic plasticity, with particular emphasis on its coupling to intracellular Ca^2+^ signaling from acidic organelles. The study demonstrates that mGluR1, primarily localized postsynaptically in CA1 pyramidal neurons, selectively engages a nicotinic acid adenine dinucleotide phosphate (NAADP)–dependent pathway that mobilizes Ca^2+^ from endolysosomal (acidic) stores via two‐pore channels (TPC1/2). This signaling is distinct from classical IP_3_–mediated pathways, as mGluR1–induced Ca^2+^ transients were dependent on NAADP and ryanodine receptor‐mediated amplification but not on IP_3_ receptors. Mechanistically, the released Ca^2+^ modulates dendritic excitability by transiently inhibiting SK‐type K^+^ channels through a process involving protein phosphatase 2A (PP2A), thereby facilitating membrane depolarization. Functionally, this cascade was shown to be essential for mGluR1–dependent LTP, as disruption of NAADP signaling or genetic deletion of TPCs abolished LTP and, notably, shifted synaptic plasticity toward LTD under the same stimulation conditions. These findings indicate that mGluR1–dependent recruitment of acidic Ca^2+^ stores constitutes a critical intracellular mechanism that determines both the induction and polarity of synaptic plasticity, highlighting a specific receptor–organelle coupling that regulates LTP versus LTD outcomes in hippocampal neurons [51].

Consistent with this, Hermann et al. [52] investigated the contribution of NAADP–dependent signaling to glutamate‐evoked Ca^2+^ dynamics in primary mouse hippocampal neurons, with particular emphasis on intracellular Ca^2+^ store mobilization and its interaction with glutamatergic receptor pathways. The study indicates that activation of mGluRs, most likely mGluR1, based on prior evidence, stimulates NAADP production, which in turn triggers Ca^2+^ release from acidic endolysosomal compartments via two‐pore channels (TPC1/2) and TRPML1 channels, as summarized in the mechanistic model. Pharmacological inhibition of NAADP signaling using Ned‐19 and BZ194 significantly reduced the amplitude and overall magnitude (AUC) of glutamate‐induced Ca^2+^ transients, demonstrating that NAADP–sensitive intracellular stores contribute substantially to cytosolic Ca^2+^ elevations. Additionally, these antagonists altered the temporal properties of Ca^2+^ signaling during bicuculline‐induced endogenous glutamate release, increasing oscillation frequency while reducing transient amplitude, indicating a role for NAADP in shaping Ca^2+^ signal dynamics. Gene expression analyses further supported the presence of NAADP target channels, including TPC1, TPC2, and TRPML1, in hippocampal neurons, reinforcing the relevance of acidic Ca^2+^ stores in this process. Although the study does not directly measure synaptic plasticity outcomes, it references prior evidence linking NAADP–TPC signaling to shifts between LTP and LTD, suggesting that NAADP–mediated Ca^2+^ release may influence synaptic plasticity by modulating intracellular Ca^2+^ homeostasis and signal patterns rather than acting as a primary determinant of plasticity induction [52].

Beyond the hippocampus, mGluR–mediated plasticity is also shaped by subtype‐specific signaling and trafficking mechanisms in striatal circuits, where Group I mGluRs regulate both postsynaptic receptor availability and presynaptic neurotransmitter release. Schwendt and Olive [53] examined the regulation of Group I mGluR mGluR5 in the rat nucleus accumbens (NAc), focusing on its subcellular distribution and intracellular signaling mechanisms mediated by protein kinase C epsilon (PKCε). mGluR5, a Gq/11–coupled receptor, was shown to colocalize with PKCε in a subset of NAc neurons, indicating spatial proximity for functional interaction. Mechanistically, activation of mGluR5 with the selective agonist DHPG induced phosphorylation of PKCε and ERK1/2, consistent with engagement of PLC–dependent signaling cascades and downstream kinase activation. Importantly, PKCε activity was found to regulate mGluR5 trafficking: activation of PKCε promoted receptor internalization (reduced surface expression and increased intracellular localization), whereas inhibition of PKCε translocation increased surface expression of mGluR5 under basal conditions. These findings demonstrate that mGluR5 signaling is dynamically controlled by PKCε–dependent phosphorylation and trafficking processes, which determine receptor availability at the neuronal membrane. Although the study does not directly measure LTP or LTD, the identified ERK1/2 activation and receptor trafficking mechanisms are well‐established modulators of synaptic strength, suggesting that PKCε–mGluR5 interactions may influence synaptic plasticity by regulating receptor surface expression and downstream signaling capacity in striatal circuits [53].

Complementing these trafficking‐based mechanisms, Li et al. [54] investigated the role of the Group I mGluR mGluR5 in the nucleus accumbens (NAc), focusing on its cellular localization, intracellular signaling, and regulation of glutamatergic transmission in the context of cocaine‐related behaviors. The study shows that mGluR5 is predominantly localized postsynaptically on striatal medium spiny neurons rather than astrocytes, where it couples to Gq/11 proteins and activates the PLC–IP_3_/DAG–PKC signaling cascade. Functionally, pharmacological blockade of mGluR5 using the selective antagonist MPEP resulted in a significant increase in extracellular glutamate levels in the NAc without altering dopamine, indicating a selective modulation of glutamatergic transmission. Mechanistically, this effect was shown to depend on a retrograde eCB signaling pathway: inhibition of postsynaptic mGluR5 reduces eCB release, leading to decreased activation of presynaptic CB1 receptors and consequent disinhibition of Ca^2+^‐dependent glutamate release. This interpretation is supported by the finding that CB1 receptor antagonism mimicked, and CB1 receptor deletion abolished, the effects of MPEP on glutamate release. Additionally, MPEP attenuated cocaine‐induced increases in extracellular glutamate, suggesting a regulatory role in activity‐dependent glutamate signaling. Although the study does not directly assess LTP or LTD, the described mGluR5–eCB–CB1 signaling axis represents a well‐established mechanism underlying presynaptic forms of LTD, indicating that mGluR5 can modulate synaptic plasticity by controlling retrograde signaling and presynaptic neurotransmitter release probability in striatal circuits [54].

Collectively, these findings demonstrate that mGluR–dependent synaptic plasticity is governed by the integration of subtype‐specific receptor localization with distinct intracellular signaling pathways across brain regions, spanning Gi/o–cAMP–dependent presynaptic gating in CA2, Gq/PLC–SK channel modulation and NAADP–dependent acidic store Ca^2+^ release in CA1, and PKCε–dependent trafficking and eCB–CB1–mediated retrograde signaling in nucleus accumbens circuits. A key limitation is that each study typically focuses on a restricted set of synapses, cell types, or receptor subtypes, often under ex vivo conditions, which constrains the generalizability of these mechanisms across different brain regions, developmental stages, and behavioral states. Moreover, most work dissects individual pathways (for example, NAADP–TPC signaling or PKCε–dependent mGluR5 trafficking) in isolation, leaving open how these cascades interact with other modulators such as gene transcription programs, protein synthesis, and disease‐associated alterations in mGluR signaling that have been implicated in neurodegeneration and addiction. Future studies need to integrate multiscale approaches combining subtype‐specific pharmacology, genetic manipulation, and high‐resolution imaging in vivo to map how mGluR1, mGluR5, and Group III receptors dynamically coordinate Ca^2+^ sources, receptor trafficking, and retrograde messengers to bias the threshold, polarity, and persistence of LTP and LTD in circuit‐ and disease‐specific contexts. Such work may clarify how targeting defined receptor–organelle couplings or downstream signaling nodes can be leveraged to normalize aberrant mGluR–dependent plasticity in disorders where these pathways are disrupted, while minimizing off‐target effects arising from the widespread distribution and pleiotropic actions of mGluR subtypes.

## 8. mGluR–Dependent Bidirectional Synaptic Plasticity: LTP and LTD

The bidirectional regulation of synaptic strength through LTP and LTD represents a fundamental property of mGluR signaling, yet the underlying mechanisms are highly dependent on receptor subtype, synaptic localization, and intracellular signaling context. In contrast to the preceding sections on classical ionotropic plasticity, mGluR–dependent plasticity involves both presynaptic and postsynaptic processes, mediated by distinct receptor subtypes (e.g., mGluR1, mGluR5, and mGluR8) that engage diverse intracellular cascades including ERK/MAPK, PI3K–Akt–mTOR, p38–MK2, and protein synthesis–dependent pathways. Importantly, these mechanisms do not operate uniformly but instead exhibit region‐specific and activity‐dependent variability, resulting in differential expression of LTP or LTD across hippocampal and cortical circuits and in disease models. To provide a structured and mechanistic overview, the key experimental studies discussed in Section 8 are summarized in Table 5, with explicit emphasis on mGluR subtype specificity, synaptic locus of expression (pre‐ versus postsynaptic), and the associated intracellular signaling pathways. This framework facilitates a clearer understanding of how mGluRs contribute to bidirectional synaptic plasticity under both physiological and pathological conditions.

**Table 5:** mGluR subtype‐specific mechanisms underlying LTP and LTD.

Study	Region	mGluR	Plasticity	Locus	Mechanism	Key finding
[55]	Hippocampus (CA1, Schaffer collateral pathway)	mGluRs (likely Group I)	LTP (also depotentiation context)	Postsynaptic	Activation of a protein kinase–dependent molecular “switch”; can be reset by low‐frequency stimulation	mGluRs are required only once to set a persistent switch enabling LTP; unlike NMDA receptors, they do not need repeated activation
[56]	Hippocampus (CA3→CA1, also CA3→CA3)	Type I mGluRs (mGluR1/5)	LTP (NMDAR–independent)	Postsynaptic	Requires protein synthesis (FMRP–dependent) and Arc signaling; induced via VGCC activation	mGluR activation alone can induce LTP independent of NMDA receptors, forming a parallel plasticity system
[57]	Hippocampus (CA1)	Group I mGluRs	LTD	Mainly postsynaptic (with presynaptic components)	ERK/MAPK pathway activation (MEK–dependent); protein synthesis–dependent	ERK activation is required for mGluR–LTD, whereas p38 MAPK is not involved
[58]	Hippocampus (CA1)	Group I mGluRs (mGluR1/5)	LTD	Postsynaptic (with possible presynaptic contribution)	Activation of PI3K–Akt–mTOR signaling → translation initiation	PI3K and mTOR activation are essential for mGluR–LTD; blocking them abolishes LTD
[59]	Hippocampus (CA3–CA1)	mGluR5 (Group I)	LTD (enhanced, transient)	Postsynaptic	p38 MAPK → MK2 cascade regulates Arc expression and AMPAR endocytosis	mGluR–LTD is transiently enhanced in the AD model via MK2 signaling; deleting MK2 normalizes LTD and rescues plasticity and learning deficits
[60]	Hippocampus (CA1, SC–CA1 synapses)	Group I mGluRs (priming context)	LTP (and metaplasticity)	Postsynaptic	Arc ubiquitination regulates AMPAR endocytosis and mGluR–dependent priming of LTP	Disrupted Arc degradation reduces LTP magnitude and abolishes mGluR priming of LTP, highlighting Arc’s role in metaplasticity
[61]	Hippocampus (CA1, SC–CA1 synapses)	Group I mGluRs (mGluR1/5)	LTD	Both presynaptic and postsynaptic (distinct forms)	Low DHPG (30 µM): NMDAR–dependent ↓ release probability (P(r)); high DHPG (100 µM): NMDAR–independent AMPAR internalization + spine shrinkage	mGluR–LTD exists in two mechanistically distinct forms (pre versus post) that converge on the same low P(r) synapses
[62]	Prefrontal cortex (mPFC, Layer V pyramidal neurons)	mGluR1	LTD (impaired after cocaine)	Postsynaptic	mGluR1–mTOR–dependent LTD; cocaine → excessive D1/PKA signaling + CP‐AMPAR accumulation	Cocaine abolishes mGluR1–LTD, leading to CP‐AMPAR insertion and abnormal LTP; restoring mGluR1 rescues plasticity
[63]	Hippocampus (CA3–CA1, SC pathway)	Group I mGluRs (mGluR1/5)	LTD	Switch: postsynaptic → presynaptic (in SPKO)	Synaptopodin regulates mGluR5 surface expression and Homer interaction; loss → protein synthesis‐independent, endocannabinoid‐mediated LTD	Synaptopodin acts as a molecular switch controlling the mGluR–LTD mechanism; without it, LTD shifts from postsynaptic protein synthesis to presynaptic eCB signaling
[64]	Hippocampus (perforant path–dentate gyrus)	mGluR8 (Group III)	LTP	Presynaptic	Gi/o‐coupled mGluR8 activation reduces glutamate release; modulates excitation–inhibition balance	mGluR8 agonist inhibits LTP in controls but enhances LTP in the ASD (VPA) model, suggesting context‐dependent regulation of synaptic plasticity

Early work in hippocampal CA1 provided foundational evidence that mGluR activation can serve as a permissive signal for LTP induction independent of classical NMDAR mechanisms. The study by Bortolotto et al. [55] investigated the role of mGluRs in the induction of LTP in the CA1 region of rat hippocampal slices, specifically within the SC–commissural pathway. Using the selective mGluR antagonist (+)MCPG, the authors demonstrated that mGluR activation is required for LTP induction but not for short‐term potentiation (STP), as MCPG consistently blocked LTP in the majority of naïve slices while sparing STP responses. Importantly, their experiments revealed that mGluR activation does not function as a repetitive trigger during each tetanic stimulation, in contrast to NMDARs, but instead initiates a persistent, input‐specific “conditioning” process that enables subsequent LTP induction even in the continued presence of MCPG. This conditioning effect could be mimicked pharmacologically using the mGluR agonist ACPD and was shown to depend on intracellular signaling mechanisms involving protein kinase activation, as it was prevented by the kinase inhibitor K‐252b. Furthermore, the conditioning state was reversible, as low‐frequency stimulation abolished it, restoring MCPG sensitivity and thereby preventing LTP induction, suggesting a dynamic regulation of this mechanism. The study also demonstrated that this mGluR–dependent process operates independently of NMDAR–mediated LTP induction, as synaptic activation under NMDAR blockade (AP5) still produced conditioning without inducing LTP. Collectively, these findings indicate that mGluRs contribute to LTP induction through a distinct intracellular signaling mechanism that establishes a long‐lasting and activity‐dependent modulatory state, rather than acting as a direct trigger for synaptic potentiation [55].

Building on the idea that mGluRs can support potentiation independently of NMDARs, more recent work has delineated a mechanistically distinct, translation‐dependent form of mGluR–LTP at hippocampal synapses. The study by Wang et al. [56] examined whether mGluRs can independently support LTP in the hippocampus, focusing on CA3→CA1 synapses under conditions of NMDAR blockade. Using high‐frequency stimulation protocols in the presence of the NMDAR antagonist APV, the authors demonstrated that this form of LTP, previously described as VGCC–dependent LTP (VGCC–LTP), requires activation of Group I mGluRs (mGluR1 and mGluR5), as coapplication of selective antagonists (MPEP and LY‐367385) abolished LTP induction, whereas antagonism of Type II mGluRs had no effect. Importantly, the study showed that mGluR–dependent LTP (mGluR–LTP) relies on intracellular mechanisms distinct from classical NMDAR–LTP, including a strict dependence on protein synthesis, as inhibition with anisomycin prevented LTP induction. Furthermore, the authors identified a critical role for activity‐regulated cytoskeleton–associated protein (Arc) signaling, demonstrating that mGluR–LTP is abolished in Arc KO models and is associated with increased Arc protein expression following tetanic stimulation. In addition, regulation by Fragile X mental retardation protein (FMRP) was observed, as disruption of FMRP altered the sensitivity of mGluR–LTP to translational inhibition. The study also reported that mGluR–LTP and NMDAR–LTP are mechanistically distinct but can interact within the same synaptic pathways, exhibiting nonadditive effects under certain stimulation paradigms. Collectively, these findings demonstrate that activation of Group I mGluRs can induce an NMDAR–independent form of LTP that is tightly coupled to translational control and Arc–dependent intracellular signaling, supporting the existence of a parallel mGluR–based system for bidirectional synaptic plasticity [56].

In contrast to these potentiation mechanisms, a large body of work has characterized Group I mGluR–dependent LTD in CA1, revealing selective engagement of distinct MAPK and translational pathways. The study by Gallagher et al. [57] investigated the intracellular signaling pathways underlying Group I mGluR1/5–dependent LTD in the CA1 region of the hippocampus, with a particular focus on the role of MAPK signaling. Using both pharmacological activation (DHPG) and synaptic stimulation paradigms, the authors demonstrated that mGluR–dependent LTD (mGluR–LTD) requires activation of the ERK pathway, as inhibition of the upstream kinase MEK (using U0126 or PD98059) significantly attenuated LTD, whereas NMDAR–dependent LTD remained unaffected. Importantly, the study shows that this form of LTD is mechanistically distinct from other MAPK–dependent pathways, as inhibition of p38 MAPK did not alter mGluR–LTD in CA1, despite its known role in LTD in other hippocampal subregions such as the dentate gyrus. Biochemical analyses further revealed that DHPG stimulation induces robust phosphorylation of ERK and its downstream effector RSK, without significant activation of p38 MAPK, indicating a selective engagement of the ERK signaling cascade. Additionally, ERK activation was shown to depend on Group I mGluR stimulation, as coapplication of mGluR1 and mGluR5 antagonists reduced ERK phosphorylation. These findings position ERK as a key intracellular mediator linking Group I mGluR activation to protein synthesis–dependent synaptic depression, highlighting pathway specificity within mGluR signaling mechanisms. Overall, the study provides direct evidence that mGluR–dependent LTD in CA1 is selectively mediated by ERK signaling rather than p38 MAPK, supporting the concept that distinct intracellular cascades underlie different forms of synaptic plasticity [57].

Complementary to ERK, the PI3K–Akt–mTOR pathway has emerged as another critical axis coupling Group I mGluRs to translation‐dependent LTD. The study by Hou and Klann [58] investigated the intracellular signaling mechanisms linking Group I mGluR1/5 activation to protein synthesis–dependent LTD in the CA1 region, with a specific focus on the PI3K–Akt–mTOR pathway. Using the Group I mGluR agonist DHPG, the authors demonstrated that mGluR activation induces rapid and transient phosphorylation of key signaling components, including PDK1, Akt, and mTOR, as shown in western blot analyses. Pharmacological inhibition experiments revealed that blocking PI3K activity with LY294002 or wortmannin prevented DHPG–induced phosphorylation of Akt and mTOR and abolished mGluR–LTD, indicating that PI3K activity is required for both signaling activation and synaptic depression. Importantly, inhibition of mTOR using rapamycin selectively blocked the maintenance phase of LTD without affecting baseline transmission or the acute response, supporting a role for mTOR in translation‐dependent plasticity. Subcellular analyses further showed that Akt and mTOR phosphorylation occur in both dendritic and somatic compartments, as evidenced by synaptoneurosome preparations and confocal imaging of CA1 pyramidal neurons. Additionally, both mGluR1 and mGluR5 contribute to pathway activation, as antagonists for each receptor partially reduced Akt/mTOR phosphorylation, while combined inhibition completely blocked signaling. Together, these findings demonstrate that the PI3K–Akt–mTOR cascade functions as a critical intracellular pathway coupling Group I mGluR activation to translation initiation mechanisms required for the expression of mGluR–dependent LTD in hippocampal CA1 neurons [58].

More recent studies have extended these mechanistic insights into disease‐relevant contexts, highlighting how dysregulation of mGluR–LTD signaling cascades can shift the balance between LTP and LTD and contribute to cognitive impairment. The study by Privitera et al. [59] examined the role of the p38 MAPK–MK2 signaling cascade in regulating Group I mGluR–dependent LTD and associated cognitive function in the APP/PS1 murine model of AD. The authors demonstrated that mGluR–LTD is significantly enhanced at 7 months of age, as evidenced by increased LTD magnitude at CA3–CA1 synapses, indicating a shift in synaptic balance toward depression. This enhancement was linked to overactivation of mGluR5 signaling and required MK2 activity, as genetic deletion of MK2 normalized LTD to wild‐type levels without altering basal synaptic transmission or presynaptic function. Mechanistically, the study showed that elevated expression of the immediate early gene Arc, at both mRNA and protein levels, correlates with enhanced LTD in APP/PS1 mice, while MK2 deletion significantly reduces Arc expression, suggesting that MK2 regulates Arc–dependent AMPAR trafficking processes underlying LTD. Behavioral analysis using the Barnes maze revealed that APP/PS1 mice fail to adopt efficient spatial search strategies despite completing the task, whereas MK2 deletion restores the use of spatial strategies, linking synaptic plasticity alterations to cognitive deficits. Additionally, the enhancement of mGluR–LTD is transient, as LTD returns to wild‐type levels by 13 months, accompanied by reduced mGluR5 and Arc protein expression. Collectively, these findings demonstrate that the p38–MK2–Arc signaling axis mediates early‐stage dysregulation of mGluR–LTD and contributes to synaptic and cognitive impairments in AD models [59].

A convergent role for Arc in regulating the balance between mGluR–dependent LTD and LTP is further supported by genetic manipulation of Arc degradation. The study by Haley et al. [60] investigated how impaired degradation of the immediate early gene Arc influences synaptic plasticity and metaplasticity in the hippocampal CA1 region using ArcKR knock‐in mice. The authors report that basal synaptic transmission and paired‐pulse facilitation at SC–CA1 synapses remain unchanged between ArcKR and wild‐type mice, indicating preserved baseline synaptic function. However, synaptically induced mGluR–dependent LTD, elicited via paired‐pulse low‐frequency stimulation, is significantly enhanced in ArcKR mice, consistent with elevated Arc accumulation due to reduced proteasomal degradation. In contrast, theta‐burst stimulation‐induced LTP is significantly reduced at later time points (2 h postinduction) in ArcKR mice, while STP remains unaffected, suggesting a selective impairment in LTP maintenance rather than induction. Furthermore, the study demonstrates that Group I mGluR–dependent priming of LTP is abolished in ArcKR mice, as DHPG preapplication enhances LTP in wild‐type slices but fails to do so in ArcKR slices, indicating disrupted metaplastic regulation of synaptic strengthening. Mechanistically, the authors suggest that prolonged Arc accumulation may promote AMPA receptor endocytosis, thereby limiting potentiation and favoring synaptic depression. Additionally, under environmental enrichment conditions, ArcKR mice exhibit a reduced LTP‐to‐STP ratio despite no change in overall LTP magnitude, reflecting instability in sustained potentiation. Collectively, these findings indicate that regulated Arc degradation is essential for maintaining the balance between LTP and LTD and for enabling mGluR–dependent metaplasticity in hippocampal circuits [60].

Beyond postsynaptic signaling molecules, mGluR–dependent LTD also exhibits heterogeneity at the level of synaptic locus and recruitment of pre‐ versus postsynaptic mechanisms, which can be differentially engaged by the same receptor family. The study by Sanderson et al. [61] examined the mechanisms underlying mGluR–dependent LTD in hippocampal CA1 synapses, demonstrating that distinct induction conditions selectively recruit presynaptic and postsynaptic forms of LTD. Using different concentrations of the Group I mGluR agonist DHPG, the authors show that low‐dose stimulation (30 µM) induces an NMDAR–dependent presynaptic LTD, evidenced by a significant increase in paired‐pulse facilitation and a reduction in mEPSC frequency without changes in amplitude, indicating decreased neurotransmitter release probability. In contrast, high‐dose DHPG (100 µM) induces an NMDAR–independent postsynaptic LTD, characterized by reduced mEPSC amplitude with no change in frequency and accompanied by a decrease in dendritic spine size, consistent with AMPA receptor internalization. Importantly, both forms of LTD are expressed at the same population of synapses, particularly those with low basal release probability, as supported by computational modeling showing that selective modulation of low P(r) synapses reproduces experimental changes in EPSCs and paired‐pulse facilitation. Pharmacological analyses further reveal differential receptor involvement, where presynaptic LTD requires both mGluR1 and mGluR5 activation, while postsynaptic LTD is predominantly mediated by mGluR1. Additionally, acute slice experiments confirm that only presynaptic LTD is associated with changes in paired‐pulse facilitation, reinforcing the distinct loci of expression. Collectively, this study highlights that mGluR–LTD is not a uniform process but comprises mechanistically distinct presynaptic and postsynaptic components that are differentially engaged depending on stimulation conditions, yet converge on a specific subset of hippocampal synapses [61].

The bidirectional impact of mGluR signaling on synaptic strength is also evident in pathological or drug‐exposed cortical circuits, where loss of mGluR–dependent LTD can permissively enable aberrant LTP via AMPAR remodeling. The study by Ruan and Yao [62] investigated how repeated cocaine exposure alters mGluR1–dependent synaptic plasticity in the medial prefrontal cortex (mPFC), revealing a mechanistic link between impaired LTD and AMPA receptor remodeling. The authors demonstrate that DHPG–induced LTD in Layer 5 pyramidal neurons is predominantly mediated by mGluR1 and requires mTOR signaling, while being expressed postsynaptically, as indicated by the absence of changes in paired‐pulse ratio. Following repeated in vivo cocaine administration, this mGluR1–LTD is completely abolished after short withdrawal (1 day), partially recovers after 2 days, and returns to baseline after 7 days, indicating a transient and cumulative impairment. Mechanistically, the loss of LTD is associated with excessive dopamine D1 receptor signaling, as pharmacological inhibition of D1 receptors or intracellular PKA restores LTD expression. Concomitant with LTD impairment, cocaine exposure induces the accumulation of Ca^2+^‐permeable AMPA receptors (CP‐AMPARs), evidenced by reduced rectification index and increased sensitivity to Naspm. Importantly, these receptors are functionally relevant, as they enable an abnormal extension of spike‐timing‐dependent LTP (t‐LTP), specifically at longer timing intervals, which is blocked by CP‐AMPAR inhibition. Furthermore, in vivo activation of mGluR1 using a positive allosteric modulator restores both LTD and normal AMPAR composition, indicating that mGluR1 signaling regulates CP‐AMPAR removal. Collectively, this study demonstrates that cocaine‐induced disruption of mGluR1–LTD leads to maladaptive synaptic strengthening through CP‐AMPAR accumulation, highlighting a critical interaction between dopaminergic signaling, receptor trafficking, and synaptic plasticity in the prefrontal cortex [62].

At hippocampal CA3–CA1 synapses, structural spine components can further modulate which mGluR subtype contributes to LTD and whether plasticity is expressed pre‐ or postsynaptically. The study by Wu et al. [63] demonstrates that synaptopodin (SP), a postsynaptic actin‐associated protein, is a critical regulator of mGluR–dependent synaptic plasticity by controlling receptor localization and the molecular mechanism of LTD, at hippocampal CA3–CA1 synapses. Loss of SP significantly weakens DHPG–induced mGluR–LTD, indicating a functional deficit in synaptic depression. Mechanistically, the impairment is specifically linked to mGluR5 rather than mGluR1, as mGluR5 activity becomes dispensable for LTD in SP KO (SPKO) mice despite unchanged total receptor expression. This functional loss is explained by a marked reduction in surface mGluR5 levels and disrupted interaction with the scaffolding protein Homer, which normally stabilizes receptor clustering and signaling at the postsynaptic membrane. In parallel, the canonical protein synthesis–dependent mechanism of mGluR–LTD is abolished in SPKO mice, as inhibition of translation fails to block LTD, indicating a shift away from postsynaptic translational control. Instead, LTD becomes dependent on eCB signaling, with CB1 receptor inhibition abolishing synaptic depression specifically in SPKO conditions, demonstrating a switch to a presynaptic mode of expression. Importantly, upstream signaling pathways such as ERK1/2 and mTOR remain largely intact, suggesting that the deficit arises from altered receptor localization and signaling coupling rather than global signaling failure. Collectively, this study identifies SP as a molecular switch that determines both the receptor subtype involvement and the locus of expression of mGluR–LTD, linking spine structural components to intracellular signaling and synaptic plasticity mechanisms [63].

While most of the above studies focus on Group I receptors, Group III mGluRs can also modulate LTP bidirectionally in a region‐ and state‐dependent manner. The study by Gholipour et al. [64] investigates the role of Group III mGluR mGlu8 in regulating hippocampal synaptic plasticity in a prenatal valproic acid (VPA)–induced rat model of autism, focusing on perforant path–dentate gyrus synapses. The authors report that VPA exposure significantly reduces LTP, as evidenced by decreased fEPSP slope and population spike amplitude following high‐frequency stimulation. Pharmacological activation of mGlu8 using the selective agonist (S)‐3,4‐DCPG produces bidirectional effects: it suppresses LTP in control animals while enhancing LTP in VPA–exposed rats, indicating context‐dependent modulation of synaptic plasticity. Mechanistically, mGlu8 receptors are predominantly localized presynaptically in the molecular layer of the dentate gyrus, where they function as Gi/o‐coupled autoreceptors that inhibit glutamate release and regulate synaptic transmission. The study further suggests that in the pathological condition, mGlu8 activation may facilitate LTP indirectly by reducing GABAergic inhibition, which otherwise suppresses NMDAR activation required for LTP induction. Supporting this, the schematic and electrophysiological recordings illustrate how changes in EPSP slope and population spike amplitude are used to quantify synaptic strengthening at perforant path–dentate gyrus synapses. Additionally, behavioral deficits in social novelty preference observed in VPA–exposed rats are reversed by intradentate gyrus administration of DCPG, linking synaptic plasticity changes with functional outcomes. Overall, this study highlights a presynaptic regulatory mechanism of mGlu8 that modulates both glutamatergic and GABAergic balance, thereby influencing LTP dynamics and behavioral phenotypes in an autism model [64].

Although the studies synthesized in Section 8 substantially advance understanding of mGluR–dependent bidirectional plasticity, several limitations constrain how far these findings can be generalized across brain regions and behavioral states. Most experiments rely on acute slice preparations, supraphysiological agonist concentrations (e.g., DHPG), or strong stimulation paradigms, which may selectively engage particular mGluR subtypes or signaling axes (ERK/MAPK, PI3K–Akt–mTOR, p38–MK2–Arc, and eCBs) in ways that only partially reflect in vivo synaptic activity patterns. In addition, many studies focus on a single cellular locus or molecular node (such as Arc, SP, or CP‐AMPARs) and a single synapse type (e.g., CA3–CA1, PP–DG, mPFC Layer 5), making it difficult to determine whether the identified mechanisms represent general principles of mGluR–dependent LTP/LTD or are circuit‐ and state‐specific specializations. Genetic or pharmacological manipulations used to probe mGluR1/5 and mGlu8 signaling also often lack cell‐type or compartmental resolution, leaving open questions about how astrocytic receptors, interneuron networks, and neuromodulatory inputs integrate with these pathways to shape the net balance between potentiation and depression. Future work will benefit from combining cell type–specific and subcellularly targeted tools (e.g., conditional KOs, opto/chemogenetic control of defined mGluR subtypes, and nanoscale imaging of receptor nanodomains) with in vivo recording during behavior to map how mGluR–dependent mechanisms operate under naturalistic patterns of activity and plasticity demands. A key outlook is to translate the mechanistic insights from these studies, such as Arc‐ and MK2–dependent tuning of AMPAR trafficking, SP–Homer control of mGluR5 localization, and mGlu8–mediated presynaptic gating of excitation–inhibition balance, into circuit‐level models that predict when and where mGluR signaling will favor LTP versus LTD, and how these decision points can be selectively targeted in neuropsychiatric and NDDs without disrupting physiological plasticity.

## 9. Cortical Region‐Specific mGluR–Dependent LTD and Metaplasticity

**Figure 2:**
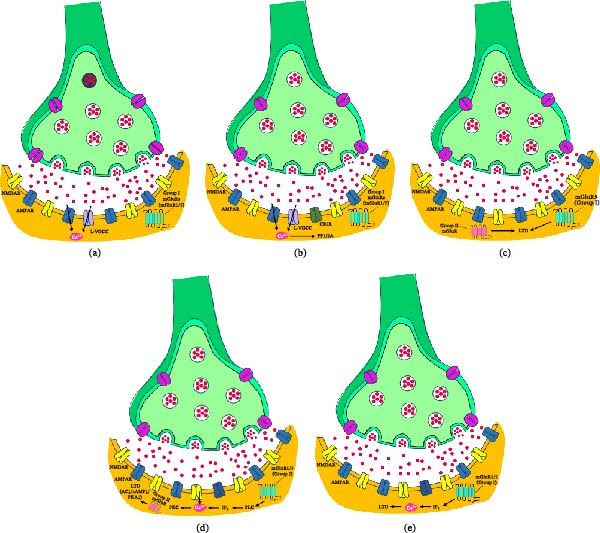
Cortical circuits and receptor mechanisms underlying mGluR–dependent LTD. (a) Anterior cingulate cortex (ACC): LTD is expressed via Group I mGluR1 in cooperation with L‑type voltage‑gated calcium channels (L‑VGCCs) and a partial contribution of NMDA receptors (NMDARs). (b) Insular cortex (IC): LTD requires mGluR5, NMDARs, L‑VGCCs, cannabinoid CB1 receptors, and the protein phosphatases PP1/2A. (c) Perirhinal cortex: LTD involves Group II mGluRs together with mGluR5 (Group I) and NMDAR–mediated synaptic activation. (d) Prefrontal cortex: LTD is mediated by Group II mGluRs through modulation of AC–cAMP–PKA signaling, while Group I mGluR1/5 engages a parallel PLC–IP_3_–Ca^2+^–PKC pathway at the postsynaptic site reflecting the canonical coupling of Group I mGluRs to PLC–IP_3_ signaling, with Group II recruiting PLC specifically in medial prefrontal cortex as reported by [67]. (e) Visual cortex: LTD is supported by Group II mGluRs, with Group I mGluR5 contributing via PLC–IP_3_–dependent postsynaptic signaling.

Cortical mGluR–dependent synaptic plasticity exhibits substantial regional diversity, reflecting differences in receptor subtype distribution, subcellular localization, and intracellular signaling mechanisms across distinct cortical areas. While early studies established that mGluR activation can drive LTD through G‐protein–coupled pathways independent of ionotropic receptors, subsequent work has revealed that these mechanisms are highly context‐dependent, varying across sensory, associative, and limbic cortices. In particular, the relative contributions of mGluR subtypes (for example, mGluR1, mGluR5, and Group II receptors), their localization at pre‐ versus postsynaptic compartments, and their coupling to signaling cascades such as IP_3_–Ca^2+^, MAPK/ERK, and PKC pathways collectively determine the direction and persistence of synaptic plasticity. Moreover, these processes are further modulated under pathological conditions, where region‐specific alterations in mGluR signaling contribute to disrupted plasticity and maladaptive circuit function. To systematically integrate these findings, key studies examining cortical region‐specific mGluR plasticity are summarized in Table 6, highlighting the relationships between receptor subtype, cellular localization, intracellular signaling pathways, and plasticity outcomes. These region‐specific mechanisms are schematized in Figure 2, which illustrates the cortical circuits and receptor signaling cascades underlying mGluR–dependent LTD across ACC, insular cortex (IC), perirhinal, prefrontal, and visual cortices.

**Table 6:** Cortical region‐specific mGluR plasticity.

Study	Cortical region	mGluR subtypes	Subcellular localization	Intracellular signaling	Plasticity type
[65]	Visual cortex (Layers II–IV)	InsP_3_–linked mGluRs (likely Group I: mGluR1/5)	Postsynaptic (demonstrated)	G‐protein–mediated signaling → InsP_3_ production → Ca^2+^ release from intracellular stores	Homosynaptic LTD (NMDA–independent)
[66]	Perirhinal cortex (Layers I–III)	Groups I–III mGluRs (not subtype‐specific)	Not determined	Pharmacological activation of mGluRs produces synaptic depression; signaling mechanisms consistent with canonical mGluR pathways (not directly tested)	Long‐lasting depression (Groups I and II); short‐term depression (Group III)
[67]	Medial prefrontal cortex (Layers I and II → V pyramidal neurons)	Group II mGluRs	Postsynaptic (functionally demonstrated)	Group II mGluR activation → PLC/IP_3_ signaling → intracellular Ca^2+^ release → PKC activation; requires ongoing NMDA receptor activity	Homosynaptic LTD
[68]	Prefrontal cortex (Layer I–II → V pyramidal neurons)	Dopamine receptors (D1/D2) + Groups I/II mGluRs	Predominantly postsynaptic (supported)	(i) Dopamine + mGluR coactivation → MAPK (ERK) signaling; (ii) Group II mGluR activation alone → Ca^2+^‐dependent, MAPK–independent pathway	Multiple LTD forms: dopamine‐dependent LTD and mGluR–induced LTD
[69]	Insular cortex (Layers II and III, V and VI; adult mouse)	mGluR5 (Group I)	Not determined (network‐level recording)	NMDA receptor + mGluR5 + L‐type VGCC activation; endocannabinoid signaling; protein phosphatases (PP1/2A); independent of PKC, PKA, CaMKII	Two LTD forms: NMDA–dependent (LFS–induced) and mGluR–dependent (DHPG–induced)
[70]	Insular cortex (Layers II and III, V and VI)	Group I mGluRs (DHPG–sensitive)	Not determined	PKC–dependent metaplastic signaling restores LTD after amputation; PKA and CaMKII are not required	LTD–related metaplasticity: loss of LFS–LTD, intact DHPG–LTD, rescue by mGluR priming
[71]	Anterior cingulate cortex (Layers II/III pyramidal neurons)	mGluRs (MCPG–sensitive; subtype not specified)	Not determined	NMDA–independent LTD requires L‐type Ca^2+^ channels and mGluR activation; independent of NMDA receptors	Homosynaptic LTD (LFS–induced)
[72]	Anterior cingulate cortex (Layers II/III, V, VI)	mGluR1 (Group I)	Not determined (network‐level recording)	Requires mGluR1 and L‐type VGCC; NMDA receptors partially contribute; PKC–dependent metaplastic rescue of LTD	LFS–induced LTD; impaired after injury and restored by mGluR1 priming
[73]	Insular cortex (Layers II and III, V and VI)	mGluR1 (Group I; role of mGluR5 not established)	Likely postsynaptic (supported)	NMDA receptor (GluN2A/GluN2B) activation; L‐type VGCC; protein synthesis–dependent; recruitment of Ca^2+^‐permeable AMPARs	Late‐phase LTP (L‐LTP)
[74]	Insular cortex (Layers II/III)	NMDAR–dependent (mGluRs not tested)	Synaptic (demonstrated)	AC1 → cAMP → PKA → Src kinases → GluN2B phosphorylation → increased synaptic NMDARs	Injury‐induced potentiation; LTP–like enhancement

A foundational work by Kato [65] suggests that in the rat visual cortex (Layers II–IV), homosynaptic LTD can be induced independently of ionotropic glutamate receptor activation and postsynaptic depolarization, instead requiring activation of postsynaptic mGluRs. LTD is elicited by presynaptic tetanization even in the presence of NMDAR and AMPA/kainate receptor blockers (APV and CNQX), indicating that classical ionotropic mechanisms are not necessary for its induction. At the subcellular level, the mechanism is postsynaptic, as intracellular interventions selectively disrupt LTD: blockade of G‐protein signaling using GDP‐β‐S, inhibition of inositol 1,4,5‐trisphosphate (IP_3_) receptors with heparin, and intracellular calcium chelation (EGTA or BAPTA) all prevent LTD without affecting baseline synaptic transmission or the ability to induce LTP. These findings indicate that LTD induction depends on a G‐protein–coupled signaling cascade leading to IP_3_–mediated calcium release from intracellular stores rather than calcium influx through membrane channels. Pharmacological activation further supports this mechanism, as application of the mGluR agonist quisqualate induces LTD in the absence of tetanic stimulation, whereas the agonist trans‐ACPD, which preferentially activates cAMP–linked mGluRs, fails to do so, indicating selective involvement of IP_3_‐coupled mGluR subtypes, likely within Group I (mGluR1 and/or mGluR5). Importantly, LTD and LTP can be induced in the same neuron but rely on distinct calcium sources, with LTD depending on intracellular Ca^2+^ release via mGluR signaling and LTP requiring NMDAR activation. Collectively, this study establishes a postsynaptic, G‐protein‐, and IP_3_–dependent mGluR signaling pathway as the primary mechanism underlying homosynaptic LTD in the visual cortex [65] and provides a canonical example of Group I mGluR–driven LTD in a sensory cortical area.

Moving from primary sensory to associative cortex, McCaffery et al. [66] demonstrated that the rat perirhinal cortex illustrates how the engagement of multiple mGluR groups broadens the repertoire of depression mechanisms. In this region, pharmacological activation of mGluRs induces both transient and long‐lasting synaptic depression in superficial cortical layers (Layers I–III), where extracellular field potentials are recorded in response to stimulation of superficial and intermediate inputs originating from adjacent entorhinal and temporal cortices. Functional evidence indicates the presence of all three mGluR groups (I–III), as activation with selective agonists produces distinct forms of synaptic modulation: the broad‐spectrum agonist (1S,3R)‐ACPD, the Group I agonist DHPG, and the Group II agonist DCG‐IV each induce an initial strong depression followed by a sustained LTD that persists after washout, whereas activation of Group III receptors with L‐AP4 produces only a reversible, short‐term depression. The LTD induced by Groups I and II receptor activation is consistently observed across both superficial and intermediate pathways, indicating a distributed network effect rather than pathway‐specific plasticity. Although precise subtype contributions (for example, mGluR1 versus mGluR5 or mGluR2 versus mGluR3) cannot be distinguished pharmacologically in this study, Group I receptors are linked to PLC and IP_3_ signaling, while Groups II/III receptors are associated with inhibition of cyclic AMP pathways, reflecting distinct intracellular signaling mechanisms. Mechanistically, the initial depression induced by Groups II and III activation is suggested to involve presynaptic inhibition of neurotransmitter release, whereas Group I‐mediated effects may involve postsynaptic depolarization through modulation of potassium conductances, although the exact subcellular localization within the perirhinal cortex remains unresolved. Notably, the requirement for relatively high concentrations of L‐AP4 to induce depression suggests a predominant contribution of low‐affinity mGluR7 rather than mGluR4 in this cortical region. Collectively, this study establishes that activation of multiple mGluR groups can differentially regulate synaptic transmission and induce long‐lasting depression in the perirhinal cortex, supporting a potential role for mGluRs in experience‐dependent reductions in neuronal responsiveness associated with recognition memory and extending the concept of mGluR–dependent LTD from strictly postsynaptic Group I pathways to mixed pre‐ and postsynaptic mechanisms [66].

Within the mPFC (prelimbic area), these principles are further refined by Otani et al. [67] demonstrated that they dissect the contribution of specific Group II receptors and their interaction with other neuromodulatory systems. In this region, activation of Group II mGluRs, specifically via the agonist DCG‐IV, induces LTD at Layer I and II to Layer V pyramidal neuron synapses through a postsynaptic mechanism. Anatomically and functionally, these receptors are localized postsynaptically on pyramidal neurons, as evidenced by intracellular manipulations and Ca^2+^ imaging, which reveal that LTD induction depends on postsynaptic signaling rather than presynaptic modulation. Pharmacological experiments confirm subtype specificity, as LTD is blocked by the Group II mGluR antagonist MSOPPE and reproduced by another Group II agonist (L‐CCG‐I), while Group I mGluR blockade (AIDA) does not affect LTD induction. Mechanistically, LTD requires synaptic activation of NMDARs, although DCG‐IV does not potentiate NMDAR–mediated currents, indicating that basal NMDAR activity contributes permissively to LTD induction. At the intracellular level, Group II mGluR activation engages a signaling cascade involving PLC and phospholipase D (PLD), leading to IP_3_–mediated Ca^2+^ release from intracellular stores, as demonstrated by blockade with U‐73122, PCCG‐13, and intracellular heparin. These Ca^2+^ elevations are directly observed in Layer V pyramidal neurons using fluorescence imaging and are tightly coupled to LTD expression. Downstream, PKC activation is essential, as both pharmacological inhibition (RO318220) and postsynaptic peptide inhibition suppress LTD, while PKA also contributes to LTD expression. Importantly, interruption of synaptic stimulation during agonist application prevents LTD, indicating that this form of plasticity is homosynaptic and requires coincident synaptic activity. Collectively, this study establishes a postsynaptic Group II mGluR–dependent LTD in the prefrontal cortex driven by PLC/PLD–IP_3_–Ca^2+^ signaling and kinase activation, highlighting that, in contrast to the visual cortex, postsynaptic Group II rather than Group I receptors can dominate LTD in an associative cortical area [67]. Thus, although PLC–IP_3_ signaling is typically associated with Group I mGluRs, in mPFC Group II mGluRs can also couple to PLC/PLD–IP_3_–Ca^2+^ cascades.

A second study by Otani et al. [68] in the same prelimbic region demonstrates that these Group II‐dependent mechanisms do not act in isolation but instead converge with Group I mGluR and dopaminergic signaling. In this study, LTD at Layer I and II to Layer V pyramidal neuron synapses requires coordinated activation of dopamine receptors and both Groups I and II mGluRs, reflecting a postsynaptic convergence of signaling pathways. Group I mGluRs (particularly mGluR5) are localized to postsynaptic dendritic spines and shafts of pyramidal neurons, whereas mGluR1 is enriched in nonpyramidal neurons, and Group II mGluRs are present at both pre‐ and postsynaptic sites, supporting their functional involvement in synaptic modulation. Tetanic stimulation (50 Hz) alone does not induce lasting plasticity, and dopamine application alone produces only transient depression; however, when tetanic stimulation is paired with dopamine, robust LTD is induced, indicating a facilitatory role of dopaminergic signaling. This LTD is abolished by antagonists of Group I (AIDA) and Group II (MSOPPE) mGluRs, as well as by the broad‐spectrum antagonist MCPG, demonstrating that synaptic activation of both mGluR groups is required. Pharmacological activation further reveals that co‐activation of Groups I and II mGluRs with 1S,3R‐ACPD facilitates LTD induction, whereas selective activation of Group I mGluRs alone (DHPG) is insufficient, while activation of Group II mGluRs (DCG‐IV) can independently induce a postsynaptic Ca^2+^‐dependent LTD. Importantly, coapplication of dopamine with mGluR agonists is sufficient to induce LTD even in the absence of synaptic stimulation, indicating that receptor coactivation alone can engage the necessary intracellular machinery. At the signaling level, LTD induction requires convergent activation of MAPKs/ERKs, as biochemical analyses show that dopamine receptors (D1 and D2) and both Groups I and II mGluRs contribute to MAPK activation, and pharmacological inhibition of MAPK kinase (PD98059) or intracellular blockade of MAPK signaling prevents LTD. Collectively, this study establishes a postsynaptic, MAPK–dependent mechanism in which dopamine receptors and multiple mGluR subtypes cooperatively regulate LTD in the prefrontal cortex, thereby illustrating how cortical mGluR–dependent plasticity can be gated by neuromodulatory systems [68].

The IC provides a further example of how distinct mGluR subtypes and their interaction with NMDARs and eCB signaling shape LTD within a multimodal cortical area. In adult mouse IC slices, low‐frequency stimulation (1 Hz) induces LTD of glutamate receptor–mediated excitatory synaptic transmission across both superficial (Layers II/III) and deep layers (V/VI), as shown using a 64‐channel multielectrode array that captures the spatial distribution of network activity. Synaptic responses are primarily mediated by postsynaptic AMPA/kainate receptors, with stimulation delivered to deep layers and recordings obtained from superficial pyramidal neurons, indicating a defined laminar organization of excitatory inputs. Mechanistically, LTD induction requires the coordinated activation of NMDARs, mGluR5, and L‐VGCCs, as pharmacological blockade with AP5, MPEP, or nimodipine abolishes LTD, whereas inhibition of mGluR1 with CPCCOEt has no effect, demonstrating subtype‐specific involvement of mGluR5 within Group I mGluRs. At the intracellular level, LTD depends on protein phosphatases PP1/PP2A and eCB signaling via CB1 receptors, as inhibition of either pathway prevents LTD induction, while multiple kinase pathways, including PKC, PKA, CaMKII, and PKMζ are not required. In addition to electrically induced LTD, bath application of the Group I mGluR agonist DHPG produces a distinct, NMDAR–independent form of LTD that does not occlude low‐frequency stimulation‐induced LTD, indicating the coexistence of mechanistically separable LTD forms within the same IC synapses. Spatial analysis further reveals that LTD is not uniformly distributed across all activated channels but is more prominent near stimulation sites, highlighting network‐level heterogeneity in synaptic depression. Collectively, this study defines both NMDAR–dependent and mGluR5–dependent LTD in the IC with distinct intracellular signaling requirements and spatial characteristics, underscoring that even within a single cortical region, multiple mGluR–dependent LTD forms can coexist [69].

A subsequent study by Liu and Zhuo [70] in the same preparation demonstrates that these IC LTD mechanisms are differentially affected by peripheral injury, revealing a metaplastic role for Group I mGluRs. Under physiological conditions, 1 Hz stimulation induces NMDAR–dependent LTD across both superficial (Layers II/III) and deep (Layers V/VI) cortical layers, as recorded with a multielectrode array. Following peripheral injury induced by tail amputation, there is a complete loss of low‐frequency stimulation‐evoked LTD in both layers, accompanied by a significant reduction in the proportion of LTD‐expressing synaptic sites, while baseline synaptic transmission remains intact. In contrast, a chemically induced form of LTD triggered by the Group I mGluR agonist DHPG remains unaffected, indicating the coexistence of mechanistically distinct LTD forms with differential vulnerability to injury. The preserved DHPG–LTD suggests that mGluR–dependent signaling pathways remain functionally intact despite the loss of activity‐dependent LTD. Notably, priming the IC with a low concentration of DHPG restores the ability to induce low‐frequency stimulation‐dependent LTD, demonstrating a form of mGluR–mediated metaplasticity that rescues synaptic depression. Pharmacological analysis reveals that this rescue mechanism specifically requires PKC activation, as inhibition with chelerythrine abolishes LTD recovery, whereas inhibition of CaMKII or PKA has no effect. Additionally, peripheral injury enhances excitatory synaptic transmission, as indicated by a leftward shift in input–output curves, suggesting increased postsynaptic excitability that may contribute to LTD impairment. Collectively, this study identifies a dissociation between NMDAR–dependent and mGluR–dependent LTD in the IC and establishes a PKC–dependent, Group I mGluR–mediated metaplastic mechanism that restores LTD following injury, thereby linking region‐specific mGluR function to injury‐induced plasticity changes [70].

Similar interactions between mGluR signaling, intrinsic excitability, and injury are observed in the ACC, a key region for affective and pain‐related processing. In the ACC of adult rats, LTD is expressed primarily in Layers II/III pyramidal neurons following stimulation of Layer V inputs, indicating a laminar organization of excitatory synaptic transmission mediated by AMPA/kainate receptors. Low‐frequency stimulation (1 Hz, 15 min) induces a long‐lasting, input‐specific LTD that is independent of NMDAR activation but critically requires both L‐VGCCs and mGluRs, as demonstrated by complete blockade with nimodipine and MCPG, respectively, while AP‐5 has no effect. Although the specific mGluR subtype is not pharmacologically distinguished in this study, the requirement for mGluRs indicates a non‐ionotropic mechanism underlying LTD induction. At the cellular level, LTD is expressed in a pathway‐specific manner, suggesting synapse‐specific plasticity within ACC circuits. Following peripheral injury (hindpaw digit amputation), there is a rapid and bilateral increase in immediate early gene expression (c‐Fos, NGFI‐A, and pCREB) in ACC neurons, indicating enhanced neuronal activity and intracellular transcriptional signaling. Concomitantly, LTD is abolished as early as 45 min postamputation and remains impaired for at least 2 weeks, without affecting baseline synaptic transmission, suggesting a selective disruption of depression mechanisms rather than general synaptic failure. This loss of LTD is region‐specific, as similar stimulation protocols still induce LTD in the parietal cortex, and is proposed to be associated with increased postsynaptic excitability and activity‐dependent plastic changes in ACC neurons following injury. Collectively, this study establishes an NMDAR–independent, mGluR‐ and L‐VGCC–dependent LTD in the ACC and demonstrates its selective impairment following peripheral amputation, paralleling the IC findings but with distinct receptor requirements [71].

Subsequent work by Kang et al. [72] using multielectrode recordings in the mouse ACC has refined these observations by directly identifying the responsible mGluR subtype. In adult mice, low‐frequency stimulation applied to deep Layer V induces LTD across a local cortical network, particularly in Layers II/III and V/VI, as revealed using a 64‐channel multielectrode array system. Excitatory synaptic transmission in this circuit is mediated by postsynaptic AMPA/kainate receptors, while LTD induction requires activation of mGluRs, with additional contributions from NMDARs and L‐VGCCs. Pharmacological analysis specifies that this form of LTD is critically dependent on mGluR1, as it is blocked by the mGluR1 antagonist LY367385 and by the broad mGluR antagonist MCPG, whereas the mGluR5 antagonist MPEP does not affect LTD, indicating subtype‐specific involvement of Group I mGluRs. Peripheral injury (distal tail amputation) results in a persistent impairment of ACC LTD without altering baseline synaptic transmission or mGluR1 expression levels, suggesting a functional rather than expression‐level deficit. Importantly, LTD can be restored through a metaplastic mechanism involving selective activation of mGluR1 using coapplication of DHPG and MPEP, which primes the system and enables subsequent low‐frequency stimulation to induce LTD; this rescue process requires PKC activity but not CaMKII or PKA. Collectively, this study defines a network‐level, mGluR1–dependent LTD in the ACC with specific pharmacological and intracellular signaling requirements and demonstrates its disruption and recoverability following peripheral injury, providing a clear subtype‐specific counterpart to the predominantly mGluR5–dependent LTD described in the IC [72].

Within the same insular cortical network, mGluR signaling is not restricted to LTD but also contributes to LTP, further illustrating the bidirectional role of these receptors in cortical plasticity. In adult mouse IC slices, theta‐burst stimulation applied to deep layers (V–VI) induces a long‐lasting, protein synthesis–dependent late‐phase LTP (L‐LTP) that is expressed across both superficial (II/III) and deep cortical layers, as revealed using a 64‐channel multielectrode array. L‐LTP induction requires coordinated activation of multiple receptor systems, including NMDARs containing both GluN2A and GluN2B subunits, L‐VGCCs, and mGluR1, as pharmacological blockade with AP5, NVP‐AAM077, Ro 25‐6981, nimodipine, or the mGluR1 antagonist CPCCOEt significantly impairs LTP induction, whereas inhibition of mGluR5 (MPEP) or Groups II/III mGluRs (EGLU and MSOP) has no effect, indicating subtype‐specific involvement of mGluR1. At the network level, L‐LTP is not uniformly distributed but is spatially restricted to subsets of activated synaptic sites within approximately 450 μm of the stimulation locus, with comparable induction probability in superficial and deep layers, indicating layer‐independent but spatially constrained plasticity. Mechanistically, induction depends on new protein synthesis, as anisomycin abolishes L‐LTP and markedly reduces the spatial extent of potentiated channels. Expression of L‐LTP is postsynaptic, as indicated by the lack of change in paired‐pulse facilitation and the requirement for recruitment of calcium‐permeable AMPA receptors. Additionally, although NMDAR activation is critical, partial persistence of LTP under AP5 suggests the presence of NMDAR–independent components. Collectively, this study defines a postsynaptic, mGluR1–dependent and protein synthesis–dependent form of L‐LTP in the IC with distinct receptor requirements and spatial organization, indicating that, within a single cortical region, mGluR1 can support both potentiation and depression depending on the induction protocol [73].

Finally, although not directly manipulating mGluRs, work on injury‐induced plasticity in the IC further constrains the context in which mGluR–dependent mechanisms operate by characterizing parallel changes in NMDAR function. In the adult mouse IC, peripheral nerve injury induces a long‐term enhancement of synaptic plasticity characterized by an increase in synaptic NMDARs, particularly within the PSD of pyramidal neurons in layers II/III, without changes in extrasynaptic receptor pools. Biochemical fractionation shows that GluN1, GluN2A, and GluN2B subunits are selectively upregulated in the PSD fraction beginning at day 7 postinjury and persisting for at least 28 days, indicating activity‐dependent redistribution rather than global protein upregulation. Electrophysiological recordings reveal enhanced NMDAR–mediated currents in pyramidal neurons, reflected by a steeper input–output relationship without alteration in current–voltage properties, suggesting increased synaptic receptor function rather than altered channel kinetics. Functionally, LTP induced by pairing protocols is NMDAR–dependent and partially mediated by both GluN2A– and GluN2B–containing receptors, but is occluded following nerve injury, indicating that injury‐induced plasticity shares mechanisms with activity‐dependent LTP. At the intracellular level, the increase in synaptic NMDARs is driven by a calcium‐sensitive signaling cascade involving adenylyl cyclase subtype 1 (AC1), PKA, and Src family kinases, which converge to enhance phosphorylation of the GluN2B subunit at Tyr1472, a modification that reduces receptor endocytosis and promotes synaptic retention. Pharmacological and genetic manipulations confirm that AC1 is required for this process and that inhibition of PKA or Src family kinases prevents both phosphorylation and synaptic accumulation of NMDARs. Imaging data further show increased surface localization of GluN2B–containing receptors without changes in total receptor levels, supporting a trafficking‐based mechanism. Behaviorally, intrainsular administration of NMDAR antagonists reduces mechanical allodynia, linking these synaptic changes to neuropathic pain processing. Collectively, this study establishes a postsynaptic, subtype‐specific (GluN2B–enriched) and signaling‐driven enhancement of synaptic NMDAR function in the IC, mediated by AC1–PKA–Src family kinase pathways and associated with persistent pain‐related plasticity, thereby providing a complementary ionotropic context in which mGluR–dependent cortical plasticity is engaged during chronic pain states [74].

Taken together, the studies summarized in this section indicate that cortical mGluR–dependent plasticity is highly region‐ and subtype‐specific, with Group I (mGluR1/mGluR5) and Group II receptors differentially contributing to LTD and LTP across sensory (visual and perirhinal), associative (prefrontal), and limbic/pain‑related (ACC and IC) cortices (Figure 2). A recurring conclusion is that postsynaptic IP_3_–Ca^2+^ signaling and downstream kinases or phosphatases (for example, PKC, MAPKs, and PP1/PP2A) are central for mGluR–dependent LTD and L‐LTP, while metaplastic priming via Group I mGluRs (particularly mGluR1) can restore lost LTD after injury in ACC and IC, linking receptor subtype, intracellular cascades, and injury‐induced plasticity in a coherent framework. At the same time, these data sets have important limitations: most experiments are performed in acute rodent slices or under anesthetized conditions, focus predominantly on excitatory pyramidal neurons and Groups I/II mGluRs, and often rely on pharmacological tools that cannot fully resolve individual receptor subtypes or distinguish pre‐ from postsynaptic contributions, which constrains the generalization of these mechanisms to behaving animals and to other cortical cell types such as inhibitory interneurons and astrocytes. Furthermore, the available studies only sample a subset of cortical regions and behavioral states (with a strong emphasis on pain models in ACC and IC), leaving open how similar or distinct mGluR–dependent plasticity rules are in other higher‐order areas (for example, motor and prefrontal subregions) and in disease‐relevant contexts such as neurodegeneration, psychiatric disorders, and aging. Future work will, therefore, need to combine cell type–specific genetic manipulation of defined mGluR subunits with in vivo recordings and behavior, extend analyses to inhibitory and glial networks, and systematically compare physiological and pathological conditions to determine when mGluR–dependent LTD/LTP and metaplasticity are adaptive versus maladaptive. Such studies may ultimately clarify whether targeting specific cortical mGluR subtypes or their downstream signaling nodes can provide circuit‐selective therapeutic strategies, for example, restoring ACC/IC LTD in chronic pain or normalizing dysregulated mGluR signaling in neurodegenerative and psychiatric disorders, while minimizing off‐target effects on normal cortical processing.

## 10. Emerging Roles of mGluRs in NDDs

**Figure 3:**
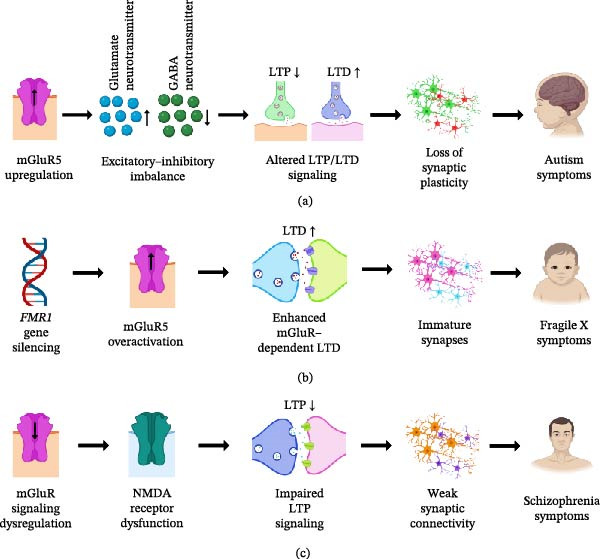
Mechanistic links between mGluR5 dysregulation and neurodevelopmental disorders. (a) Upregulation of mGluR5 signaling disrupts the excitatory–inhibitory balance (increased glutamatergic and decreased GABAergic transmission), leading to altered LTP/LTD, loss of synaptic plasticity, and manifestation of autism‐related symptoms. (b) FMR1 gene silencing in Fragile X syndrome causes mGluR5 overactivation, which enhances mGluR–dependent LTD, promotes immature spine/synapse morphology, and results in Fragile X–associated behavioral symptoms. (c) mGluR signaling dysregulation can induce NMDA receptor dysfunction, impairing LTP and weakening synaptic connectivity, ultimately contributing to schizophrenia‐like symptoms.

**Figure 4:**
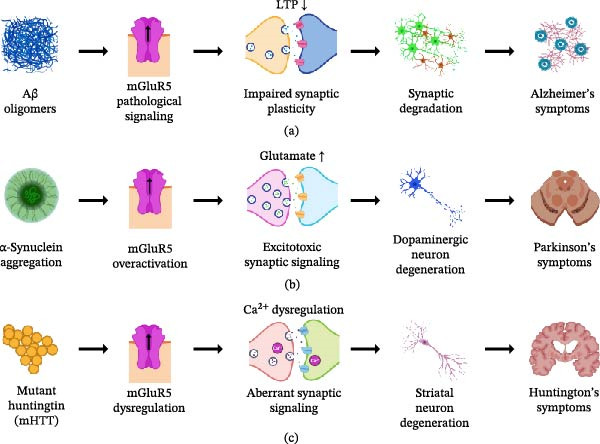
Pathological mGluR5 signaling in major neurodegenerative disorders. (a) In Alzheimer’s disease, Aβ oligomers drive pathological mGluR5 activation, leading to impaired LTP, progressive synaptic degradation, and the emergence of Alzheimer’s‐like cognitive symptoms. (b) In PD, α‐synuclein aggregation promotes mGluR5 overactivation, causing excitotoxic glutamatergic synaptic signaling, dopaminergic neuron degeneration, and Parkinsonian motor symptoms. (c) In Huntington’s disease, mutant huntingtin (mHTT) induces mGluR5 dysregulation and Ca^2+^‐dependent aberrant synaptic signaling, resulting in striatal neuron loss and characteristic Huntington’s symptoms.

mGluRs have emerged as a unifying yet highly heterogeneous mechanism across neurodegenerative and neuropsychiatric disorders, where subtype‐specific alterations in receptor expression, subcellular localization, and intracellular signaling converge to reshape synaptic plasticity, circuit function, and disease progression. In contrast to earlier frameworks that considered glutamatergic dysfunction in broad or ionotropic terms, accumulating evidence now highlights that distinct mGluR subtypes (Groups I–III) exert region‐, cell type–, and context‐dependent effects through defined signaling pathways, including Gq/PLC/IP_3_/Ca^2+^, Gi/o–mediated inhibition of cAMP, and downstream regulation of ERK, mTOR, and translational control. These pathways critically influence both neuronal and glial plasticity mechanisms, thereby linking receptor‐level perturbations to systems‐level dysfunction. In Section 10, we systematically examine subtype‐specific mGluR dysregulation across neurological and neurodegenerative conditions, integrating molecular, synaptic, and network‐level evidence (Table 7). The disorders discussed span neurodevelopmental (ASD and FXS), psychiatric (schizophrenia and PTSD), and classical neurodegenerative diseases (AD, PD, Huntington’s disease [HD], and ALS), as well as epilepsy and neuropathic pain, thereby capturing the broad functional spectrum of mGluR–mediated plasticity disturbances. As illustrated in Figure 3, these conditions exhibit convergent patterns of receptor dysregulation at the membrane and synaptic level, including altered receptor density, trafficking, and coupling to intracellular signaling cascades, which collectively shift the balance between LTP and LTD. Complementarily, Figure 4 summarizes how these subtype‐specific alterations propagate across cellular compartments and brain regions to produce disease‐specific phenotypes, highlighting interactions between neuronal, astrocytic, and microglial mGluR signaling within larger circuit architectures. By organizing the evidence around receptor subtype, signaling mechanism, and disease context, Section 10 aims to clarify how mGluR dysfunction transitions from a molecular perturbation to a driver of pathological plasticity. This framework also provides a foundation for evaluating subtype‐selective pharmacological strategies, emphasizing that therapeutic efficacy will likely depend on targeting specific mGluR subtypes within defined cellular and circuit contexts rather than applying uniform glutamatergic modulation across disorders.

**Table 7:** Subtype‐specific mGluR dysregulation across neurodegenerative disorders.

Disorder	Key mGluR subtypes	Regional/subcellular distribution	Intracellular signaling mechanisms	Synaptic plasticity (LTP/LTD)	Neuron–glia interaction	Functional outcome	Pharmacological targeting	Key references
ASD	mGluR5	Increased cortical mGluR5 in idiopathic ASD; higher binding in left striatum/thalamus; mainly postsynaptic, also present on glia	mGluR5 modulates glutamate/GABA signaling, interacts with NMDARs, and affects presynaptic Ca^2+^; mTOR and MAPK/ERK pathways are noted (from FXS), not directly shown in ASD	Not assessed in ASD; mGluR–dependent LTD mentioned only in the FXS context, not measured in ASD cohorts	mGluR5 is present on neurons and glia; neuron–glia interactions (e.g., CNTNAP2–related) are discussed, but no direct evidence of altered mGluR signaling in ASD	Increased mGluR5 in cortex and left striatum/thalamus; negatively correlates with GABA+; elevated glutamate and reduced GABA receptors in ASD support E/I imbalance (not mGluR–specific)	mGluR5 PET imaging ([18F]FPEB) enables in vivo quantification and stratification; therapeutic targeting remains investigational with mixed outcomes in ASD	[75–77]
FXS	mGluR5	FXS PET studies show reduced mGluR5 in men across cortical/subcortical regions (notably occipital cortex and thalamus); background: postsynaptic receptor regulating astrocytic glutamate signaling	Loss of FMRP dysregulates mGluR–mediated protein synthesis; it involves mTOR, MAPK/ERK, PKA, and GSK3 pathways	mGluR–LTD is enhanced and protein synthesis–independent in Fmr1‐/y; no net spine shrinkage in WT or Fmr1‐/y, indicating dissociation of functional versus structural plasticity	mGluR5 knockout (FXS–like) shows increased microglial reactivity and altered inflammatory/ECM markers; astrocytic glutamate regulation is background mGluR5 biology	FXS shows dysregulated protein synthesis, synaptic dysfunction, and hyperexcitability; models exhibit seizures, cortical hyperexcitability, and behavioral deficits	mGluR5 NAMs show strong preclinical effects, but mavoglurant/basimglurant lack clear clinical efficacy; tolerance may contribute; [18F]FPEB PET is useful as a biomarker	[78–81]
Schizophrenia	mGluR5	mGluR5 is abundant in cortex, hippocampus, and striatum; mGlu1/5 present in PV + interneurons (DLPFC); studies focus on DLPFC (and hippocampus in one); reduced mGlu2R (DLPFC) and mGlu3R (in antipsychotic‐free schizophrenia)	mGluR5 enhances NMDAR function; schizophrenia involves coordinated glutamatergic changes (not a single receptor); Group II (esp. mGlu3R) show epigenetic and antipsychotic‐related effects in DLPFC	Schizophrenia involves altered plasticity and connectivity; mGluR5 is linked to LTP/LTD and excitability, but not directly measured in patients	Group I mGluRs in DLPFC interneurons; mGlu3R also in glia; neuroinflammation shown in Poly I:C model, not directly in human schizophrenia tissue	Lower mGluR5 availability in schizophrenia is linked to worse negative/depressive symptoms, poorer functioning, and impaired cognition; no overall case–control difference in binding was seen on PET, but broader glutamatergic dysregulation in the DLPFC/hippocampus is supported by postmortem data	mGlu5 PAMs show procognitive and antipsychotic‐like effects; mGlu2/3 therapies may be limited by reduced cortical receptors; antipsychotics may normalize mGlu3R but not mGlu2R in DLPFC	[82–85]
Epilepsy	mGluR1, mGluR5 (Group I)	Group I mGluRs are mainly postsynaptic; II/III largely presynaptic, with mGluR3 also in glia; region‐ and phase‐specific changes occur in hippocampus/temporal cortex in epilepsy, with altered cortical mGluR5 networks in focal cortical dysplasia	Group I mGluRs (Gq/G11) activate PLC–IP_3_/DAG → Ca^2+^/PKC and enhance NMDA; in PTZ SE, mGluR1 boosts excitability via cAMP/PKA (HCN1 suppression). Groups II/III inhibit adenylate cyclase and neurotransmitter release	mGluRs regulate synaptic transmission relevant to plasticity/epileptogenesis, but patient LTP/LTD isn’t directly measured; models link altered mGluRs to hyperexcitability rather than quantified LTP/LTD deficits	Astrocytes modulate seizures via EAAT2 uptake and mGluR–mediated glutamate release; glial mGluR3 expression is also noted	mGluR dysregulation drives hyperexcitability and seizures; focal cortical dysplasia shows reduced mGluR5 network integration, with models supporting epileptogenic expression changes	In PTZ SE, mGluR1 worsens seizures, and its antagonism reduces them; in lithium–pilocarpine models, Group III mGluRs show preclinical promise for preventing epileptogenesis	[86–89]
Alzheimer’s disease	mGluR5	mGluR5 is mainly postsynaptic (also pre‐/nuclear), with reduced hippocampal/parahippocampal levels in AD; mGluR1α is dendritic/plasma membrane–localized and reduced in CA1, subiculum, and entorhinal cortex	Group I mGluRs (Gq/PLC/IP_3_/DAG) drive Ca^2+^ signaling; in AD, abnormal mGluR5 (Aβ‐mGluR5/Fyn/tau) and eEF2K/eEF2 pathways are linked to impaired mGluR–LTD (APP/PS1)	AD involves impaired plasticity; hippocampal mGluR–LTD is reduced in APP/PS1 mice and rescued by eEF2K suppression, while LTP deficits are noted but not directly tested	Neuroinflammation‐linked glial mGluR5 upregulation is noted, but altered neuron–glial mGluR signaling is not directly measured in AD patients	Region‐ and group‐specific links exist between mGluR5 and amyloid, medial temporal atrophy, plasma p‐tau181/NfL, and cognition (no overall correlation); mGluR1α is reduced in vulnerable medial temporal regions with unclear functional impact	mGluR5 is a potential neurodegenerative biomarker (therapeutic relevance investigational); eEF2K suppression rescues hippocampal mGluR–LTD in APP/PS1 mice, highlighting translational control as a target	[90–92]
Parkinson’s disease	mGluR5	mGluR5 is enriched in striatal MSNs/interneurons; mGluR2/3 are presynaptic on corticostriatal, cholinergic, GABAergic, and pallidal neurons, with mGluR3 also in glia	mGluR5 drives excessive glutamatergic signaling in PD (6‐OHDA: ↑ intra‐axonal Ca^2+^, calpain, ERK); Group II mGluRs inhibit adenylyl cyclase and modulate voltage‐gated Ca^2+^/K^+^ channels	Evidence supports altered glutamatergic signaling in PD, but without direct LTP/LTD measures; plasticity claims should remain general, not direct	mGluR3 is expressed in both neurons and glia, but direct altered neuron–glia interaction was not demonstrated in these papers	Nigrostriatal dopamine loss is associated with excessive glutamatergic activity contributing to motor symptoms, dyskinesias, and some non‐motor symptoms; in the 6‐OHDA model, increased mGluR5 is associated with early axonal degeneration	mGluR5 NAMs (e.g., dipraglurant) improve motor/dyskinesia and some nonmotor symptoms; MPEP/MTEP reduce mGluR5–linked axonal degeneration (6‐OHDA); mGluR2/3 strategies may help wearing‐off fluctuations	[93–95]
Huntington’s disease	mGluR5	mGluR5 is abundant in striatum/cortex (HD–vulnerable regions); Q175 PET shows reduced binding there, confirmed postmortem	mGluR5 activation drives IP_3_–Ca^2+^/PKC signaling, enhanced by mutant huntingtin; dysregulated PI3K/PDK1/Akt/mTOR impairs autophagy, while mGluR5 also modulates CREB/BDNF and REST/NRSF via Src and N‐cadherin/β‐catenin pathways	Evidence supports altered neuronal signaling and gene expression in HD, with synaptic dysfunction inferred from dysregulated transcriptional and Ca^2+^ pathways, not direct LTP/LTD measures	No direct neuron‐glia interaction changes are shown; mGluR5 involvement in peripheral inflammation/metabolism (BACHD mice) should not be taken as CNS neuron–glia evidence	mGluR5 dysregulation links to mHTT aggregation, impaired autophagy, transcriptional changes, neuronal loss, and motor/cognitive deficits; reduced binding in Q175 mice worsens overtime	mGluR5 NAM CTEP improves HD pathology in mice: reduces mHTT aggregates, normalizes PI3K/Akt/mTOR, enhances ULK1/autophagy and CREB/BDNF, and rescues REST/NRSF abnormalities	[96–99]
ALS	mGluR1, mGluR5	Group I mGluRs are upregulated in ALS spinal cord; mGluR1/5 increase in glutamatergic terminals early, with mGluR5 also in astrocytes and microglial Group I mGluRs linked to neuroinflammation	Group I mGluRs (PLC/IP_3_/DAG) trigger Ca^2+^ release and PKC; in SOD1G93A, 3,5‐DHPG–evoked glutamate release is exocytotic via intraterminal Ca^2+^, with PLC/PKC, MAPK/ERK, and Akt/GSK–3β/CREB pathways also implicated	Not directly assessed as LTP/LTD in these papers; instead, Group I mGluR overactivity enhances excitatory glutamate transmission and abnormal presynaptic glutamate release in the spinal cord during disease progression	Mutant SOD1 astrocytes are glutamate‐vulnerable and degenerate via mGluR5; reducing mGluR5 dampens astrocyte/microglial activation, with microglial Group I mGluRs modulating reactive and inflammatory states	Excessive glutamatergic transmission/excitotoxicity contributes to motor neuron degeneration and disease progression; mGluR5 reduction preserves motor neurons, lowers cytosolic Ca^2+^, delays onset, prolongs survival probability, and normalizes abnormal glutamate release in SOD1G93A mice	Blocking Group I mGluRs, especially mGluR5, benefits ALS models: delays onset, extends survival, preserves motor neurons, normalizes glutamate release, and reduces astrocyte/microglial activation; in vivo blockade also slows astrocytic degeneration	[100–103]
Neuropathic pain	mGluR5, mGluR1	mGluR5 is upregulated in caudal prelimbic mPFC after SNI; it is tonically active in PAG neurons, and mGluR1/5 in the red nucleus increase contralateral to injury	In PAG, persistent mGluR5 sustains excitability/Ca^2+^ oscillations; Homer1a disrupts this in neuropathic pain, with knockdown preventing chronic pain after SNL; in the red nucleus, mGluR1/5 drive TNF‐α and IL‐1β expression	LTP/LTD was not directly assessed; the studies instead focus on maladaptive supraspinal sensitization and altered mGluR–dependent excitability in pain‐modulatory circuits	Neuroimmune signaling is implicated in the red nucleus through mGluR1/5–dependent increases in TNF‐α and IL‐1β; broader neuron–glia mechanisms were not directly established across all regions studied	Prelimbic mGluR5 upregulation drives tactile hypersensitivity and depressive‐like behavior; a single PAG mGluR5 inverse agonist (MPEP) induces long‐lasting allodynia; red nucleus mGluR1/5 promote mechanical allodynia	Prelimbic mGluR5 blockade (MPEP/MTEP) reduces allodynia and depressive‐like behavior; PAG mGluR1/5 agonism (DHPG) relieves neuropathic pain, while mGluR5 inverse agonism induces persistent pain; red nucleus LY367385/MTEP alleviate SNI pain	[104–106]
PTSD	mGluR2	mGluR2 is chronically elevated presynaptically in cortex, hippocampus, and amygdala after blast exposure; cortical mGluR5 availability is increased in PTSD, strongest in PFC, with additional ventral striatal elevation	Group II antagonism reverses blast‐induced PTSD–like traits, implicating pathological mGluR2 signaling; mGluR5 changes (↑SHANK1, ↓FKBP5) suggest altered anchoring and glucocorticoid regulation	These papers discuss PTSD in the context of glutamate‐dependent fear, memory, and stress circuitry; direct LTP/LTD measurements were not reported	No direct neuron–glia mechanism is shown; evidence centers on neuronal receptor dysregulation, behavioral phenotypes, and stress‐related circuit changes	Chronic mGluR2 elevation links to persistent PTSD–like traits (impaired recognition memory, abnormal fear), while higher cortical mGluR5 correlates with avoidance symptoms	mGluR2/3 antagonists show benefit: LY341495 reverses memory/fear deficits, BCI‐838 improves PTSD–like traits and boosts dentate gyrus neurogenesis; imaging/postmortem data also support mGluR5 as a target	[107–110]
Stroke/ischemia	mGluR5	mGluR5 is widely expressed, mainly postsynaptic on pyramidal neurons, also on GABAergic neurons and glia; white‐matter astrocytes express mGluR5 with Group I‐dependent responses in ischemia	Group I mGluRs (Gq/PLC/IP_3_) drive Ca^2+^ signaling; after stroke, mGluR5 contributes to maladaptive plasticity/network dysfunction, while in white‐matter astrocytes, activation evokes Ca^2+^ signaling and is protective against OGD	mGluR5 regulates synaptic transmission and plasticity; poststroke, it drives maladaptive plasticity affecting recovery rather than directly measured LTP/LTD	Astrocytic Group I mGluR activation induces Ca^2+^ signaling and protects white‐matter astrocytes from ischemia; broader neuron–glia conclusions remain cautious	Stroke disrupts brain‐wide connectivity; mGluR5–driven maladaptive plasticity impairs recovery, while its inhibition restores connectivity and sensorimotor function	mGluR5 NAMs (MTEP, fenobam, AFQ056) improve poststroke recovery; PAMs impair it, and combining inhibition with enriched environment further enhances outcomes	[111, 112]

### 10.1. ASD

ASD illustrates how mGluR dysregulation and broader glutamatergic–GABAergic imbalance can converge on synaptic plasticity mechanisms that shape circuit development, neuronal excitability, and ultimately complex behavioral phenotypes. Across clinical, imaging, animal, and genetic studies, alterations in mGluR5 signaling emerge alongside disrupted excitation–inhibition balance and micronutrient homeostasis, suggesting that multiple converging molecular pathways can perturb glutamatergic synapses and their plasticity rules in ASD, with important implications for stratified therapeutic strategies targeting specific mGluR subtypes and associated signaling networks.

A recent study by Meguid et al. [75] investigated neurochemical alterations associated with ASD by examining biomarkers of inhibitory and excitatory neurotransmission, as well as essential mineral homeostasis, in children. The analysis reveals a pronounced disruption in the balance between inhibitory GABAergic signaling and excitatory glutamatergic activity, as reflected by significantly reduced serum levels of GABAA and GABAB receptors and elevated glutamate concentrations in children with ASD compared to neurotypical controls. This neurochemical imbalance supports the hypothesis that ASD pathology involves dysregulation of the excitation–inhibition equilibrium within neural circuits, which is essential for maintaining normal synaptic transmission, neural development, and behavioral regulation. In addition to neurotransmitter abnormalities, the study identifies significant reductions in key mineral elements, including zinc, potassium, and calcium, suggesting that ionic and micronutrient homeostasis may contribute to altered neuronal excitability and synaptic signaling in ASD. Correlation analysis further indicates that lower levels of GABAB receptors and zinc are associated with increased repetitive and stereotypical behaviors, highlighting their potential relevance as biomarkers linked to behavioral severity. Collectively, these findings suggest that ASD may arise from interacting neurochemical and metabolic disturbances affecting neurotransmission, synaptic regulation, and neural signaling pathways, emphasizing the importance of integrating neurotransmitter and mineral biomarker analysis to better understand the pathophysiology of the disorder and identify potential therapeutic targets [75].

Building on this evidence for excitation–inhibition imbalance, Brašić et al. [76] offers a detailed analysis of mGluR5 expression across idiopathic ASD (IASD), FXS, and typically developing individuals using PET imaging with the radioligand [18F]FPEB. The results reveal a clear and important pattern: mGluR5 expression is significantly increased in the cortical regions of individuals with IASD, while it is markedly reduced across both cortical and subcortical regions in individuals with FXS. This opposing trend highlights that ASD is not a single uniform condition but rather a spectrum with distinct underlying neurobiological mechanisms, particularly when comparing genetically defined disorders like FXS with idiopathic cases. The findings support the role of mGluR5 in synaptic signaling and neurodevelopment, with reduced expression in FXS potentially contributing to cognitive deficits and behavioral abnormalities, while increased expression in IASD may reflect altered excitatory signaling pathways. However, the study is not without its limitations, including a small sample size, variability in imaging protocols between research sites, and confounding factors such as differences in age and intellectual ability between groups. These issues complicate the interpretation of whether observed differences are purely disease‐related or influenced by cohort characteristics. Despite these constraints, the study successfully demonstrates the potential of PET imaging as a tool for measuring mGluR5 in vivo, offering a promising direction for biomarker development and precision medicine approaches in ASD. In short, the study cuts through the usual vague theorizing and actually shows that different forms of autism may operate through entirely different molecular pathways, which is inconveniently complex but scientifically honest [76].

Further extending the link between mGluR5 signaling and excitation–inhibition balance, Carey et al. [77] adopt a translational approach to investigate the relationship between mGluR5 availability and the balance between excitatory and inhibitory neurotransmission in ASD. Using a combination of PET with the radioligand [18F]FPEB and proton magnetic resonance spectroscopy (1H‐MRS), the authors directly examined the relationship between mGluR5 receptor availability and regional levels of glutamate–glutamine (Glx) and GABA+ in the human brain. The analysis focused on two regions implicated in ASD pathology: the dorsomedial prefrontal cortex and a region encompassing the left striatum and thalamus. PET results demonstrated significantly higher mGluR5 availability in individuals with ASD compared with controls, particularly within the striatum/thalamus region, suggesting enhanced glutamatergic receptor signaling in neural circuits involved in social cognition and behavioral regulation. Importantly, correlation analyses revealed a strong negative relationship between GABA+ levels and mGluR5 availability across participants, indicating that increased mGluR5 expression may be associated with reduced inhibitory neurotransmission. To explore the molecular basis of these findings, the study incorporated autoradiography experiments in three mouse models associated with ASD (Cntnap2 KO, Shank3 KO, and 16p11.2 deletion). Among these models, only Cntnap2 KO mice exhibited significantly increased striatal mGluR5 binding, mirroring the human imaging results. Since this model is characterized by deficits in parvalbumin‐positive GABAergic interneurons, the findings suggest that elevated mGluR5 availability in ASD may represent a compensatory response to disruptions in inhibitory neural circuits. Overall, the study highlights a potential mechanistic link between glutamatergic receptor signaling and GABAergic dysfunction, supporting the hypothesis that excitation–inhibition imbalance plays a central role in ASD neurobiology [77].

Together, the ASD studies in this section illustrate that mGluR–related dysregulation in neurodegenerative and neurodevelopmental conditions rarely acts in isolation but instead emerges at the intersection of altered receptor expression, disrupted excitation–inhibition balance, and genetically driven changes in synaptic architecture and plasticity. At the same time, several important limitations temper the strength of the conclusions that can be drawn, including small and often heterogeneous clinical samples, cross‐sectional designs that preclude causal inference, variability in imaging and analytical protocols across sites, and the use of diverse animal and cellular models that only partially recapitulate human disease. These constraints, combined with the marked molecular and phenotypic heterogeneity within and between disorders, suggest that current findings should be interpreted as identifying candidate mechanisms and mGluR–related signaling pathways rather than definitive disease biomarkers or therapeutic targets. Future work should, therefore, prioritize larger, longitudinal, and mechanistically informed studies that integrate receptor imaging, molecular profiling, and circuit‐level readouts of synaptic plasticity, ideally across stratified patient subgroups defined by genetic background, clinical phenotype, or treatment response. Such approaches may help to disentangle compensatory from pathogenic mGluR adaptations, clarify how receptor subtype‐ and region‐specific changes interact with ionotropic plasticity mechanisms, and support the rational development of subtype‐selective mGluR modulators or circuit‐targeted interventions with improved translational potential and safety profiles.

### 10.2. FXS

mGluR5 has emerged as a central node linking synaptic signaling, circuit maturation, and neuroimmune function to the cognitive and behavioral phenotypes of FXS, providing a translational bridge between basic mechanisms of mGluR–dependent plasticity and clinically relevant biomarkers and treatment strategies. In this context, converging human imaging, cellular plasticity, pharmacological, and genetic studies collectively indicate that FMRP deficiency and mGluR5 dysregulation in FXS cannot be captured by a simple model of uniformly increased receptor signaling but instead involve region‐specific alterations in receptor expression, downstream translational control, and neuroinflammatory pathways with direct implications for therapeutic targeting.

In FXS, converging human imaging, synaptic plasticity, pharmacological, and genetic data collectively indicate that FMRP deficiency leads to complex mGluR5 dysregulation that cannot be captured by a simple “excess mGluR5 signaling” model. Brašić et al. [78] investigated the relationship between FMRP levels and cerebral expression of mGluR5 in men with FXS, aiming to clarify inconsistencies in previous research regarding glutamatergic dysfunction in the disorder. Using PET with the radioligand [18F]FPEB, they quantified mGluR5 expression across multiple cortical and subcortical brain regions and compared these measurements with peripheral levels of FMRP obtained from lymphocyte assays. Contrary to predictions derived from the traditional mGluR theory of FXS, which suggests excessive mGluR5 signaling due to the absence of FMRP regulation, their results demonstrated reduced mGluR5 expression throughout the brains of affected individuals. Linear regression analysis further indicated that mGluR5 expression was significantly lower in regions such as the occipital cortex and thalamus when FMRP levels were held constant, suggesting region‐specific modulation of glutamatergic signaling pathways. These findings challenge simplified interpretations of the mGluR hypothesis and highlight the complexity of the relationship between FMRP deficiency and glutamatergic receptor regulation in humans. By combining molecular measurements of FMRP with in vivo imaging of receptor expression, the study proposes a biomarker framework that may improve the design of clinical trials targeting glutamatergic pathways in FXS and related autism spectrum conditions [78].

At the synaptic level, Thomazeau et al. [79] addressed how mGluR5–dependent plasticity is altered in Fragile X by dissecting the relationship between functional and structural synaptic changes. Synaptic plasticity involves both functional changes in synaptic transmission and structural modifications of dendritic spines, yet the relationship between these processes remains poorly understood. Thomazeau et al. [79] demonstrated that long‐term synaptic depression (LTD) induced by activation of NMDARs and mGluRs produces distinct structural outcomes despite producing similar functional weakening of synaptic transmission. Using simultaneous electrophysiological recordings and two‐photon imaging of dendritic spines in hippocampal CA1 neurons, they observed that activation of NMDARs produced both synaptic depression and persistent spine shrinkage, indicating coordinated functional and structural plasticity. In contrast, activation of mGluR5 using the agonist DHPG induced robust LTD and internalization of AMPA receptors but failed to produce lasting structural changes in dendritic spines, indicating that functional synaptic depression does not necessarily require accompanying morphological remodeling. Further experiments showed that NMDA–induced spine shrinkage depends on nonionotropic (metabotropic) signaling through NMDARs and requires mTORC1–dependent protein synthesis, whereas the electrophysiological expression of LTD depends on ion flux through the receptor channel. Importantly, in the Fragile X mouse model lacking FMRP, structural plasticity no longer required mTORC1 activation or new protein synthesis, suggesting that elevated basal protein synthesis removes regulatory constraints on spine remodeling. Together, these findings reveal that functional and structural plasticity are governed by partially independent signaling pathways and that dysregulation of translational control mechanisms in FXS alters the structural dynamics of synapses in an mGluR5–related manner [79].

Building on these mechanistic insights, Stoppel et al. [80] examined how chronic pharmacological manipulation of mGluR5 impacts Fragile X–like phenotypes overtime, focusing on acquired treatment resistance. Their study critically evaluates the limitations of targeting mGluR5 as a therapeutic strategy for FXS by assessing the emergence of tolerance following repeated administration of the mGluR5 NAM CTEP in the Fmr1 KO mouse model. The authors showed that chronic CTEP rapidly produces tolerance, reducing its ability to rescue key Fragile X phenotypes, including audiogenic seizure susceptibility, cortical hyperexcitability, and elevated hippocampal protein synthesis. Experimental evidence across behavioral, electrophysiological, and biochemical assays indicated that this resistance does not arise from increased mGluR5 receptor expression or sensitivity, but instead reflects intracellular adaptations within the downstream signaling cascade linking mGluR5 activation to protein synthesis. Pharmacological experiments further suggested that the adaptive mechanism occurs downstream of glycogen synthase kinase‐3α (GSK3α) but upstream of translational control processes, as inhibition of protein synthesis with cycloheximide remained effective even after resistance to mGluR5 inhibition had developed. Importantly, the authors also demonstrated that brief inhibition of mGluR5 during early postnatal development can produce persistent improvements in cognitive behavior measured weeks after treatment cessation, indicating the existence of critical developmental windows during which therapeutic intervention can restore more normal neural circuit maturation. These data, therefore, suggest that, in the context of FXS, the efficacy of mGluR5–targeted therapies depends not only on the magnitude of receptor inhibition but also on timing and dosing strategies that avoid maladaptive downstream compensation [80].

Finally, Dahl et al. [81] extended the scope from synapses and circuits to neuroimmune and extracellular matrix mechanisms by evaluating the pathological consequences of disrupting mGluR5 signaling in a genetically engineered rat model. Their study characterized heterozygous mGluR5 KO rats to investigate mechanisms potentially relevant to FXS and related ASDs and demonstrated that partial loss of mGluR5 produces developmental abnormalities accompanied by pronounced neuroinflammatory responses. Molecular analyses revealed significant alterations in inflammatory cytokine expression, including elevated levels of chemokines such as CXCL1 and CXCL2, indicating enhanced inflammatory signaling during early postnatal development. Histological and imaging analyses further showed a substantial increase in microglial density within cortical and hippocampal regions, particularly in postnatal day 14 animals, suggesting early activation of neuroimmune pathways. Morphological analysis of individual microglial cells demonstrated age‐dependent changes in cell area, perimeter, circularity, and branching complexity, reflecting shifts from immature to more ramified microglial states during development. These changes were accompanied by alterations in extracellular matrix–related gene expression and perineuronal net density, implying that mGluR5 signaling influences structural remodeling of neural circuits through immune‐mediated pathways. Behavioral assays, including open‐field and elevated‐plus maze tests, also revealed abnormal anxiety‐like and exploratory behaviors in heterozygous animals relative to wild‐type controls. Collectively, the study suggests that disruption of mGluR5 signaling contributes not only to synaptic dysregulation but also to neuroinflammatory and extracellular matrix alterations that may shape the development of neuropsychiatric phenotypes associated with FXS and ASD [81].

Together, these studies indicate that mGluR5 dysregulation in FXS encompasses alterations in receptor expression, decoupling of functional and structural plasticity, adaptive changes in translational signaling, and neuroinflammatory remodeling, rather than a uniform gain‐of‐function of mGluR5 signaling. At the same time, important limitations remain: most data derive from preclinical rodent models or relatively small human cohorts, cross‐sectional designs constrain inferences about developmental trajectories, and methodological differences in imaging, behavioral assessment, and plasticity protocols complicate direct comparison across studies. Moreover, current work focuses predominantly on mGluR5 within hippocampal and select cortical circuits, leaving the contribution of other mGluR subtypes, subcortical regions, and cell‐type–specific signaling (e.g., interneurons, astrocytes, and microglia) incompletely defined. Future studies combining longitudinal in vivo imaging, cell‐type–resolved manipulations, and pathway‐specific pharmacology across developmental stages will be essential to clarify how mGluR–dependent synaptic plasticity, translational control, and neuroimmune signaling interact to shape disease progression. Such integrative approaches may refine biomarker strategies, optimize the timing and pattern of mGluR–targeted interventions, and support the design of rational combination therapies that more effectively translate mechanistic insights on mGluR dysregulation into durable clinical benefit in FXS and related neurodevelopmental disorders.

### 10.3. Schizophrenia

Schizophrenia exemplifies how mGluR dysregulation contributes to neuropsychiatric symptomatology through distributed, circuit‐level alterations rather than isolated receptor changes. Converging postmortem, in vivo imaging, and preclinical data indicate that disturbances in Groups I and II mGluR signaling intersect with NMDAR function, synaptic scaffolding complexes, neuroimmune pathways, and environmental modulators such as nicotine and antipsychotic exposure, thereby linking molecular pathology to cognitive, affective, and sensorimotor deficits characteristic of the disorder. In postmortem dorsolateral prefrontal cortex and hippocampus, multilevel molecular profiling combined with machine learning indicates that schizophrenia is better characterized by coordinated perturbations across glutamatergic synapses than by single, large‐effect molecular changes. Univariate analyses of neuroactive amino acids (including glutamate, D‐serine, glycine, and asparagine), as well as transcripts and proteins for NMDA (GluN1 and GluN2A/B) and AMPA (GluA1–4) receptor subunits, metabotropic receptors (mGluR1–5), synaptic scaffolding proteins (PSD‐95 and Homer1), and glutamate transporters (EAAT1/2 and VGluT1/2), revealed only limited isolated differences that did not survive correction for multiple comparisons. However, multivariate models identified combinations of NMDAR signaling components such as GluN1 and its coagonist D‐serine, postsynaptic structural elements including PSD‑95 and GluA1, and glutamate regulatory systems involving mGluR2/3, mGluR5, and EAAT2 as collectively contributing to molecular signatures that discriminated schizophrenia from control brains. These results suggest that mGluR–containing signaling modules participate in broader glutamatergic network disruptions that underlie synaptic dysregulation in schizophrenia [82].

In vivo molecular imaging further links regional mGluR5 availability to clinical and cognitive manifestations of the disease. Using [^11^C]ABP688 PET in male patients with chronic schizophrenia, regional mGluR5 binding potential across the prefrontal and temporal cortices, caudate, putamen, and inferior frontal gyrus was examined in relation to psychopathology and cognitive performance rather than as a categorical diagnostic marker [83]. While overall mGluR5 binding did not differ significantly between patients and healthy controls, higher receptor availability was associated with reduced negative and depressive symptom severity on PANSS, SANS, and HAMD scales, improved global functioning, and better performance on neuropsychological tests, with particularly robust correlations in the left temporal cortex and caudate where lower binding was linked to greater symptom burden and poorer functioning [83]. The study also identified smoking as a major modulator of mGluR5, with markedly reduced binding, approaching 50% decreases in some regions, in smokers irrespective of diagnostic group, indicating that nicotine exposure can substantially influence mGluR5–mediated glutamatergic signaling [83]. These findings support a role for regional mGluR5 availability in shaping symptom expression and cognitive profiles in schizophrenia, while underscoring the need to account for environmental factors such as nicotine use when interpreting mGluR–related imaging data [83].

Preclinical work in maternal immune activation models provides mechanistic and translational insights into how Group I mGluRs may contribute to schizophrenia‐like phenotypes and their potential reversibility. In a Poly I:C–induced model, offspring exhibited robust impairments in prepulse inhibition, novel object recognition, and Y‑maze spontaneous alternation, aligning with sensorimotor gating and memory deficits observed clinically [84]. Pharmacological enhancement of mGlu1 and mGlu5 signaling with positive allosteric modulators (PAMs), particularly the mGlu5 PAM VU‑29 and the mGlu1 PAM RO 67‑7476, significantly reversed these behavioral abnormalities during both adolescence and adulthood, while concurrently reducing neuroinflammatory markers such as TNF‑α and IL‑1β in the prefrontal cortex and hippocampus [84]. In contrast, NAMs, including fenobam and JNJ‑16259685, produced minimal or inconsistent effects on behavioral outcomes [84]. Together, these data suggest that enhancing, rather than inhibiting, Group I mGluR signaling can ameliorate schizophrenia‐like cognitive and sensorimotor disturbances in the context of immune‐related developmental insults, and point to an interaction between glutamatergic and neuroimmune pathways that may be relevant to disease pathophysiology [84].

Complementary evidence from human postmortem cortex highlights disease‐ and treatment‐related alterations in Group II mGluRs and the accompanying epigenetic landscape. In DLPFC samples from a large cohort of individuals with schizophrenia and matched controls, a significant reduction in cortical mGlu2R protein levels was observed in schizophrenia across both medicated and unmedicated subjects, suggesting that decreased mGlu2R expression reflects a disease‐associated change rather than a secondary effect of antipsychotic treatment [85]. In contrast, mGlu3R downregulation was most evident in antipsychotic‐free patients, whereas medicated individuals showed receptor levels closer to those of controls, consistent with a potential normalizing influence of antipsychotic exposure on mGlu3R expression [85]. These protein‐level alterations were not paralleled by straightforward transcriptional changes: GRM2 mRNA remained unchanged, and GRM3 transcripts were paradoxically increased in unmedicated patients, while chromatin immunoprecipitation assays demonstrated increased permissive histone marks at the GRM3 promoter, indicative of epigenetic mechanisms that may act to compensate for receptor loss [85]. Supporting a link between developmental insults, treatment, and Group II mGluR regulation, rodent studies showed that prenatal immune activation reduced cortical mGlu2R levels and that chronic clozapine administration further decreased mGlu2R expression [85]. On this basis, the authors propose that schizophrenia‐associated reductions in Group II mGluR function engage epigenetic feedback processes attempting to restore signaling, while antipsychotic medications may differentially modulate mGlu2R and mGlu3R trajectories [85].

Collectively, these studies indicate that schizophrenia involves distributed, context‐dependent alterations in Groups I and II mGluR signaling that are embedded within broader glutamatergic, synaptic, immune, and environmental networks, rather than simple unidirectional changes in isolated receptor subtypes. At the same time, important limitations constrain interpretation: postmortem and PET datasets are largely cross‐sectional and male‐biased, sample sizes and regional coverage vary, smoking and medication status remain major confounders, and preclinical MIA models only approximate selected dimensions of the disorder. Future work should prioritize longitudinal, multimodal designs that integrate in vivo mGluR imaging with detailed clinical phenotyping and genetic/epigenetic profiling, refine developmental and immune‐related animal models to better capture human disease trajectories, and systematically test subtype‐selective mGluR modulators (including PAMs and NAMs) in relation to specific symptom clusters and cognitive domains, ideally in combination with stratification by environmental exposures such as nicotine use and antipsychotic treatment history.

### 10.4. Epilepsy

Dysregulation of mGluRs contributes to epilepsy at multiple, interconnected levels of neuronal circuitry, ranging from receptor‐ and channel‐specific signaling in hippocampal pyramidal neurons to large‐scale reconfiguration of glutamatergic receptor networks and neuron–glia interactions across cortical circuits. In the context of status epilepticus (SE), excessive glutamatergic drive can aberrantly engage Group I mGluRs, particularly mGluR1, leading to maladaptive modulation of intrinsic conductances and loss of homeostatic control over neuronal excitability. At the same time, epileptogenesis involves dynamic, stage‐ and region‐specific remodeling of multiple mGluR subtypes (Group I–III) in hippocampal and cortical regions, which shifts the balance between excitatory transmission and presynaptic inhibition as the disease progresses from latent to chronic phases. Beyond neurons, astrocytic mGluRs and glutamate transporters critically shape extracellular glutamate dynamics, and thereby influence seizure thresholds, firing patterns, and network stability. Finally, these molecular and cellular alterations manifest at the systems level as disturbed mGluR5 network topology and reduced cortical integration in patients with focal epileptic pathologies, underscoring that mGluR dysregulation in epilepsy is not restricted to focal lesions but involves widespread plastic reorganization of glutamatergic signaling across the brain.

In a mechanistic study focused on the hippocampal CA1 region during SE, activation of mGluR1 was shown to regulate neuronal excitability by selectively disrupting hyperpolarization‐activated cyclic nucleotide‐gated (HCN) channel function [86]. Using a pentylenetetrazole (PTZ)‐induced rat model of SE, this work demonstrated a marked reduction in HCN1 channel expression, particularly in membrane‐bound surface proteins, whereas HCN2 expression was largely preserved, indicating a subtype‐specific impairment of HCN1–mediated control of excitability [86]. Concurrently, mGluR1 expression increased in the CA1 region and co‐localized with HCN1 in pyramidal neurons, suggesting a functional interaction in which heightened mGluR1 signaling influences HCN1 trafficking and availability. Pharmacological activation of mGluR1 with the agonist DHPG further suppressed HCN1 expression and exacerbated seizure severity and mortality, whereas mGluR1 inhibition by LY367385 restored HCN1 levels, delayed seizure onset, and reduced seizure severity, directly linking mGluR1 activity to epileptic outcomes. At the intracellular level, mGluR1 activation engaged the cAMP–PKA pathway, which interfered with TRIP8b–dependent regulation of HCN1 surface trafficking, thereby decreasing HCN1 membrane availability; blockade of PKA with H89 reversed these effects, normalizing HCN1 expression and attenuating seizures. Together, these findings indicate that, in epilepsy, excessive glutamatergic signaling amplifies hippocampal excitability through an mGluR1–cAMP–PKA cascade that impairs HCN1 channel function, highlighting this receptor–channel axis as a potential therapeutic node for modulating neuronal excitability in SE [86].

Expanding from this receptor–channel interaction to broader transcriptomic remodeling, changes in mGluR gene expression across the course of epileptogenesis have been characterized using a lithium–pilocarpine rat model of temporal lobe epilepsy [87]. Rather than restricting the analysis to a single subtype, this study systematically quantified mRNA levels of Group I (Grm1 and Grm5), Group II (Grm2 and Grm3), and Group III (Grm4, Grm7, and Grm8) receptors in the dorsal and ventral hippocampus and temporal cortex at both latent (3 and 7 days) and chronic (60 days) stages after seizures, thereby capturing the temporal evolution of mGluR regulation [87]. In the latent phase, the most pronounced alterations involved Groups I and III receptors, with increased Grm5 expression in hippocampal regions and decreased Grm4, Grm7, and Grm8 expression in the hippocampus and temporal cortex, a combination that may favor neuronal hyperexcitability by enhancing excitatory glutamatergic transmission while diminishing presynaptic inhibitory control [87]. In contrast, Group II receptor changes were largely absent early on but emerged in the chronic phase, where increased Grm2 expression in the dorsal hippocampus and reduced Grm3 expression in the temporal cortex suggested delayed compensatory or maladaptive regulatory processes. Although several early alterations normalized by 60 days, reduced Grm8 expression persisted in hippocampal regions, indicating a sustained shift in glutamatergic modulation that may contribute to the maintenance of epileptic network activity [87]. By demonstrating distinct region‐ and phase‐specific expression patterns across mGluR subtypes, this work underscores that glutamatergic modulation in epilepsy is highly dynamic and that effective therapeutic strategies may need to account for both receptor subtype and the temporal progression of epileptogenesis [87].

Complementing these in vivo findings, a neuron–astrocyte computational model (NAG) has been developed to examine how disruptions in extracellular glutamate homeostasis, governed in part by astrocytic mGluRs, drive epileptic neuronal discharges [88]. In this framework, neuronal membrane dynamics, extracellular glutamate concentrations, and ion regulation are integrated to simulate neuron–astrocyte interactions within the microenvironment, allowing the authors to dissect how combined changes in release, uptake, and receptor‐mediated signaling shape network behavior [88]. Extracellular glutamate is modeled as the outcome of three key regulatory pathways: neuronal glutamate release, astrocytic uptake via EAAT2, and astrocytic glutamate binding through mGluRs, together with diffusion processes in the extracellular space, providing a unified description of glutamate turnover [88]. Simulations showed that elevated extracellular glutamate promotes transitions in neuronal firing patterns from resting states to bursting, mixed‐mode spiking, and eventually tonic firing, recapitulating increasing levels of epileptic discharge intensity, and that downregulation of EAAT2 markedly accelerates glutamate accumulation, enhancing excitability and seizure progression [88]. Within this context, astrocytic mGluR–mediated signaling modulated glutamate release through Ca^2+^‐dependent gliotransmission, thereby altering discharge frequency and network stability, while bifurcation analyses indicated that shifts in extracellular glutamate concentration modify the thresholds for state transitions between firing regimes [88]. This theoretical work thus supports the view that astrocytic control of extracellular glutamate, including through mGluR–dependent pathways, is a central determinant of seizure initiation and severity and provides a systems‐level framework to interpret how glial mGluR alterations may contribute to epileptic network dynamics [88].

At the systems and network level in humans, the impact of mGluR dysregulation in epilepsy has been explored using [^11^C]ABP688 PET to map large‐scale mGluR5 networks in patients with focal cortical dysplasia (FCD) and to analyze their topology using graph‐theoretical measures [89]. Moving beyond lesion‐centric views, this study constructed cortical similarity networks based on interregional mGluR5 binding potential across 175 cortical regions, enabling an assessment of global and local properties of mGluR5 organization in both hemispheres [89]. Patients with FCD exhibited significantly reduced global network efficiency and diminished small‐world organization in both the ipsilateral and contralateral hemispheres compared with healthy controls, indicating less integrated and less optimally organized cortical receptor networks [89]. Resilience analyses further showed that mGluR5 networks in FCD were particularly vulnerable to targeted removal of high‐participation hub nodes, pointing to weakened intermodular connectivity that typically supports efficient information transfer across distributed brain regions [89]. Although nodal topology differences did not remain significant after correction, correlation analyses revealed that local efficiency within hub regions of the contralateral hemisphere decreased with increasing epilepsy duration, suggesting that chronic seizure activity is associated with progressive disruption of mGluR5–mediated functional integration in distant cortical areas [89]. These observations support the concept that FCD is associated with widespread alterations in glutamatergic receptor networks, consistent with long‐term plastic reorganization of cortical connectivity driven by persistent epileptic activity and mGluR5 dysregulation [89].

Together, the available evidence indicates that mGluR dysregulation in epilepsy spans tightly coupled mechanisms from receptor–channel interactions in hippocampal neurons to dynamic, stage‐dependent changes in mGluR gene expression, altered astrocyte‐mediated glutamate homeostasis, and disrupted mGluR5 network topology in the epileptic cortex. Current studies, however, are limited by model‐ and region‐specific focus, relatively small sample sizes in clinical imaging work, and the difficulty of directly linking molecular and cellular alterations to long‐term functional and behavioral outcomes in patients. In addition, most preclinical investigations rely on chemically induced or focal models that may not fully capture the heterogeneity of human epilepsies, and cross‐species differences in mGluR subtype expression and signaling may constrain translational generalization. Future work should integrate multimodal approaches that combine receptor subtype–specific pharmacology, longitudinal transcriptomic and imaging analyses, and refined neuron–glia computational models to better connect mGluR signaling dynamics with network‐level plasticity and clinical phenotypes. Such studies, particularly when extended to diverse epilepsy syndromes and coupled with selective modulators of distinct mGluR subtypes, will be essential to identify which components of the mGluR signaling machinery constitute robust, disease stage–specific therapeutic targets for modulating pathological excitability without disrupting physiological synaptic plasticity.

### 10.5. AD

AD provides a clear example of how dysregulated mGluR–dependent signaling and plasticity intersect with core pathogenic processes such as protein synthesis imbalance, receptor loss, and network‐level glutamatergic dysfunction. Building on the mechanistic framework outlined in the plasticity sections, converging evidence at molecular, synaptic, and systems levels indicates that specific mGluR subtypes (notably mGluR5 and mGluR1α) are altered in a manner that both reflects and potentially contributes to impairments in hippocampal LTP/LTD, medial temporal lobe circuit integrity, and cognitive decline in AD. In particular, studies in transgenic models and in patients suggest that translational control downstream of Group I mGluRs is disrupted, that subtype‐specific receptor availability is reduced in vulnerable hippocampal and parahippocampal regions, and that these changes closely track amyloid burden, neurodegeneration markers, and memory performance, underscoring the relevance of mGluR signaling to AD–related synaptic failure.

One line of evidence comes from experimental work in the APP/PS1 transgenic mouse model, which directly links Alzheimer’s pathology to impaired mGluR–dependent LTD through dysregulated translational control at the elongation step. In aged APP/PS1 mice, hippocampal mGluR–LTD at CA3–CA1 synapses is significantly impaired, indicating a deficit in a key synaptic plasticity mechanism that normally contributes to adaptive tuning of excitatory transmission and memory‐related circuit function. This impairment is associated with abnormal phosphorylation of the elongation factor eEF2, a central regulator of mRNA translation whose phosphorylation suppresses protein synthesis, suggesting that pathological changes in AD interfere with the activity‐dependent translational machinery required for mGluR–LTD expression. Genetic suppression of eEF2K, the kinase responsible for eEF2 phosphorylation, restores mGluR–LTD without altering basal synaptic transmission, pointing to a selective involvement of the eEF2K–eEF2 axis in plasticity failure rather than in baseline synaptic integrity. Consistent with this, pharmacological inhibition of eEF2K with NH125 in hippocampal slices from APP/PS1 mice rescues the mGluR–LTD deficit, supporting the idea that reducing eEF2 phosphorylation can normalize mGluR–dependent synaptic plasticity in the AD model. Together, these findings indicate that altered translational regulation downstream of mGluR activation contributes to synaptic plasticity impairments in AD and identify the eEF2K–eEF2 pathway as a potential target for restoring mGluR–LTD and associated cognitive functions [90].

Complementing these mechanistic data, in vivo imaging studies in patients extend the picture from synaptic physiology to whole‐brain markers of disease burden by quantifying subtype‐specific mGluR availability. Using the mGluR5–specific PET radiotracer [18F]PSS232 in conjunction with structural MRI and additional PET ligands, reduced mGluR5 availability has been observed in the hippocampus and parahippocampal gyrus of individuals with AD compared to cognitively normal controls, indicating that mGluR5–mediated glutamatergic signaling is diminished within medial temporal lobe structures that are crucial for episodic memory. Within the AD cohort, regional mGluR5 availability shows a positive association with amyloid deposition measured by [18F]Florbetapir PET, suggesting that receptor changes co‐occur with, and may be influenced by, local amyloid pathology. mGluR5 availability also correlates positively with glucose metabolism assessed by [18F]FDG PET and with gray matter volume, implying that lower receptor availability is linked to reduced neuronal activity and structural atrophy in these regions. In addition, negative associations between mGluR5 availability and plasma neurodegeneration biomarkers such as neurofilament light chain and phosphorylated tau (p‐tau181) indicate that reduced mGluR5 expression tracks with systemic measures of neuronal damage. Importantly, decreased hippocampal mGluR5 availability correlates with poorer performance on global cognitive scales, including MMSE and MoCA‐B, reinforcing the functional significance of these receptor alterations. Overall, these imaging data suggest that mGluR5 availability integrates multiple aspects of Alzheimer’s pathology, amyloid burden, metabolic dysfunction, neurodegeneration, and cognitive impairment, and may serve as a potential biomarker for disease progression [91].

Postmortem analyses further support a role for subtype‐ and region‐specific mGluR changes in AD by demonstrating selective downregulation of mGluR1α in vulnerable hippocampal and cortical territories. Immunohistochemical examination of human brain tissue from Alzheimer’s patients reveals a significant reduction in mGluR1α expression within the CA1 subfield, particularly in the stratum pyramidale and stratum radiatum, as well as in the subiculum and EC, all of which are among the earliest and most severely affected regions in AD. In contrast, mGluR1α expression appears relatively preserved in other hippocampal regions such as CA2, CA3, and the dentate gyrus, indicating that mGluR1α dysregulation is region‐selective rather than uniformly distributed across the hippocampal formation. Quantitative analyses show a marked decrease in receptor density in CA1 pyramidal and radiatum layers, suggesting that modulatory glutamatergic signaling is particularly compromised in circuits that support hippocampal output and memory consolidation. Additional reductions in the subiculum and EC point to broader disruption of medial temporal lobe networks that integrate hippocampal information with neocortical areas. The authors discuss that such downregulation of mGluR1α could increase neuronal vulnerability by weakening mechanisms that normally help constrain NMDAR–mediated excitotoxicity, or might reflect a compensatory attempt to limit excessive glutamatergic drive in diseased tissue. In either case, the data highlight that selective alterations in mGluR1α expression are a feature of Alzheimer’s pathology and underscore the importance of considering receptor subtype and regional context when evaluating glutamatergic targets for therapeutic intervention [92].

Across the AD studies considered in Section 10.5, converging evidence indicates that subtype‐ and region‐specific alterations in mGluR signaling are closely linked to synaptic plasticity impairments, medial temporal lobe circuit dysfunction, and core pathological markers such as amyloid burden, neurodegeneration, and cognitive decline, collectively highlighting mGluR pathways as mechanistically relevant to disease‐related synaptic failure. At the same time, important limitations temper the interpretation and translational generalization of these findings, including the reliance on a single transgenic mouse line for mechanistic dissection of mGluR–LTD, the correlative nature of PET imaging metrics of mGluR5 availability in relatively small and clinically heterogeneous patient cohorts, and the postmortem snapshot provided by mGluR1α immunohistochemistry, which cannot resolve the temporal sequence of receptor alterations relative to plaque and tangle pathology. Future work will benefit from integrating longitudinal in vivo imaging of defined mGluR subtypes with circuit‐level plasticity measurements and cell type–specific manipulations in animal models, as well as from extending these approaches to larger, phenotypically well‐characterized clinical samples to determine whether mGluR–related measures can serve not only as mechanistic readouts but also as robust biomarkers and therapeutic targets for modifying synaptic and cognitive trajectories in AD.

### 10.6. PD

Dysregulation of mGluRs emerges as a convergent mechanism linking synaptic imbalance, circuit‐level dysfunction, and the evolution of motor and nonmotor phenotypes across NDDs. In PD, progressive degeneration of the nigrostriatal dopaminergic pathway is accompanied by maladaptive changes in glutamatergic transmission within basal ganglia circuits, and alterations in specific mGluR subtypes appear to shape both disease progression and treatment‐related complications. Within this framework, studies in postmortem human tissue and experimental models collectively indicate that changes in mGluR distribution and signaling are not epiphenomena, but may participate in compensatory or pathogenic processes that influence motor fluctuations, axonal integrity, and neuropsychiatric manifestations.

In the PD basal ganglia, Group II mGluR2/3 display a regionally differentiated distribution in healthy brains, with the highest binding in the caudate nucleus, lower levels in the putamen, and approximately half that level in the globus pallidus, consistent with their role in modulating glutamatergic inputs within motor circuits [93]. In postmortem samples from PD patients, mGluR2/3 binding is significantly reduced in the caudate nucleus and internal globus pallidus, particularly in individuals who did not develop wearing‐off motor complications, whereas no clear differences emerge between patients with and without dyskinesias. This pattern supports the view that downregulation of mGluR2/3 may represent an adaptive response to heightened glutamatergic drive, potentially stabilizing motor output by dampening excessive glutamate release and partially buffering fluctuations in dopaminergic signaling within basal ganglia loops [93]. The authors, therefore, propose that altered mGluR2/3 responsiveness contributes to the regulation of levodopa‐related motor complications, highlighting these presynaptic receptors as potential pharmacological targets specifically for managing wearing‐off phenomena rather than dyskinesias per se [93].

Complementing these observations in human tissue, experimental work in toxin‐based models points to a distinct but converging role of mGluR5 in early degenerative processes that precede overt neuronal loss. In a 6‐hydroxydopamine (6‐OHDA) model of PD, exposure to the neurotoxin leads to a marked upregulation of mGluR5 expression during axonal degeneration in primary neurons, suggesting that this receptor becomes engaged at an early stage of the neurodegenerative cascade [94]. Pharmacological blockade of mGluR5 with the selective antagonists MPEP and MTEP markedly reduces axonal degeneration in vitro, indicating that mGluR5 signaling contributes to the propagation of axonal injury rather than merely reflecting downstream damage [94]. Mechanistic analysis reveals that 6‐OHDA rapidly elevates intra‐axonal calcium, activates the calcium‐dependent protease calpain, and enhances ERK phosphorylation, all of which are established mediators of neuronal injury; importantly, inhibition of mGluR5 attenuates calcium influx and suppresses calpain activation and ERK phosphorylation, positioning mGluR5 upstream of a calcium‐dependent pathway that drives axonal damage. In vivo, administration of MPEP in 6‐OHDA–lesioned rats attenuates degeneration of dopaminergic axons in the striatum before frank loss of neuronal cell bodies, supporting the relevance of this mechanism to early PD–like pathology and suggesting that mGluR5 antagonism may exert neuroprotective effects at a stage when axonal pathology is still potentially modifiable [94].

Building on these mechanistic insights, pharmacological modulation of mGluR5 has been explored not only for neuroprotection but also for symptomatic treatment of established PD, including both motor and non‐motor domains. Degeneration of the nigrostriatal pathway is known to produce excessive glutamatergic activity in the basal ganglia, which contributes to motor dysfunction and is also implicated in the emergence of psychiatric symptoms such as anxiety, depression, and compulsive behaviors; targeting mGluR5 thus offers a way to dampen pathological glutamatergic signaling across these domains [95]. In rodent models, oral administration of dipraglurant, a selective NAM of mGlu5, produces a dose‐dependent reduction in haloperidol‐induced catalepsy, indicating robust antiparkinsonian activity without evidence of motor suppression [95]. In the same study, dipraglurant exerts beneficial effects on nonmotor symptom–like behaviors by increasing punished licks in the Vogel conflict‐drinking test, reducing immobility in the forced swim test, and decreasing marble‐burying behavior, consistent with anxiolytic, antidepressant, and anticompulsive‐like properties [95]. Notably, these improvements occur without impairment of motor coordination or locomotor activity in Rotarod and locomotion assays, suggesting that behavioral effects are not secondary to sedation or global motor depression [95]. Pharmacokinetic data further demonstrate rapid absorption, central nervous system penetration, and cerebrospinal fluid concentrations that mirror plasma exposure, indicating that systemic dosing can achieve brain levels compatible with receptor engagement [95]. Taken together with the mechanistic evidence from toxin‐lesion models, these findings position mGluR5 as a functionally important node where glutamatergic dysregulation intersects with both motor and psychiatric manifestations of PD, and they support the rationale for mGluR5‐NAMs such as dipraglurant as candidate therapies that may address multiple symptom domains while potentially interacting with underlying degenerative processes [95].

Taken together, these observations indicate that mGluR dysregulation in PD spans alterations in receptor expression, subcellular localization, and signaling, with potentially distinct but converging contributions from Group II and mGluR5–mediated pathways to motor fluctuations, axonal integrity, and neuropsychiatric symptoms. While the available postmortem and experimental data support a role for specific mGluR subtypes in both compensatory and pathogenic processes, most evidence derives from limited sample sizes, toxin‐based animal models, and behavioral paradigms that only partially recapitulate the complexity and chronicity of human PD, which constrains the generalizability of current conclusions. In particular, the temporal sequence of receptor changes relative to dopaminergic degeneration, the cell‐type and circuit specificity of mGluR signaling, and potential interactions with other neurotransmitter systems remain incompletely defined, and clinical translation of mGluR–targeting strategies is still in its early stages. Future studies integrating longitudinal in vivo imaging, cell‐ and circuit‐specific manipulations, and well‐powered clinical trials with subtype‐selective modulators will be essential to clarify whether modulating mGluR2/3 and mGluR5 can not only ameliorate motor and non‐motor symptoms, but also modify disease trajectories. A more systematic mapping of mGluR alterations across disease stages and patient subgroups including those with prominent psychiatric or cognitive features, may ultimately enable stratified, mGluR–based interventions that are tailored to specific patterns of glutamatergic dysregulation rather than applying a uniform approach to all individuals with PD.

### 10.7. HD

HD illustrates how dysregulation of Group I mGluRs, particularly mGluR5, intersects with core pathogenic processes, including autophagy failure, transcriptional abnormalities, synaptic dysfunction, and systemic metabolic disturbances, to shape both neuronal vulnerability and disease progression. In HD, mutant huntingtin (mHTT) aggregates preferentially damage corticostriatal circuits, and converging evidence from genetic, pharmacological, and imaging studies suggests that altered mGluR5 signaling contributes to this vulnerability by engaging intracellular pathways that regulate aggregate clearance, neurotrophic support, gene expression programs, and peripheral inflammatory and metabolic states. By integrating longitudinal receptor imaging with mechanistic work in complementary mouse models, these studies collectively position mGluR5 not only as a modulator of intracellular signaling and synaptic function but also as a candidate biomarker and therapeutic target in HD, while underlining the need to balance receptor inhibition with preservation of its physiological roles.

In the zQ175 mouse model, characterized by progressive accumulation of mHTT aggregates in striatal and cortical neurons, Abd‐Elrahman and Ferguson [96] examined whether pharmacological inhibition of mGluR5 could correct defects in autophagy and neuronal survival pathways. They showed that zQ175 mice exhibit enhanced activation of the PI3K/Akt/mTOR pathway, leading to inhibitory phosphorylation of ULK1, suppression of autophagy, and consequent accumulation of toxic mHTT aggregates. Chronic administration of the mGluR5 NAM CTEP normalized this aberrant signaling profile by reducing phosphorylation of PDK1, Akt, and mTOR, thereby relieving the inhibitory brake on ULK1 and promoting autophagy induction. This restoration of autophagic flux facilitated clearance of mHTT aggregates and was accompanied by increased phosphorylation of CREB and upregulation of cFos and brain‐derived neurotrophic factor (BDNF), which are linked to neuronal survival and differentiation. Consistent with these molecular adaptations, CTEP–treated zQ175 mice displayed reduced apoptotic neuronal loss in the striatum, supporting the view that mGluR5 blockade can coordinate autophagy and neurotrophic signaling in a way that is beneficial in the context of HD–related neurodegeneration [96].

Expanding this mechanistic perspective beyond the CNS, Santos et al. [97] investigated how mGluR5 contributes to the metabolic and inflammatory phenotype in BACHD mice, a transgenic model expressing full‐length mHTT that develops increased body weight, visceral adiposity, and peripheral metabolic disturbances. By crossing BACHD mice with mGluR5 KO animals to generate BACHD/mGluR5^−^/^−^ double mutants, the authors assessed body weight, visceral fat accumulation, adipokine levels, and inflammatory cytokine profiles across different ages. Genetic deletion of mGluR5 reduced body weight gain and decreased visceral adipose tissue in double mutant mice compared with BACHD controls, and it normalized adiponectin while lowering leptin levels, thereby increasing the adiponectin/leptin ratio, a marker of improved metabolic function. In parallel, mGluR5 deletion shifted adipose tissue towards a more anti‐inflammatory state, as indicated by an increased IL‐10/TNF ratio. These findings suggest that mGluR5 signaling influences systemic metabolic homeostasis and inflammatory balance in HD models, implying that targeting mGluR5 could have dual benefits on central and peripheral components of the disease without overextending these observations beyond the studied context [97].

At the receptor level, Bertoglio et al. [98] used in vivo PET with the mGluR5–selective radiotracer ^11^C‐ABP688 to characterize longitudinal changes in receptor availability during HD progression in heterozygous Q175 mice. PET/CT imaging performed at 6, 9, and 13 months revealed a consistent reduction in ^11^C‐ABP688 nondisplaceable binding potential in heterozygous animals relative to wild‐type littermates, with striatal binding decreased by approximately 13%–14% and cortical binding by about 10% across time points. In heterozygous Q175 mice, striatal mGluR5 binding further declined overtime, indicating a progressive reduction in receptor availability as the disease advanced. Postmortem immunohistochemistry confirmed decreased mGluR5 expression in both striatum and cortex, while neuronal density remained preserved, arguing that reduced binding reflected receptor downregulation rather than neuronal loss. These data support the use of mGluR5 PET imaging as a tool to monitor disease‐related receptor changes in vivo and to evaluate interventions that modulate glutamatergic signaling in HD, without inferring direct causal relationships beyond those demonstrated [98].

Linking receptor signaling to transcriptional regulation and synaptic machinery, De Souza et al. [99] explored how mGluR5 activity modulates the transcriptional repressor REST/NRSF via the N‐cadherin/β‐catenin signaling complex in corticostriatal neurons. In primary corticostriatal cultures, pharmacological activation of mGluR5 with the agonist DHPG enhanced Src kinase–dependent phosphorylation of N‐cadherin, disrupting the N‐cadherin/β‐catenin complex and initiating downstream signaling changes. In contrast, inhibition of mGluR5 with CTEP reduced Src activation, stabilized N‐cadherin/β‐catenin interactions, and altered transcriptional pathways. These shifts impacted REST/NRSF expression and its target gene SNAP‐25, a presynaptic protein critical for neurotransmitter release: mGluR5 activation increased REST/NRSF and suppressed SNAP‐25 expression, whereas mGluR5 inhibition lowered REST/NRSF levels and restored SNAP‐25 expression. Validation in zQ175 and BACHD mouse models showed that pharmacological inhibition or genetic deletion of mGluR5 normalized REST/NRSF signaling and SNAP‐25 expression in vivo. Together, these findings indicate that mGluR5 can influence transcriptional programs relevant to synaptic function via Src–mediated modulation of the N‐cadherin/β‐catenin complex, suggesting a route through which mGluR5 dysregulation may contribute to transcriptional and synaptic abnormalities in HD without extending the conclusions beyond the presented evidence [99].

Across these studies, HD emerges as a condition in which mGluR5 dysregulation intersects with autophagy control, neurotrophic support, transcriptional regulation, synaptic function, and systemic metabolic–inflammatory balance, collectively shaping disease‐relevant phenotypes. At the same time, several limitations constrain the interpretation and immediate translational relevance of these findings: evidence is derived predominantly from specific mouse models (zQ175, Q175, and BACHD) and in vitro corticostriatal cultures, receptor modulation has largely been probed through genetic deletion or chronic negative allosteric modulation rather than subtype‐ and cell type–selective approaches, and longitudinal imaging data, while sensitive to receptor downregulation, do not by themselves establish causal relationships with clinical progression. Future work should, therefore, define how mGluR5 signaling varies across distinct neuronal and glial populations within corticostriatal circuits, determine the extent to which modulating mGluR5 can normalize mGluR–dependent synaptic plasticity without impairing physiological signaling, and evaluate whether mGluR5–targeted interventions and PET–based measures of receptor availability translate to measurable benefits and reliable biomarkers in patients with HD.

### 10.8. Amyotrophic Lateral Sclerosis

Alterations in mGluR signaling emerge as a converging mechanism linking glutamate excitotoxicity, neuroinflammation, and nonneuronal cell vulnerability in amyotrophic lateral sclerosis (ALS). In the context of the SOD1G93A model, Group I mGluRs, particularly mGluR5, are not only redistributed at synaptic and extrasynaptic sites but also become critical modulators of microglial and astrocytic responses, thereby coupling aberrant glutamate release to chronic inflammatory and degenerative cascades. Across multiple studies, a coherent picture arises in which disease stage–dependent changes in mGluR1/5 expression and function facilitate excessive glutamatergic transmission, promote excitotoxic damage, and shape microglia–astrocyte–motor neuron cross talk, supporting the view that selective targeting of Group I mGluRs may offer a rational strategy to mitigate ALS progression without overgeneralizing beyond the available experimental evidence.

One line of evidence comes from work examining the physiological and pathological roles of Group I mGluRs expressed by microglial cells and their contribution to neuroinflammation in several neurological disorders, with a particular focus on ALS. Microglia, as resident immune cells of the central nervous system, play a critical role in maintaining neural homeostasis as well as mediating inflammatory responses following injury or disease. This review highlights that microglia express multiple glutamatergic receptors, including ionotropic receptors and mGluRs, which allow these cells to respond to glutamate released during neuronal activity or under pathological conditions. Activation of Group I mGluRs, which are coupled to Gq proteins, stimulates intracellular signaling pathways such as PLC activation, calcium mobilization, and protein kinase C signaling that regulate microglial phenotype and inflammatory responses. Experimental evidence summarized in the review shows that mGluR5 activation can reduce the production of pro‐inflammatory mediators, including tumor necrosis factor‐α, nitric oxide, and reactive oxygen species, thereby limiting microglia‐mediated neurotoxicity. In contrast, under certain pathological conditions, such as intracerebral hemorrhage or severe inflammatory activation, inhibition of mGluR5 may reduce excessive microglial activation and neurodegeneration, indicating that the receptor can exert context‐dependent effects. The review also discusses the involvement of microglial Group I mGluRs in several neurological conditions, including traumatic brain injury, spinal cord injury, epilepsy, AD, ASD, and ALS, emphasizing that modulation of these receptors can influence disease progression by altering neuroinflammatory pathways. Overall, these findings suggest that pharmacological targeting of microglia‐expressed Group I mGluRs represents a potential strategy for regulating neuroinflammation and neurodegeneration in multiple neurological disorders, including ALS, while underscoring that the net outcome of mGluR5 modulation is likely to depend on disease context and activation state [100].

Building on this microglial perspective, in vivo studies in the SOD1G93A mouse model directly examine whether reducing mGluR5 expression can alter ALS disease course, thereby linking receptor dysregulation to functional outcomes. In one such study, Bonifacino et al. [101] generated double‐mutant mice (SOD1G93AGrm5^+/−^) by crossing SOD1G93A transgenic mice with mice carrying a heterozygous deletion of the mGluR5 gene, reducing receptor expression by approximately half. ALS is characterized by progressive degeneration of motor neurons, and excessive glutamate‐mediated excitotoxicity has been proposed as a major contributor to neuronal damage. Behavioral and survival analyses revealed that partial reduction of mGluR5 significantly prolonged lifespan and delayed disease onset in the ALS mouse model, with survival increasing from approximately 134 days in SOD1G93A mice to about 153 days in the double‐mutant animals. Histological analysis of spinal cord tissue demonstrated greater preservation of motor neurons in SOD1G93AGrm5^+/−^ mice compared with SOD1G93A mice, indicating a neuroprotective effect associated with reduced mGluR5 expression. In addition, biochemical studies showed decreased activation of astrocytes and microglia, reduced intracellular calcium levels, and normalization of abnormal glutamate release in spinal cord synaptosomes, suggesting that mGluR5 reduction mitigates excitotoxic and inflammatory mechanisms involved in ALS pathology. Motor performance tests also revealed improvements during disease progression, particularly in male mice, indicating a sex‐dependent component in the functional benefits observed. Taken together, these data demonstrate that partial knockdown of mGluR5 can attenuate several pathological features of ALS in the SOD1G93A mouse model and support the idea that pharmacological inhibition of Group I mGluRs, especially mGluR5, may represent a promising approach for slowing disease progression, while still recognizing that these conclusions are currently restricted to this specific genetic and experimental context [101].

Complementary mechanistic insight into how mGluR1/5 dysregulation emerges during ALS progression comes from studies examining functional changes and expression levels of these receptors in the spinal cord of SOD1G93A mice. ALS is associated with glutamate‐mediated excitotoxicity, and abnormal glutamate release is believed to contribute to motor neuron degeneration. Using spinal cord synaptosomes from mice at presymptomatic (30 and 60 days) and early symptomatic (90 days) stages, researchers evaluated glutamate release induced by the mGluR1/5 agonist 3,5‐dihydroxyphenylglycine (3,5‐DHPG). The results showed that stimulation with 3,5‐DHPG produced similar glutamate release in wild‐type and SOD1G93A mice at 30 and 60 days of age, but a significant increase was observed in 90‐day‐old SOD1G93A mice, indicating that abnormal receptor‐mediated glutamate release emerges during early symptomatic stages of the disease. Further pharmacological experiments demonstrated that both mGluR1 and mGluR5 contribute to this enhanced release and that the mechanism is dependent on intracellular calcium mobilization from endoplasmic reticulum stores through IP_3_–mediated signaling pathways. Confocal microscopy analysis revealed that although overall receptor levels in total synaptosomal preparations did not change significantly, both mGluR1 and mGluR5 were selectively overexpressed in glutamatergic axon terminals of 90‐day‐old SOD1G93A mice. Additional western blot analysis of total spinal cord tissue showed increased expression of these receptors during disease progression, suggesting that non‐presynaptic cellular populations may also contribute to receptor upregulation. Overall, these findings demonstrate that functional alterations and overexpression of Group I mGluRs occur during ALS progression and may contribute to excessive glutamatergic transmission, thereby reinforcing the concept that mGluR1 and mGluR5 represent potential therapeutic targets for modulating excitotoxic mechanisms in ALS, while the precise contribution of each cellular compartment remains to be fully delineated. [102].

A further layer of complexity is added by studies focusing on astrocyte degeneration and its relationship to motor neuron damage in ALS, which position mGluR5 as a key mediator of glial vulnerability. Using the hSOD1G93A transgenic mouse model, investigators analyzed lumbar spinal cord tissue and identified a subpopulation of astrocytes with abnormal spheroid morphology, referred to as spheroid GFAP‐positive cells (SGPCs), appearing predominantly in the ventral horn region near motor neurons. These astrocytes displayed ubiquitin accumulation, altered GFAP cytoskeletal structure, and activation of the apoptotic marker caspase‐3, indicating that they undergo a degenerative process during disease progression. The study further demonstrated that astrocytes expressing mutant SOD1 become highly vulnerable to glutamate‐induced excitotoxicity mediated specifically by mGluR5. Pharmacological experiments showed that activation of mGluR5 reproduced glutamate toxicity, while treatment with the selective mGluR5 antagonist MPEP prevented astrocyte death in vitro. In vivo administration of MPEP to transgenic mice delayed disease onset, reduced astrocyte degeneration, and modestly extended survival, indicating that mGluR5 signaling contributes to astrocyte vulnerability and ALS progression. These findings suggest that excitotoxicity in ALS affects not only motor neurons but also surrounding astrocytes, leading to a reciprocal degenerative interaction that accelerates disease development. Overall, this work highlights a previously underrecognized role of astrocyte degeneration mediated by mGluR5 signaling and suggests that targeting mGluR5 may represent a potential therapeutic strategy for slowing ALS progression, while recognizing that the observed benefits are modest and derived from a single experimental model [103].

Taken together, these ALS studies indicate that Group I mGluR dysregulation contributes to disease mechanisms by enhancing glutamate‐mediated excitotoxicity, shaping microglial and astrocytic inflammatory responses, and promoting glial–motor neuron cross talk that can accelerate degeneration, while simultaneously revealing that mGluR5 signaling may have context‐dependent protective or detrimental effects depending on disease stage and cellular activation state. At the same time, important limitations temper these conclusions, including the predominant reliance on the SOD1G93A mouse model, the focus on early and mid‐symptomatic stages rather than the full disease spectrum, and the use of genetic or pharmacological manipulations that may not fully recapitulate clinically achievable levels of receptor modulation in humans. Moreover, most data derive from preclinical settings and emphasize Group I mGluRs, leaving uncertainties about how these findings generalize to sporadic ALS, to other genetic backgrounds, and to the broader family of mGluR subtypes that may act in compensatory or opposing ways. Future studies will need to clarify cell type–specific and stage‐specific roles of mGluR1 and mGluR5 in ALS, dissect downstream signaling pathways in microglia and astrocytes with higher spatial and temporal resolution, and integrate these mechanistic insights with translational work using selective, brain‐penetrant modulators in diverse ALS models and clinical cohorts to determine whether targeting Group I mGluRs can be safely and effectively leveraged as a disease‐modifying strategy.

### 10.9. Neuropathic Pain

Neuropathic pain emerges when physiological nociceptive signaling becomes persistently dysregulated, and converging evidence implicates mGluRs as key molecular integrators of this maladaptive transition in both central and peripheral pain pathways. Across the pain neuraxis, including dorsal root ganglia, spinal cord, brain stem, and higher‐order cortical regions, region‐ and subtype‐specific alterations in mGluR expression and signaling remodel excitatory–inhibitory balance and synaptic plasticity, thereby sustaining aberrant pain transmission and affective disturbances. In general, Group I mGluRs (mGluR1/5) tend to exert pronociceptive and proinflammatory actions, whereas Groups II and III receptors more often mediate antinociceptive effects by limiting glutamate release and neuronal excitability, providing a mechanistic framework through which mGluR imbalance can drive chronic neuropathic pain and its emotional and cognitive comorbidities in neurological and neurodegenerative conditions.

Within this framework, Chung et al. [104] specifically investigates how upregulation of mGluR5 in the mPFC contributes to the development of neuropathic pain and associated negative mood symptoms following nerve injury. Using a spinal nerve ligation (SNL) rat model of chronic neuropathic pain, the researchers performed PET imaging with the mGluR5–selective radiotracer [^11^C]ABP688 to identify regional changes in receptor expression across the brain. The imaging analysis revealed a significant increase in mGluR5 levels in several pain‐related brain regions, with the most prominent upregulation occurring in the prelimbic region of the mPFC, an area strongly associated with emotional processing and pain perception. Behavioral experiments demonstrated that pharmacological blockade of mGluR5 in this region using the antagonist MPEP significantly reduced mechanical allodynia and alleviated depressive‐like behaviors in neuropathic pain animals, as evidenced by increased paw withdrawal thresholds and reduced immobility time in the forced swimming test. Conversely, lentiviral overexpression of mGluR5 in the same brain region of naïve rats induced pain hypersensitivity, depressive‐like behavior, and anxiety‐like responses even in the absence of nerve injury, and additional behavioral assays confirmed that inhibition of mGluR5 relieved the aversive emotional state associated with chronic pain without altering baseline locomotor activity. Overall, the findings demonstrate that increased mGluR5 signaling in the mPFC plays a causal role in both neuropathic pain and pain‐related mood disturbances, suggesting that targeting prefrontal mGluR5 may represent a promising therapeutic strategy for treating chronic pain and comorbid affective disorders [104].

Complementing this corticolimbic perspective, another study examines the role of persistent mGluR5 activity in the periaqueductal gray (PAG), a central component of the endogenous pain modulatory system that regulates pain transmission through the PAG–rostral ventromedial medulla (RVM) pathway. Using the SNL rat model, the researchers combined behavioral analysis, PET brain imaging, electrophysiology, calcium imaging, and molecular techniques to assess how changes in mGluR5 activity affect neuronal excitability and descending pain control. The findings revealed that mGluR5 in PAG neurons is persistently active under normal conditions, where it maintains neuronal excitability and supports proper pain modulation, and pharmacological activation of PAG mGluR1/5 with the agonist DHPG produced strong analgesic effects and restored abnormal brain activity patterns associated with neuropathic pain, including reduced activity in the PAG–RVM pathway and increased activity in cortical pain‐processing regions. Conversely, inhibition of mGluR5 using inverse agonists such as MPEP induced long‐lasting mechanical allodynia and hypersensitivity even in the absence of nerve injury, demonstrating that loss of persistent mGluR5 activity alone can trigger chronic pain‐like symptoms. Electrophysiological recordings further showed that blocking mGluR5 reduced neuronal excitability and decreased excitatory synaptic transmission within PAG circuits. The study also identified the activity‐dependent protein Homer1a as a key regulator of this process, as increased Homer1a expression in neuropathic pain conditions disrupted persistent mGluR5 activity and weakened excitatory signaling in the PAG–RVM pathway, whereas viral overexpression of Homer1a induced pain hypersensitivity in naïve animals and knockdown of Homer1a prevented chronic pain development following nerve injury. Overall, these data indicate that persistent mGluR5 activity in PAG neurons is essential for maintaining normal pain modulation, and its disruption through Homer1a–mediated signaling contributes to the emergence and maintenance of chronic neuropathic pain [105].

Extending the analysis to brain stem motor and integrative structures, a further study investigates the role of Group I (mGluR1 and mGluR5) in the red nucleus and their contribution to the development of neuropathic pain following peripheral nerve injury. Using a spared nerve injury (SNI) rat model, the researchers examined changes in receptor expression, behavioral pain responses, and inflammatory signaling mechanisms within the red nucleus. Western blot and immunohistochemical analyses demonstrated that both mGluR1 and mGluR5 are constitutively expressed in the red nucleus of normal rats but are significantly upregulated in the region contralateral to nerve injury 2 weeks after SNI, particularly in the magnocellular portion of the red nucleus. Behavioral experiments showed that administration of the mGluR1 antagonist LY367385 or the mGluR5 antagonist MTEP into the red nucleus significantly increased paw withdrawal thresholds and alleviated mechanical allodynia in SNI rats, whereas activation of Group I mGluRs using the agonist DHPG in normal rats induced mechanical hypersensitivity. Molecular analysis further revealed that neuropathic pain was associated with increased expression of the pro‐inflammatory cytokines TNF‐α and IL‐1β in the red nucleus, and pharmacological blockade of mGluR1 or mGluR5 significantly suppressed the elevation of these inflammatory mediators, while activation of Group I mGluRs enhanced TNF‐α and IL‐1β expression, indicating that these receptors promote neuroinflammatory signaling within the red nucleus. Taken together, these findings demonstrate that activation of red nucleus mGluR1 and mGluR5 facilitates neuropathic pain development by stimulating the production of pro‐inflammatory cytokines, further supporting the notion that Group I mGluRs may represent potential therapeutic targets for the treatment of neuropathic pain [106].

Overall, the studies reviewed in this section converge on the view that dysregulated mGluR signaling constitutes a key mechanistic axis linking altered glutamatergic transmission, aberrant synaptic plasticity, and neuroimmune activation to the emergence and maintenance of neuropathic pain and related affective disturbances across the pain neuraxis. By delineating region‐specific changes in mGluR expression and function in structures such as the mPFC, PAG, and red nucleus, these findings highlight how Group I mGluR1/5 signaling can exert both pronociceptive and modulatory roles depending on the cellular context, receptor coupling state, and network position within descending and ascending pain pathways. At the same time, several limitations should be acknowledged, including the predominant reliance on rodent nerve injury models, the focus on relatively short temporal windows after injury, and the use of pharmacological tools and viral manipulations that may have off‐target effects or fail to fully recapitulate the complexity of human neuropathic pain and neurodegenerative conditions. In addition, most studies emphasize Group I receptors, while the contribution of Groups II and III mGluRs, cell type–specific expression patterns (neurons versus glia), and potential interactions with other neuromodulatory systems remain less well defined, underscoring the need for more systematic, comparative analyses across receptor subtypes and disease stages. Future work should, therefore, integrate longitudinal in vivo imaging, cell type–resolved manipulations, and translational clinical studies to clarify how dynamic shifts in mGluR signaling across cortical, subcortical, and spinal circuits shape the trajectory from acute injury to chronic neuropathic pain, and to determine whether selective targeting of specific mGluR subtypes, signaling partners, or network nodes can yield durable analgesic and mood‐stabilizing benefits without disrupting physiological plasticity or inducing intolerable side effects.

### 10.10. Posttraumatic Stress Disorder

In stress‐ and trauma‐related disorders, dysregulation of distinct mGluR subtypes emerges at multiple levels of organization, from receptor anchoring and membrane availability to presynaptic autoregulation of glutamate release and activity‐dependent modulation of synaptic circuits implicated in fear, avoidance, and mood regulation. Human imaging and postmortem data indicate that cortical mGluR5 availability and its synaptic scaffolding environment are altered in PTSD, while preclinical models of stress, depression, and blast‐related mild traumatic brain injury (mTBI) converge on Group II mGluR2/3 signaling as a key determinant of stress resilience versus vulnerability and of the persistence of PTSD–like behavioral traits. Across these studies, aberrant mGluR function is not merely correlative but can be bidirectionally manipulated: enhancing or stabilizing specific receptor populations is associated with pathological fear and avoidance, whereas pharmacological inhibition of Group II receptors can both prevent and reverse stress‐induced behavioral impairments and promote hippocampal neurogenesis, highlighting mGluR subtypes as mechanistically grounded targets for intervention in trauma‐ and stress‐related neuropsychiatric conditions.

Holmes et al. [107] combined in vivo PET imaging with [18F]FPEB and postmortem molecular analyses to examine mGluR5 in medication‐free individuals with PTSD compared with healthy controls, demonstrating significantly higher cortical mGluR5 availability in PTSD, with increases of approximately 19% overall and particularly pronounced elevations in prefrontal regions such as the dorsolateral prefrontal cortex and orbitofrontal cortex. Elevated mGluR5 availability was strongly and positively correlated with avoidance symptoms across multiple cortical regions, suggesting that enhanced receptor signaling within prefrontal circuits may preferentially support specific PTSD symptom dimensions. In parallel, postmortem ventral prefrontal cortex tissue showed significant upregulation of the synaptic scaffolding protein SHANK1, which anchors mGluR5 at the neuronal membrane, together with reduced FKBP5 expression, a regulator of glucocorticoid receptor function, indicating that increased mGluR5 availability in PTSD may arise from enhanced receptor stabilization at excitatory synapses in the context of dysregulated glucocorticoid signaling. These converging imaging and molecular findings support a model in which altered mGluR5 distribution and anchoring at cortical synapses contribute to the neurobiological substrates of PTSD and position mGluR5 as a candidate target for mechanism‐based treatments [107].

Extending the focus from postsynaptic mGluR5 to presynaptic Group II receptors that modulate glutamate release, Highland et al. [108] explored whether manipulating mGlu2/3 activity during stress exposure alters the development of depression‐like behaviors, using both genetic and pharmacological approaches to distinguish subtype‐specific contributions. Mice lacking mGlu2 (Grm2^−/−^), but not those lacking mGlu3 (Grm3^−/−^), showed reduced immobility in the forced swim test and resilience to stress‐induced behavioral deficits across several paradigms, including inescapable shock‐induced escape deficits, corticosterone‐induced behavioral alterations, and chronic social defeat stress‐induced anhedonia, indicating that the absence of mGlu2 confers a stress‐resilient phenotype. Consistent with this, pharmacological blockade of mGlu2/3 with the antagonist LY341495 during stress exposure prevented the development of stress‐induced escape deficits, whereas activation of these receptors with the agonist LY379268 increased susceptibility to maladaptive behaviors, and prophylactic administration of LY341495 up to several days before stress produced long‐lasting protection against both the onset and recurrence of stress‐induced impairments. Microinjection of LY341495 into the mPFC reproduced these protective effects, implicating this cortical region as a critical site where reduced mGlu2 signaling modulates stress‐related synaptic plasticity and behavioral adaptation, and supporting the view that inhibition of mGlu2 enhances stress resilience and may represent a prophylactic strategy for preventing stress‐related disorders such as depression [108].

Building on the role of Group II mGluRs in stress vulnerability, De Gasperi et al. [109] investigated how repetitive low‐level blast exposure shapes the long‐term expression and function of mGluR2 in a rat model that exhibits PTSD–related behavioral traits after mTBI, thereby linking sustained receptor dysregulation to delayed neurobehavioral symptoms that emerge months after injury. Male rats exposed to repeated blast overpressure showed significantly elevated mGluR2 expression in the anterior cortex, hippocampus, and amygdala at long‐term time points (43–52 weeks), while earlier time points (2–6 weeks) did not exhibit such changes, indicating that chronic upregulation of this primarily presynaptic receptor parallels the delayed manifestation of cognitive and fear‐related deficits rather than acute injury responses. RNA analyses confirmed increased mGluR2 gene expression at later stages without corresponding alterations in mGluR3, suggesting a selective and sustained modification of mGluR2 signaling pathways, whereas immunohistochemistry showed that the receptor’s presynaptic distribution remained intact, pointing to quantitative rather than topographical remodeling at excitatory terminals. Importantly, administration of the mGluR2/3 antagonist LY341495 months after blast exposure rapidly reversed deficits in recognition memory and fear learning, demonstrating that persistent mGluR2 upregulation constitutes a modifiable molecular change that contributes to blast‐induced neurobehavioral dysfunction and underscoring Group II receptors as potential therapeutic targets for chronic PTSD–like symptoms following blast‐related injury [109].

In a complementary therapeutic approach within the same blast‐induced mTBI framework, Perez‐Garcia et al. [110] evaluated whether the mGluR2/3 antagonist BCI‐838 can reverse established PTSD–related behavioral traits and enhance hippocampal plasticity, thereby testing if Group II receptor blockade not only normalizes stress‐related behaviors but also engages regenerative mechanisms. Repeated low‐level blast exposure produced persistent anxiety‐like behaviors, exaggerated fear responses, and impaired long‐term recognition memory in rats, modeling chronic PTSD–associated symptomatology after mTBI, and delayed treatment with BCI‐838 at two different doses significantly reversed several of these abnormalities, including anxiety in light–dark emergence, elevated zero maze tests and heightened cued fear conditioning. BCI‐838 also restored long‐term recognition memory and was associated with increased hippocampal neurogenesis in the dentate gyrus, as indicated by elevated BrdU‐ and doublecortin‐labeled neuronal precursor cells, suggesting that antagonism of mGluR2/3 not only alleviates emotional and cognitive symptoms but also promotes structural plasticity in hippocampal circuits implicated in memory and contextual fear processing. Together with the genetic and pharmacological stress studies and the chronic mGluR2 upregulation observed after blast exposure, these findings collectively support a coherent picture in which enhanced Group II mGluR signaling contributes to the maintenance of PTSD–like and depression‐related phenotypes, whereas its inhibition confers stress resilience, reverses established behavioral deficits, and facilitates neurogenic remodeling, thus, reinforcing Group II mGluRs as promising targets for mechanism‐based interventions in trauma‐ and stress‐related neuropsychiatric disorders [110].

Taken together, these human and preclinical findings indicate that dysregulation of distinct mGluR subtypes contributes to stress‐ and trauma‐related phenotypes through partially divergent yet converging mechanisms at the level of cortical and limbic circuitry, synaptic scaffolding, and presynaptic control of glutamate release. However, current evidence is limited by relatively small clinical samples, cross‐sectional imaging designs, species and sex differences across models, and the use of high‐dose or prophylactic pharmacological manipulations that may not directly translate to chronic treatment strategies in patients. In addition, most studies focus on a restricted set of brain regions and behavioral readouts, providing only a partial view of how mGluR–mediated signaling interacts with other neurotransmitter systems, neuroinflammatory processes, and structural network alterations over the course of disease progression. Future work should integrate longitudinal multimodal imaging, circuit‐specific manipulations, and cell type–resolved molecular approaches to map how mGluR5 and Group II mGluR2/3 signaling dynamically shape synaptic plasticity and symptom trajectories in stress‐ and trauma‐related disorders and should extend to carefully designed early‐phase clinical trials testing subtype‐selective modulators with attention to timing of intervention, resilience versus vulnerability phenotypes, and biomarker‐guided patient stratification.

### 10.11. Synthesis, Limitations, and Future Directions

The evidence reviewed in this section identifies mGluR signaling, particularly mGluR5, as a fundamentally context‐dependent regulator of neural function across brain injury and neurological disorders. In acute traumatic and ischemic insults, mGluR5 exerts neuroprotective effects through mechanisms including Arc–mediated stabilization of synaptic signaling and suppression of neuroinflammation. However, during subacute and recovery phases, the same signaling pathways can constrain adaptive circuit reorganization and functional recovery, underscoring a bidirectional and temporally dynamic role. More broadly, dysregulation of mGluR subtypes across neurodevelopmental, neuropsychiatric, and neurodegenerative conditions reflects not uniform alterations in glutamatergic tone, but rather subtype‐, region‐, and cell‐specific changes that reshape synaptic plasticity, neuronal excitability, and neuron–glia interactions. These effects converge on disruptions in mGluR–dependent LTP and depression, linking molecular signaling to circuit dysfunction and behavioral outcomes. Despite these advances, several limitations constrain current understanding. Much of the available evidence derives from animal models and in vitro systems that do not fully capture the cellular complexity, temporal progression, and heterogeneity of human disease. Pharmacological approaches often rely on a limited set of ligands with incomplete subtype specificity, while Groups II and III mGluRs and cell type–specific signaling mechanisms remain comparatively underexplored. In addition, cross‐sectional designs and methodological variability limit mechanistic interpretation, and the temporal windows governing beneficial versus maladaptive mGluR signaling are not well defined. Future research should prioritize integrative, longitudinal strategies that combine receptor‐specific imaging, electrophysiological and transcriptomic profiling, and circuit‐resolved manipulations across disease stages. Particular emphasis should be placed on delineating how mGluR signaling interacts with intracellular pathways such as Gq/PLC, ERK/mTOR, and Arc–dependent synaptic remodeling in a time‐ and cell‐specific manner. Such approaches may enable precision, phase‐specific interventions, for example, transient activation of mGluR5 to mitigate acute excitotoxic and inflammatory damage, followed by targeted inhibition to promote postinjury plasticity and recovery. Advancing toward subtype‐specific and mechanistically grounded modulation of mGluRs will be essential for translating these insights into effective therapeutic strategies.

## 11. Subtype‐Specific Pharmacological Modulation of mGluRs

**Figure 5:**
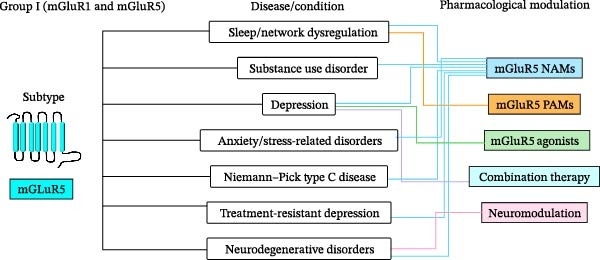
Overview of pharmacological modulation of mGluR5, highlighting negative and positive allosteric modulators (NAMs/PAMs), orthosteric agonists, and emerging approaches including combination therapy and neuromodulation.

**Figure 6:**
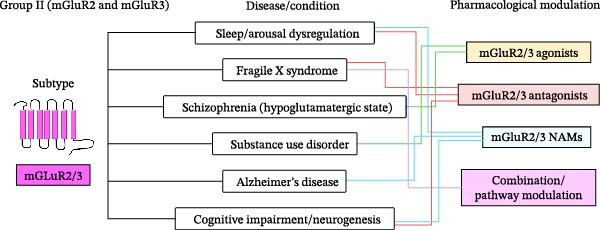
Pharmacological strategies targeting mGluR2/3, including agonists, antagonists, and negative allosteric modulators, along with pathway‐ and combination‐based modulation approaches.

**Figure 7:**
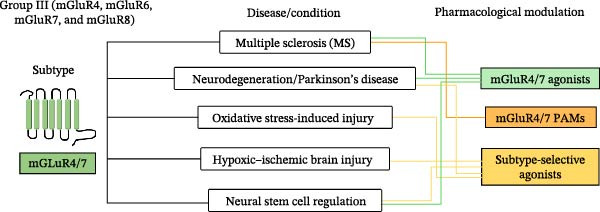
Subtype‐selective modulation of Group III mGluRs (mGluR4/7), illustrating agonists, positive allosteric modulators (PAMs), and subtype‐specific therapeutic targeting strategies.

mGluRs represent a diverse family of G protein–coupled receptors that regulate synaptic transmission, plasticity, and network excitability through subtype‐specific signaling mechanisms. Based on sequence homology, intracellular coupling, and pharmacological profiles, mGluRs are classified into three groups: Group I (mGluR1 and mGluR5), which are primarily postsynaptic and coupled to Gq/PLC signaling; Group II (mGluR2 and mGluR3), and Group III (mGluR4, 6, 7, and 8), which are predominantly Gi/o‐coupled receptors that modulate presynaptic glutamate release and intracellular signaling pathways. Pharmacological targeting of these receptor subtypes, including orthosteric agonists and antagonists, as well as positive allosteric modulator (PAM) and NAM, has revealed distinct and often bidirectional effects on synaptic plasticity, neuronal network dynamics, and disease‐related phenotypes. Importantly, the functional outcomes of mGluR modulation are highly dependent on receptor subtype, cellular localization, and physiological or pathological context. In the following Sections 11.1–11.3), we systematically examine subtype‐specific pharmacological modulation across Group I, II, and III mGluRs. For clarity, key pharmacological agents, their receptor selectivity, mechanisms of action, and functional outcomes are summarized in Table 8. To facilitate a clear understanding of subtype‐specific pharmacological strategies, schematic representations of Group I (mGluR5), Group II (mGluR2/3), and Group III (mGluR4/7) receptor modulation are provided in Figures 5–7, highlighting key classes of ligands, including agonists, antagonists, and allosteric modulators, along with emerging therapeutic approaches such as combination strategies and neuromodulation.

**Table 8:** Subtype‐specific pharmacological modulation of mGluRs.

mGluR group	Subtype	Compounds	Pharmacological type	Mechanism of action	Functional outcome	Key reference
Group I	mGluR5	MPEP, MTEP; ADX47273, LSN2814617	NAM (MPEP, MTEP); PAM (ADX47273, LSN2814617)	Allosteric modulation of mGluR5 (NAMs inhibit signaling; PAMs enhance signaling)	NAMs increased deep sleep and sleep efficiency; enhanced theta and gamma connectivity; reduced alpha activity PAMs increased wakefulness; reduced deep sleep; increased alpha coherence; reduced theta and gamma connectivity	[113]
	mGluR5	MTEP	NAM (antagonist)	mGluR5 regulates glutamate homeostasis (including via the cystine–glutamate exchanger); antagonism reduces glutamate‐overflow‐driven activation of postsynaptic GABAergic medium spiny neurons	Dose‐dependent attenuation of amphetamine self‐administration under FR and PR schedules; environmental condition modulates effects (IC rats show higher motivation at high dose without MTEP; EC shows protection at low dose even without MTEP)	[114]
	mGluR5	CDPPB; MTEP	PAM (CDPPB); NAM (MTEP)	Positive allosteric modulation enhances mGluR5 activity, while antagonism suppresses it; mGluR5 functionally interacts with NMDAR signaling, and reduced mGluR5 activity is required for ketamine’s antidepressant effects	CDPPB attenuates ketamine’s antidepressant effect; MTEP with subeffective ketamine shows synergistic antidepressant effects but abolishes anxiolysis (MTEP anxiolysis seen in EPM, not OFT)	[115]
	mGluR5	CTEP; DHPG; CHPG	NAM (CTEP); agonists (DHPG—nonmembrane permeable, CHPG—membrane permeable)	CTEP inhibits mGluR5 activity and reduces aberrant signaling; DHPG preferentially activates surface mGluR5, while CHPG accesses intracellular mGluR5 pools	CTEP reduces enhanced mGluR–LTD and behavioral abnormalities but not hippocampal memory deficits; in NPC, DHPG (surface mGluR5) LTD is reduced, while CHPG (intracellular) LTD is enhanced	[116]
	mGluR5	MPEP; riluzole	Antagonist (MPEP); glutamate release inhibitor (riluzole)	MPEP blocks mGluR5 signaling; riluzole inhibits glutamate release without directly targeting receptors	MPEP increases corticosterone under stress, raises adrenal weight, and decreases mGluR5 expression, with no effect on stress‐induced catecholamine release; riluzole shows no significant effects on hormones or mGluR5 expression	[117]
	mGluR5	MPEP	Antagonist	Blocks mGluR5 receptor signaling	Dose‐dependent decrease in distress vocalizations during the first 5 min (anxiolytic/anti‐panic effect); no increase in DVocs during the behavioral despair phase (no antidepressant‐like effect)	[118]
	mGluR5	CHPG	Agonist	Selective activation of mGluR5 receptors	In control: reduces fEPSP slope (mGluR5–dependent synaptic depression); after HFMS: CHPG enhances fEPSP slope instead of reducing it, indicating inhibition/impaired mGluR5–dependent depression	[119]
Group II	mGluR2	LY341495; Ro‐4491533	Antagonist (LY341495); NAM (Ro‐4491533)	Inhibition of presynaptic mGluR2 signaling → modulation of glutamatergic neurotransmission and sleep–wake regulatory circuits	Robust cortical arousal with increased wakefulness, enhanced theta/gamma oscillations, reduced deep sleep and REM sleep, increased transitions from sleep to waking, and delayed compensatory sleep without rebound hypersomnolence	[120]
	mGluR2/3	LY341495	Antagonist	Selective antagonism of Group II mGluRs (Gi‐coupled; pre‐ and postsynaptic) modulates mGluR–dependent synaptic signaling (proposed; not directly shown to increase cAMP/PKA)	Abrogates exaggerated mGluR–dependent LTD in Fmr1 KO mice, restoring LTD toward WT levels; enhances LTD in WT mice	[121]
	mGluR2/3	LY354740	Agonist	Activation of Group II mGluRs leads to PKC–dependent phosphorylation of the GluN1 subunit of NMDARs, enhancing NMDAR currents and priming function (metaplasticity; requires concurrent activity for LTP)	Promotes induction of LTP at Schaffer collateral–CA1 synapses by lowering the threshold for NMDAR–dependent plasticity; enhances subsequent HFS–induced LTP	[122]
	mGluR2/3	LY379268	Agonist	Activation of presynaptic Gi/o‐coupled mGluR2/3 (canonical mechanism; downstream effects such as inhibition of cAMP and glutamate release not directly measured in this study)	Reduces motivation for amphetamine self‐administration in a rearing‐dependent manner: low dose effective in EC, high dose in SC, no effect in IC; indicates environment‐dependent modulation of mGluR2/3 function	[123]
	mGluR2/3	BCI‐838 (prodrug → BCI‐632)	Prodrug; active metabolite BCI‐632 is a negative allosteric modulator	Inhibition of mGluR2/3 signaling is associated with transcriptomic upregulation of neurogenic and plasticity‐related pathways (e.g., BDNF, PI3K–mTOR signaling) in the dentate gyrus (causal pathway not directly established)	Enhanced adult hippocampal neurogenesis (↑ DCX, BrdU); improved recognition memory; mimics effects of physical exercise in APP/PS1 Alzheimer’s model	[124]
Group III	mGluR4 (also includes mGluR6/7/8 family)	L‐AP4; PHCCC	Orthosteric agonist (L‐AP4); positive allosteric modulator (PHCCC)	Activation of mGluR4 (Gi/o‐coupled) in dendritic cells reduces cAMP signaling, shifts cytokines (↓ IL‐6, IL‐23; ↑ IL‐12, IL‐27, TGF‐β), and biases CD4^+^ T cells from TH17 toward Treg	Reduced neuroinflammation and EAE severity; increased Treg (Foxp3^+^) cells; decreased TH17–mediated immunity; improved disease recovery	[125]
	mGluR4/6/7/8 (functional dominance: mGluR7)	L‐AP4; AMN082; VU0155041; (S)‐3,4‐DCPG; CPPG	L‐AP4: orthosteric agonist; AMN082: mGluR7–selective agonist; VU0155041: mGluR4 PAM (did not reproduce antiproliferative effects); (S)‐3,4‐DCPG: mGluR8 agonist; CPPG: antagonist	Group III mGluR activation inhibits forskolin‐stimulated cAMP (Gi/o), but effects in hNSPCs are mGluR7‐specific (AMN082, not mGluR4); reduces proliferation and shifts differentiation (↓ neuronal β‐III tubulin, TH; ↓ oligodendrocyte GalC; ↑ astrocytes)	Decreased proliferation without toxicity (AMN082–dependent); reduced neuronal and oligodendrocyte differentiation; increased astrocyte differentiation (mGluR7–dependent)	[126]
	mGluR4	VU0155041; MSOP	VU0155041: selective mGluR4 agonist; MSOP: Group III antagonist	Activation of mGluR4 attenuates H_2_O_2_–induced oxidative stress signaling → inhibition of JNK and p38 MAPK phosphorylation → suppression of apoptotic cascades (↓ procaspase‐8/9/3 cleavage, restoration of Bcl‐2/Bax balance)	Increased NSC viability; reduced apoptosis (↓ TUNEL, ↓ PI‐positive cells); protection against oxidative stress–induced cell death	[127]
	mGluR4	VU0155041	mGluR4–selective agonist	Activation of mGluR4 reduces cAMP levels and inhibits PKA activity, leading to suppression of ASK1 activation and downstream p38 MAPK phosphorylation → attenuation of apoptotic signaling (↓ caspase‐8/9/3 cleavage; ↑ Bcl‐2/Bax ratio)	Attenuation of OGD–induced apoptosis in hNSCs (↓ TUNEL, ↓ apoptosis rate); increased viability; neuroprotection under hypoxic–ischemic conditions	[128]

### 11.1. Group I mGluRs

Subtype‐specific pharmacological modulation of Group I mGluRs, and particularly mGluR5, exerts diverse and often context‐dependent effects on synaptic plasticity, network dynamics, and behavior, reflecting the receptor’s widespread distribution and distinct coupling to intracellular pathways across brain regions and peripheral tissues. Across preclinical models, NAMs, PAMs, orthosteric agonists, and antagonists have been used to dissect how mGluR5 contributes to sleep–wake regulation, reward‐related plasticity, affective and stress‐related behaviors, intracellular versus surface receptor signaling, and neuroendocrine responses, thereby providing mechanistic insight into how this subtype may be leveraged or constrained in translational strategies for neuropsychiatric and NDDs.

Ahnaou et al. [113] investigated how bidirectional allosteric modulation of mGluR5 regulates large‐scale neuronal network dynamics and sleep‐dependent processes linked to synaptic plasticity. Mechanistically, the study focused on how altering mGluR5 signaling influences cortical oscillatory coherence and vigilance‐state organization, which are key electrophysiological correlates of synaptic integration and plasticity, using both NAMs (MPEP and MTEP) and PAMs (ADX47273 and LSN2814617) that selectively target the mGluR5 subtype, a Group I mGluR predominantly localized postsynaptically in cortico‐hippocampal circuits and functionally coupled to NMDAR signaling. Functionally, mGluR5 blockade via MPEP and MTEP enhanced deep sleep consolidation, increased sleep efficiency, and strengthened cortical network coherence, particularly in theta and gamma frequency bands associated with memory processing and synaptic plasticity, whereas positive allosteric modulation (ADX47273 and LSN2814617) promoted wakefulness, reduced deep and REM sleep, and shifted network activity toward increased alpha oscillatory coherence with reduced theta–gamma synchronization, indicating altered functional connectivity. These effects were dose‐dependent and correlated with receptor occupancy, with LSN2814617 exhibiting stronger and longer‐lasting effects than ADX47273 due to higher brain exposure and mGluR5 engagement, suggesting that mGluR5 negative modulation may support cognitive processes by enhancing sleep‐dependent network synchronization, while positive modulation may differentially influence arousal and cortical processing in conditions such as schizophrenia and AD where network dysregulation and sleep disturbances are prominent; however, the study does not directly assess behavioral cognition and confines its conclusions to electrophysiological evidence linking subtype‐specific mGluR5 modulation to mechanisms underlying cognitive and NDDs [113].

Extending this subtype‐specific perspective from large‐scale oscillatory regulation to reward‐related synaptic plasticity, Arndt et al. [114] examined how modulation of glutamate homeostasis influences psychostimulant reinforcement, focusing on the role of mGluR5 in regulating mesocorticolimbic circuitry under different environmental conditions. The study is based on the role of mGluR5 in maintaining synaptic glutamate balance within the nucleus accumbens (NAc) and mPFC, where disruptions in glutamate homeostasis are implicated in addictive behaviors, and used the selective mGluR5 antagonist MTEP (0.3–5.0 mg/kg) to target this Group I receptor, which is predominantly expressed postsynaptically on GABAergic medium spiny neurons in the NAc and modulates responses to glutamate overflow and synaptic plasticity mechanisms linked to reward processing. MTEP produced a dose‐dependent attenuation of amphetamine self‐administration (AMP SA) under both fixed‐ratio (FR‐1) and progressive‐ratio (PR) schedules, with higher doses significantly reducing active lever pressing and, thus, decreasing reward sensitivity and motivation to obtain amphetamine, and these effects were modulated by environmental conditions, as isolated rats showed greater baseline drug‐seeking behavior and differential sensitivity to MTEP compared with enriched rats, indicating that environmental modulation of glutamatergic tone influences mGluR5 function. Locomotor control experiments confirmed that reduced self‐administration was not due to nonspecific motor suppression, and from a disease relevance perspective, the findings indicate that mGluR5 antagonism can reduce psychostimulant reinforcement by modulating glutamate‐dependent reward circuitry, supporting a potential role for this subtype in substance use disorders while appropriately limiting interpretation to behavioral and neuropharmacological outcomes without direct assessment of long‐term addiction phenotypes or cognitive endpoints [114].

In affective and stress‐related domains where mGluR5 is tightly coupled to NMDAR–dependent signaling, Gokalp and Unal [115] investigated the role of this receptor in mediating the behavioral effects of ketamine, focusing on how modulation of mGluR5 influences antidepressant, anxiolytic, and fear‐related outcomes. The study builds on the interaction between mGluR5 and NMDAR–dependent signaling, whereby ketamine‐induced NMDAR blockade leads to increased glutamate release and downstream modulation of postsynaptic receptors, including mGluR5, which is functionally coupled to NMDARs via Homer–Shank scaffolding complexes, and used both the positive allosteric modulator CDPPB (10 mg/kg) and the selective antagonist MTEP (1.25 mg/kg) in combination with either an antidepressant or sub‐effective dose of ketamine to interrogate subtype‐specific contributions. Activation of mGluR5 via CDPPB attenuated the antidepressant effect of ketamine in the forced swim test, as immobility in the CDPPB+ ketamine group did not differ from controls, indicating that enhanced mGluR5 signaling counteracts ketamine’s antidepressant mechanism, whereas inhibition of mGluR5 using MTEP in combination with low‐dose ketamine produced a significant reduction in immobility, demonstrating a synergistic antidepressant effect not observed with either treatment alone; however, this combinatorial treatment abolished the anxiolytic effects seen with individual administration of MTEP or low‐dose ketamine in the elevated plus maze, indicating a functional dissociation between antidepressant and anxiolytic outcomes. Additional behavioral analyses confirmed that these changes were not attributable to general locomotor alterations, and from a disease relevance perspective, the findings suggest that suppression of mGluR5 activity is a critical component of ketamine’s antidepressant action and that subtype‐specific antagonism of mGluR5 may enhance therapeutic efficacy in depressive disorders, while also indicating that such modulation may produce domain‐specific behavioral trade‐offs, highlighting the complexity of targeting mGluR5 in neuropsychiatric conditions [115].

A complementary line of evidence comes from Niemann–Pick Type C (NPC) disease, in which disrupted cholesterol trafficking profoundly alters mGluR5 localization and signaling and thereby reshapes synaptic plasticity and behavior. Toledano‐Zaragoza et al. [116] investigated how NPC1 deficiency, which causes endolysosomal cholesterol accumulation, impairs receptor trafficking and degradation, resulting in aberrant intracellular accumulation of mGluR5 and reduced receptor presence at the plasma membrane, yet leaving this intracellularly retained receptor pool functionally active. In hippocampal slices from NPC1‐deficient mice, this redistribution led to enhanced mGluR5–dependent signaling, increased basal protein synthesis, and exaggerated mGluR–LTD, with enhanced LTD when stimulated with membrane‐permeable agonists (CHPG), consistent with increased intracellular receptor activity, and reduced responses to non‐permeable agonists (DHPG) due to diminished surface receptor availability. Pharmacological intervention with the brain‐penetrant mGluR5 NAM CTEP, administered orally, normalized exaggerated mGluR–LTD and significantly improved multiple behavioral abnormalities, including anxiety‐like behavior, depressive‐like states, and repetitive behaviors, although memory deficits were not rescued. From a disease relevance perspective, these findings demonstrate that intracellular overactivation of mGluR5 is a key contributor to psychiatric manifestations in NPC and that selective inhibition of this receptor can ameliorate behavioral phenotypes, while also refining the understanding of mGluR5 signaling by showing that intracellular receptor pools can drive pathological plasticity independently of surface expression and highlighting subtype‐specific and compartment‐specific targeting as a potential therapeutic strategy in lysosomal storage and NDDs [116].

Beyond central synapses, Pokusa et al. [117] extended the scope of subtype‐specific mGluR5 modulation to peripheral neuroendocrine regulation by examining its involvement in stress‐induced hormone release and gene expression within the adrenal gland. This study focused on how mGluR5 signaling may modulate hypothalamic–pituitary–adrenocortical (HPA) axis activity through potential regulation of steroidogenesis and catecholamine secretion under stress conditions and used the selective mGluR5 antagonist MPEP (1 mg/kg, repeated administration) alongside the glutamate release inhibitor riluzole to assess upstream glutamatergic influence. MPEP treatment revealed that mGluR5 is expressed in both the adrenal cortex and medulla, indicating peripheral localization beyond central synaptic compartments, and pharmacological blockade resulted in a selective increase in corticosterone levels during restraint stress without affecting catecholamine release, indicating a differential role of mGluR5 in steroid versus catecholamine output. In parallel, MPEP reduced mGluR5 gene expression and increased adrenal gland weight without significantly altering key steroidogenic regulators (StAR and SF‐1), and riluzole failed to modify hormone levels or receptor expression, suggesting limited contribution of endogenous glutamate release to adrenal mGluR5 regulation under basal conditions; from a disease relevance standpoint, these findings indicate that mGluR5 exerts a modest and selective modulatory role in HPA axis activation during stress, particularly influencing glucocorticoid release without significantly regulating catecholaminergic stress responses, and the study appropriately limits conclusions regarding broader impacts on stress‐related or neuropsychiatric disorders [117].

Further integrating subtype specificity across Groups I, II, and III receptors in a stress‐related behavioral model, White et al. [118] evaluated modulation of glutamatergic receptors in a chick social‐separation stress paradigm designed to model both panic‐like anxiety and treatment‐resistant depression. The study is based on differential roles of mGluRs in regulating glutamatergic transmission across distinct phases of stress, with Group I mGluRs (including mGluR5) enhancing postsynaptic excitatory signaling and Groups II/III receptors primarily acting presynaptically to inhibit glutamate release and employed the mGluR5 antagonist MPEP, the mGluR2/3 agonist LY404039, and the mGluR7 agonist AMN082 to allow subtype‐specific functional assessment. MPEP (3–30 mg/kg) significantly reduced distress vocalizations during the initial 5‐min isolation phase, indicating a robust anxiolytic (antipanic) effect, but had no effect during the later depression‐like phase, suggesting limited antidepressant efficacy in this treatment‐resistant depression model, whereas LY404039 similarly reduced early‐phase vocalizations without influencing later depressive‐like behavior, and the mGluR7 agonist AMN082 selectively increased vocalizations during the later phase, indicative of antidepressant‐like effects, without affecting the anxiety phase. These phase‐specific outcomes highlight a functional dissociation between receptor subtypes in regulating anxiety versus depressive behaviors within the same model, and from a disease relevance perspective, the findings suggest that mGluR5 antagonism primarily modulates panic‐related behaviors, whereas mGluR7 activation may be more relevant for antidepressant effects in treatment‐resistant conditions, with Group II receptors contributing mainly to anxiolytic regulation; importantly, the study emphasizes translational limitations, and therefore, confines its conclusions to preclinical screening utility rather than direct therapeutic efficacy [118].

Finally, Holl et al. [119] explored how neuromodulatory interventions intersect with mGluR5–dependent synaptic plasticity by investigating how prior high‐frequency magnetic stimulation (HFMS) modulates mGluR–dependent plasticity at SC–CA1 synapses, focusing on metaplastic interactions between physical stimulation and receptor‐mediated depression. The study is grounded in activity‐dependent modulation of glutamatergic signaling, where HFMS induces synaptic potentiation through NMDAR–dependent processes and alters subsequent responsiveness of metabotropic pathways, potentially via changes in intracellular signaling or receptor coupling, and used the selective mGluR5 agonist CHPG (150 µM) and the Group I mGluR agonist DHPG (100 µM) to differentiate between mGluR5–specific and combined mGluR1/5–mediated effects. In control slices, CHPG induced a reduction in fEPSP slopes, reflecting mGluR5–dependent synaptic depression, whereas in HFMS–treated slices, CHPG instead enhanced synaptic responses, indicating a reversal or inhibition of mGluR5–mediated depression. While DHPG produced robust synaptic depression in both control and HFMS–treated slices, demonstrating that combined mGluR1/5–mediated depression remained intact despite prior stimulation. This selective disruption suggests that HFMS impairs mGluR5–specific signaling while sparing broader Group I mGluR function, and from a disease relevance perspective, the findings indicate that external neuromodulatory interventions such as magnetic stimulation can selectively alter mGluR5–dependent plasticity mechanisms, which may be relevant for therapeutic approaches like repetitive transcranial magnetic stimulation in major depressive disorder where altered mGluR5 signaling has been implicated, while appropriately limiting conclusions to acute synaptic effects without direct behavioral or clinical validation [119].

Across these studies, subtype‐specific pharmacological modulation of Group I mGluRs, and particularly mGluR5, emerges as a powerful but highly context‐dependent lever on synaptic plasticity, network oscillations, affective and stress‐related behaviors, reward processing, and even peripheral endocrine function, yet important limitations temper immediate translational extrapolation. While NAMs, PAMs, orthosteric agonists, and antagonists consistently demonstrate that mGluR5 can bidirectionally regulate sleep–wake organization, theta–gamma coupling, chemically induced LTD, psychostimulant reinforcement, ketamine‐related antidepressant responses, and stress‐induced hormone release, these findings are largely derived from acute or subchronic preclinical paradigms in rodents or other non‐human species, often without direct behavioral readouts of cognition or long‐term disease modification, and with limited coverage of dose ranges, sex, age, and comorbidity profiles. Section 11.1 also highlights that many experiments isolate single ligands and end points in highly controlled settings, which constrains conclusions about how mGluR5 modulation would behave in the complex milieu of human neurodegenerative or neuropsychiatric disorders where multiple receptor systems, intracellular pools (surface versus intracellular mGluR5), and cell types (neurons versus glia versus peripheral endocrine cells) interact dynamically. Clinical and translational work further indicates that despite strong mechanistic and behavioral rationale, mGluR5 NAMs and PAMs have faced challenges related to safety, tolerability, and efficacy in humans, including neuropsychiatric adverse events and context‐dependent effects that may depend on disease stage or pathological substrates such as amyloid, underscoring that both hypo‐ and hyperactivation of mGluR5 can be detrimental depending on brain region and pathology. Future research will, therefore, need to refine pharmacological selectivity not only at the level of receptor subtype but also at the level of signaling bias (for example, favoring specific intracellular cascades), subcellular compartment (surface versus intracellular mGluR5), and circuit or cell‐type specificity, potentially through targeted delivery, biased allosteric ligands, or combination approaches with neuromodulatory interventions such as magnetic stimulation. Longitudinal studies that explicitly integrate electrophysiological markers of LTP/LTD, network oscillations, and behavior in disease‐relevant models, followed by carefully designed early‐phase clinical trials with robust safety monitoring, will be essential to determine whether the mechanistic plasticity effects documented here can be harnessed for durable therapeutic benefit in neurodegenerative and stress‐related brain disorders while minimizing off‐target and regionally inappropriate consequences.

### 11.2. Group II mGluRs

Subtype‐specific pharmacological modulation of Group II mGluRs (mGluR2/3) illustrates how a single receptor class can exert divergent effects on synaptic plasticity, network excitability, and disease‐related phenotypes, depending on brain region, cellular localization, and the direction of receptor modulation (agonism versus antagonism). Across models of sleep–wake regulation, FXS, schizophrenia‐related hypoglutamatergic states, addiction, and AD, both orthosteric ligands and allosteric modulators targeting mGluR2/3 have been used to either dampen or enhance glutamatergic signaling, thereby normalizing aberrant LTP/LTD, rebalancing cortical arousal, or promoting neurogenesis. In this context, presynaptic mGluR2/3 generally function as Gi/o‐coupled autoreceptors that inhibit adenylyl cyclase, reduce cAMP–PKA signaling, and suppress glutamate release, while postsynaptic and glial mGluR3 can additionally modulate NMDAR–dependent plasticity and neurotrophic support, placing Group II receptors at a critical interface between synaptic physiology and neurodegenerative or neuropsychiatric processes. The following studies collectively highlight how subtype‐selective antagonism, agonism, or negative allosteric modulation of mGluR2/3 can bidirectionally tune synaptic and network function, with convergent implications for cognitive impairment, mood disorders, substance use disorders, and AD, while remaining within the boundaries of preclinical and mechanistic evidence.

Ahnaou et al. [120] examined the role of mGluR2 signaling in regulating cortical arousal and sleep–wake transitions using electrophysiological and pharmacological approaches in rodents. Mechanistically, the study focused on presynaptic mGluR2–mediated modulation of glutamate release, which influences network excitability and vigilance‐state stability, particularly through its role in regulating transitions between sleep and wakefulness. Pharmacological intervention involved both an orthosteric antagonist (LY341495) and a NAM (Ro‐4491533), enabling selective inhibition of receptor signaling. These compounds target the mGluR2 subtype, a Group II metabotropic receptor predominantly localized presynaptically in limbic and cortical circuits, where it functions as an autoreceptor controlling glutamatergic transmission. Functionally, mGluR2 blockade induced a robust and sustained arousal phenotype characterized by increased active wakefulness, reduced deep and REM sleep, prolonged sleep onset latency, and enhanced transitions from light sleep to waking states, as shown in both rats and wild‐type mice. At the network level, this arousal state was associated with increased theta and gamma oscillatory power and reduced slow‐wave activity, indicating a shift toward higher‐frequency cortical activation. Importantly, the absence of these effects in mGluR2 KO mice confirmed receptor‐specific mediation of the observed responses. From a disease relevance perspective, these findings suggest that mGluR2 inhibition enhances cortical activation and vigilance‐related network dynamics, which may be beneficial in conditions characterized by cognitive impairment or hypoarousal (e.g., depression or schizophrenia), although the concomitant reduction in slow‐wave activity indicates potential trade‐offs for sleep‐dependent memory consolidation. The study remains cautious in interpretation, as behavioral cognition was not directly assessed, but provides strong mechanistic and electrophysiological evidence for subtype‐specific modulation of mGluR2 in regulating brain state transitions [120].

Building on the concept that altered mGluR signaling can drive pathological synaptic plasticity, Choi et al. [121] investigated the role of mGluR signaling in FXS, focusing on how dysregulated mGluR–dependent plasticity can be pharmacologically corrected in the Fmr1 KO mouse model. Mechanistically, the study is based on the “mGluR theory of Fragile X,” where loss of FMRP leads to exaggerated mGluR–dependent LTD at SC–CA1 synapses, driven by dysregulated protein synthesis downstream of glutamatergic signaling. Pharmacological intervention involved chronic administration of lithium and the Group II mGluR antagonist LY341495, allowing assessment of both downstream pathway inhibition and receptor‐level modulation. LY341495 selectively targets Group II mGluRs (mGluR2/3), which are primarily presynaptic Gi‐coupled receptors that regulate glutamate release and intracellular cAMP signaling. Functionally, Fmr1 KO mice exhibited significantly enhanced DHPG–induced LTD (~62%–68% of baseline versus ~81%–83% in wild‐type), confirming exaggerated synaptic depression. Chronic treatment with either lithium or LY341495 restored LTD to near wild‐type levels (~79%–84%), effectively normalizing synaptic plasticity without altering basal synaptic transmission or paired‐pulse facilitation. Importantly, these effects were observed with both long‐term treatments initiated during development and shorter‐term treatment initiated in adulthood, indicating that pharmacological correction of mGluR–dependent plasticity is not restricted to early developmental windows. From a disease relevance perspective, these findings demonstrate that targeting Group II mGluRs or their downstream signaling pathways can reverse core synaptic plasticity abnormalities in FXS, supporting their therapeutic potential. However, the study appropriately limits interpretation to electrophysiological rescue of LTD and does not directly assess higher‐order cognitive or behavioral outcomes, although it aligns with prior evidence suggesting translational relevance [121].

The capacity of Group II mGluRs to regulate plasticity extends beyond LTD to LTP metaplasticity. Rosenberg et al. [122] investigated how activation of Group II mGluRs modulates synaptic plasticity through a metaplastic mechanism that primes NMDAR–dependent LTP at SC–CA1 synapses. Mechanistically, the study demonstrates that activation of mGluR2/3 engages a noncanonical signaling pathway involving PKC activation and intracellular calcium release, leading to phosphorylation of the GluN1 subunit of NMDARs at Ser896, thereby enhancing NMDAR function. Pharmacological manipulation was performed using the selective Group II mGluR agonist LY354740, alongside antagonists such as LY341495 and multiple pathway inhibitors (e.g., PKC blockers and NMDAR antagonist D‐AP5) to dissect signaling mechanisms. These compounds target the mGluR2 and mGluR3 subtypes, which are expressed both pre‐ and postsynaptically, with evidence from paired‐pulse ratio analysis indicating that LTP in this context is primarily mediated by postsynaptic receptors. Functionally, activation of mGluR2/3 induced a significant potentiation of synaptic responses (~137% of baseline), which was abolished by receptor antagonism or blockade of PKC and NMDARs, confirming a requirement for both signaling pathways. Importantly, mGluR2/3 activation did not directly induce classical LTP in isolation but instead lowered the threshold for subsequent LTP induction, as prior activation enhanced high‐frequency stimulation‐induced LTP. Subtype‐specific analysis using KO models further revealed that mGluR3 plays a dominant role, as LTP was significantly reduced in mGluR3–deficient mice but preserved in mGluR2 KOs, consistent with its broader synaptic and extrasynaptic distribution and ability to modulate both GluN2A– and GluN2B–containing NMDARs. From a disease relevance perspective, these findings suggest that Group II mGluRs, particularly mGluR3, regulate synaptic plasticity by priming NMDAR–dependent LTP, providing a mechanistic basis for their proposed role in conditions associated with hypoglutamatergic states, such as schizophrenia. However, the study appropriately confines conclusions to synaptic and electrophysiological outcomes without direct behavioral validation [122].

Consistent with this regulatory role in glutamatergic drive and motivational circuits, Garcia and Cain [123] investigated how activation of presynaptic Group II mGluRs modulates glutamatergic transmission underlying psychostimulant reinforcement, with a particular focus on the influence of environmental context on receptor function. Mechanistically, the study is based on mGluR2/3–mediated Gi/o‐coupled inhibition of cAMP signaling, which suppresses presynaptic glutamate release in mesocorticolimbic circuits, particularly within the nucleus accumbens, thereby regulating excitatory drive associated with drug‐seeking behavior. Pharmacological manipulation was performed using the highly selective mGluR2/3 agonist LY379268 (0.3 and 1 mg/kg). This compound targets the mGluR2 and mGluR3 subtypes, predominantly localized presynaptically, where they function as autoreceptors controlling glutamate release. Functionally, LY379268 produced dose‐dependent suppression of AMP SA under a PR schedule, reflected by reductions in active lever pressing and drug infusions, particularly in standard condition rats at the higher dose and in enriched condition (EC) rats at the lower dose. However, this suppressive effect was absent in isolated condition (IC) rats, where neither dose significantly reduced AMP intake or motivation, indicating impaired mGluR2/3 functional responsiveness. Notably, EC rats exhibited lower baseline motivation (reduced breakpoints) compared to IC and SC rats, suggesting that environmental enrichment modifies glutamatergic tone and receptor efficacy rather than absolute drug intake. Importantly, normalization analyses demonstrated that common statistical transformations can obscure these subtype‐specific pharmacological effects by inflating variability and masking group differences. From a disease relevance perspective, these findings indicate that activation of mGluR2/3 can suppress psychostimulant reinforcement through presynaptic control of glutamate release, but that the efficacy of this modulation is highly dependent on the functional state of the receptor shaped by environmental and developmental factors, which may have implications for variability in therapeutic responses in substance use disorders. The study appropriately limits interpretation to behavioral and neuropharmacological outcomes without extending to long‐term addiction phenotypes [123].

In neurodegenerative contexts, the same receptors can be engaged in the opposite pharmacological direction to promote structural plasticity. Garcia et al. [124] investigated how modulation of Group II mGluRs influences adult hippocampal neurogenesis (AHN) and cognitive function in an AD amyloidosis model (APP/PS1 mice). Mechanistically, the study is based on mGluR2/3–mediated regulation of glutamatergic signaling and downstream neurotrophic pathways, where inhibition of these Gi/o‐coupled receptors is proposed to enhance synaptic plasticity and neurogenesis through activation of intracellular pathways such as BDNF and PI3K–mTOR signaling. Pharmacological intervention involved BCI‐838 (5 mg/kg, oral administration), a prodrug metabolized into BCI‐632, which acts as a NAM of mGluR2/3. This compound selectively targets the mGluR2 and mGluR3 subtypes, which are predominantly presynaptic receptors regulating glutamate release and synaptic tone. Functionally, chronic BCI‐838 treatment significantly enhanced AHN, as evidenced by increased numbers of DCX‐positive immature neurons and BrdU‐labeled proliferating cells in the dentate gyrus compared to APP/PS1 controls. Behaviorally, BCI‐838 improved recognition memory in the novel object recognition task, restoring performance deficits observed in APP/PS1 mice, while transcriptomic analysis revealed upregulation of BDNF and PI3K–mTOR pathway components (e.g., PIK3C2A) and enrichment of glutamate receptor‐related pathways, supporting a molecular link between mGluR2/3 inhibition and neuroplasticity. Notably, these effects were comparable to those induced by physical exercise, and no additive benefit was observed with combined treatment, suggesting overlapping mechanisms. From a disease relevance perspective, the findings indicate that negative allosteric modulation of mGluR2/3 promotes neurogenesis and cognitive improvement in an AD model, potentially by engaging neurotrophic and translational signaling pathways; however, the study appropriately limits conclusions to preclinical outcomes without direct evidence of disease modification or long‐term neuroprotection [124].

Taken together, the studies reviewed in this section indicate that subtype‐specific pharmacological modulation of Group II mGluRs exerts diverse yet converging effects on synaptic plasticity, network excitability, and disease‐relevant behaviors, with both agonists and antagonists capable of normalizing aberrant glutamatergic signaling in a context‐dependent manner. Antagonists and NAMs such as LY341495, Ro‐4491533, and BCI‐838 can relieve excessive mGluR2/3–mediated inhibition to restore synaptic depression in FXS models or enhance neurogenesis and cognition in APP/PS1 mice, whereas orthosteric agonists like LY354740 and LY379268 engage presynaptic and postsynaptic mGluR2/3 to prime NMDAR–dependent LTP and suppress psychostimulant reinforcement. These findings underscore a bidirectional therapeutic potential in which the same receptor subtypes can be targeted in opposite directions across disorders characterized by either hypo‐ or hyperglutamatergic states, but they also highlight important limitations, including the heavy reliance on rodent models, predominant focus on acute or subchronic treatment paradigms, and lack of direct assessment of higher‐order cognitive or long‐term disease‐modifying outcomes in most experimental designs. Clinical translation has been further complicated by the variable success of mGluR2/3 agonists and modulators in psychiatric and neurodegenerative indications, where promising early‐phase results have not always been replicated in larger trials, and by the current scarcity of highly selective ligands that can clearly disentangle neuronal mGluR2 from astrocytic and neuronal mGluR3 contributions in vivo. Future work will, therefore, require systematic integration of subtype‐selective orthosteric and allosteric tools with cell type–specific targeting strategies, longitudinal behavioral and cognitive assessments, and cross‐species validation of mGluR2/3–dependent plasticity signatures, with particular emphasis on defining when agonism versus antagonism is appropriate for a given disease stage and circuit, and on establishing robust biomarkers that can report target engagement and synaptic effects in humans.

### 11.3. Group III mGluRs

Subtype‐specific pharmacological modulation of Group III mGluRs has emerged as a promising strategy to influence both immune and stem cell–related mechanisms that are relevant to neurodegeneration, by taking advantage of the Gi/o‐coupled nature of these receptors to dampen cAMP–dependent pro‐inflammatory or proapoptotic pathways and to bias cell fate decisions without directly interfering with fast ionotropic transmission. Across preclinical models, activation of mGluR4 and mGluR7 with selective orthosteric agonists and PAMs consistently reduces intracellular cAMP signaling, modulates MAPK and ASK1–p38 cascades, and reshapes cytokine profiles or apoptotic protein balance, thereby attenuating experimental autoimmune inflammation, oxidative stress‐induced neural stem cell death, and hypoxic–ischemic injury, while also regulating neural stem/progenitor proliferation and differentiation. These studies collectively position Group III mGluR subtypes as pharmacological entry points to indirectly support synaptic and network integrity in disorders where neuroinflammation, oxidative stress, and impaired neurogenesis contribute to disease progression, while appropriately restricting conclusions to in vitro and in vivo preclinical models rather than clinical efficacy.

Fallarino et al. [125] investigated the role of mGluR4 in regulating neuroinflammation through immune–glutamate interactions in experimental autoimmune encephalomyelitis (EAE), a model of multiple sclerosis. Mechanistically, the study demonstrates that mGluR4, a Gi/o‐coupled Group III receptor, reduces intracellular cAMP levels in dendritic cells (DCs), thereby modulating cytokine production and T helper (TH) cell differentiation; specifically, mGluR4 activation suppresses cAMP–dependent signaling pathways that otherwise promote pro‐inflammatory cytokines such as IL‐6 and IL‐23 while limiting anti‐inflammatory mediators like IL‐27. Pharmacological modulation was achieved using PHCCC, a selective positive allosteric modulator of mGluR4, and L‐AP4, an orthosteric agonist of Group III mGluRs, allowing both receptor‐specific and group‐level activation. These agents target the mGluR4 subtype, which was shown to be constitutively expressed in peripheral DCs and CD4^+^ T cells, with particularly high expression in CD11c^+^ DCs and regulatory T cell populations. Functionally, genetic deletion of mGluR4 resulted in exacerbated EAE severity, earlier disease onset, and increased mortality, accompanied by a shift toward a pro‐inflammatory TH17‐dominated immune response (increased IL‐17A, reduced Foxp3^+^ Treg cells). In contrast, pharmacological enhancement of mGluR4 signaling with PHCCC reduced intracellular cAMP in DCs, rebalanced cytokine production (increased IL‐12, IL‐27, TGF‐β; decreased IL‐6 and IL‐23), promoted Treg differentiation, and significantly attenuated disease severity and relapse frequency in vivo. Importantly, the histological and clinical score data, PHCCC–treated mice exhibited reduced demyelination and inflammatory infiltration compared to controls. From a disease relevance perspective, these findings demonstrate that activation of mGluR4 exerts a protective immunomodulatory role by shifting the balance from TH17–driven inflammation toward Treg–mediated regulation, highlighting this receptor as a potential therapeutic target in multiple sclerosis, while appropriately confining conclusions to preclinical EAE models and noting that therapeutic effects depend on early intervention and immune context [125].

Extending Group III mGluR pharmacology to neural stem/progenitor cells, Vernon et al. [126] investigated the role of these receptors in regulating proliferation and differentiation of human neural stem/progenitor cells (hNSPCs) derived from the ventral mesencephalon, thus, linking subtype‐specific modulation to regenerative mechanisms potentially relevant for disorders such as PD. Mechanistically, the study demonstrates that Group III mGluRs are Gi/o‐coupled receptors that inhibit adenylyl cyclase and reduce intracellular cAMP levels, as shown by the ability of the agonist L‐AP4 to suppress forskolin‐induced cAMP elevation in these cells. Pharmacological manipulation involved the broad‐spectrum Group III agonist L‐AP4, the mGluR7–selective allosteric agonist AMN082, as well as subtype‐selective ligands for mGluR4 (VU0155041) and mGluR8 ((S)‐3,4‐DCPG), enabling precise identification of subtype‐specific effects. These compounds target Group III receptors (mGluR4, mGluR7, and mGluR8), with expression confirmed at both mRNA and protein levels in undifferentiated hNSPCs, particularly for mGluR4, mGluR7, and mGluR8. Functionally, activation of Group III mGluRs with L‐AP4 produced a dose‐dependent reduction in cellular proliferation, evidenced by decreased MTT metabolic activity and reduced BrdU incorporation without affecting cell viability, indicating a specific antiproliferative effect rather than cytotoxicity. Importantly, subtype‐specific analysis revealed that this effect was primarily mediated by mGluR7, as AMN082 replicated the antiproliferative effect, whereas mGluR4 and mGluR8 agonists had minimal or no effect. During differentiation, activation of mGluR7 shifted cell fate toward astrocytic lineage, as shown by increased GFAP and S100β expression and a higher proportion of astrocyte‐like cells, alongside reduced neuronal (β‐III tubulin and TH) and oligodendrocyte (GalC) markers. From a disease relevance perspective, these findings indicate that mGluR7 activation regulates neural stem cell fate by suppressing proliferation and promoting astrocytic differentiation, suggesting potential implications for regenerative strategies in NDDs such as PD, where controlled modulation of stem cell proliferation and differentiation is critical, while the study appropriately limits conclusions to in vitro cellular models without direct in vivo validation of therapeutic efficacy [126].

Focusing again on mGluR4 but now under oxidative stress conditions relevant to neurodegenerative pathology, Zhang et al. [127] investigated the neuroprotective role of this receptor in oxidative stress‐induced apoptosis of NSCs, with particular emphasis on intracellular survival signaling pathways. Mechanistically, the study demonstrates that mGluR4 activation, a Gi/o‐coupled Group III receptor, suppresses intracellular stress signaling cascades, particularly the JNK and p38 MAPK pathways, which are key mediators of apoptosis under oxidative conditions; this is accompanied by modulation of apoptotic regulators, including restoration of the Bcl‐2/Bax balance and inhibition of procaspase‐8/9/3 cleavage. Pharmacological intervention was performed using the selective mGluR4 agonist VU0155041, with additional validation through the Group III antagonist MSOP and mGluR4–specific siRNA knockdown to confirm receptor specificity. These agents target the mGluR4 subtype, which is predominantly localized presynaptically but is also expressed in neural stem/progenitor cells, as confirmed by immunocytochemistry showing high coexpression with nestin‐positive NSCs (~91.6%). Functionally, exposure to H_2_O_2_ (100 µM, 12 h) significantly reduced NSC viability (~62% loss), whereas pretreatment with VU0155041 restored viability in a concentration‐dependent manner and significantly reduced apoptosis, as demonstrated by decreased PI‐ and TUNEL‐positive cells (~30% → ~13%–15%). At the molecular level, VU0155041 inhibited H_2_O_2_–induced phosphorylation of JNK and p38, and these effects were mimicked by specific MAPK inhibitors (SP600125 and SB203580), indicating that mGluR4–mediated protection operates through suppression of stress‐activated kinase signaling. Importantly, both pharmacological blockade (MSOP) and genetic knockdown of mGluR4 abolished these protective effects, confirming subtype specificity. From a disease relevance perspective, these findings indicate that activation of mGluR4 confers resistance to oxidative stress‐induced NSC apoptosis by modulating MAPK–dependent apoptotic pathways, suggesting potential utility in enhancing stem cell survival in neurodegenerative conditions such as PD and stroke, where oxidative stress is a major pathological driver, while the study appropriately limits conclusions to in vitro NSC models without direct in vivo validation of therapeutic efficacy [127].

Building on this oxidative stress‐related work and further refining the link between subtype‐specific modulation and hypoxic–ischemic injury, Zhang et al. [128] investigated the neuroprotective role of mGluR4 in human NSCs (hNSCs) under hypoxic–ischemic conditions using an oxygen–glucose deprivation (OGD) model. Mechanistically, the study demonstrates that mGluR4 activation, a Gi/o‐coupled Group III receptor, inhibits adenylyl cyclase activity, leading to reduced cAMP levels and suppression of PKA signaling, which in turn attenuates activation of the ASK1–p38 MAPK apoptotic pathway. Pharmacological modulation was achieved using the selective mGluR4 agonist VU0155041 (1–50 μM), with pathway validation through ASK1 inhibitor (NQDI‐1), p38 MAPK inhibitor (SB239063), and p38 activator (asiatic acid). These interventions target the mGluR4 subtype, which is expressed in hNSCs and localized functionally to regulate cellular stress responses under pathological conditions. Functionally, OGD significantly induced apoptosis in hNSCs, as shown by increased TUNEL‐positive cells, reduced cell viability, elevated cleaved caspase‐8/9/3 levels, and disruption of the Bcl‐2/Bax balance, whereas pretreatment with VU0155041 restored cell viability, reduced apoptosis rates, and normalized apoptotic signaling markers in a concentration‐dependent manner. At the signaling level, as illustrated in the western blot data, VU0155041 suppressed OGD–induced phosphorylation of ASK1 and p38 MAPK, effects that were mimicked by pharmacological inhibitors and abolished by mGluR4 knockdown, confirming receptor‐specific mediation. Importantly, activation of p38 with asiatic acid reversed the protective effects of mGluR4 activation, further validating the involvement of the ASK1–p38 axis. From a disease relevance perspective, these findings indicate that activation of mGluR4 enhances resistance of NSCs to hypoxic–ischemic injury by suppressing stress‐induced apoptotic signaling, suggesting potential utility in conditions such as hypoxic–ischemic brain injury (HIBI) and stroke, where stem cell survival is a critical limitation for regenerative therapies, while the study appropriately restricts conclusions to in vitro hNSC models with no direct in vivo validation of therapeutic efficacy [128].

Collectively, the available evidence indicates that subtype‐specific pharmacological modulation of Group III mGluRs, particularly mGluR4 and mGluR7, provides a coherent mechanistic framework for influencing neuroinflammatory and stem cell–related processes that are highly relevant to neurodegenerative and hypoxic–ischemic conditions, but current data remain predominantly preclinical and mechanistic in nature. The studies summarized in this section converge on the capacity of Gi/o‐coupled Group III mGluRs to suppress cAMP–dependent signaling, reshape cytokine milieus, and inhibit stress‐activated kinase pathways (JNK, p38, and ASK1–p38), thereby attenuating immune‐driven demyelination in EAE, reducing oxidative and OGD–induced apoptosis in NSCs, and modulating proliferation and fate specification of hNSPCs, with downstream implications for disorders such as multiple sclerosis [129], PD, stroke, and HIBI. However, several important limitations temper the translational interpretation of these findings: most experiments are conducted in vitro or in acute rodent models with relatively short observation windows; pharmacological tools such as PHCCC, VU0155041, AMN082, and L‐AP4, while subtype‐preferential, do not always provide absolute selectivity across the Group III family; potential issues such as receptor desensitization, biased signaling, off‐target effects, and long‐term safety profiles are not comprehensively addressed in these mechanistic studies. In addition, clinical experience with Group III mGluR ligands remains very limited, with early trials and translational efforts in PD and related indications yielding mixed or inconclusive results, highlighting challenges in optimizing dosing regimens, therapeutic time windows, and patient stratification, as well as in aligning rodent model endpoints with clinically meaningful outcomes. Future work should, therefore, prioritize the development and rigorous pharmacological characterization of highly selective, brain‐penetrant allosteric modulators and orthosteric ligands for individual Group III subtypes, especially mGluR4 and mGluR7, combined with in vivo validation in chronic and comorbid models that better recapitulate the complex pathophysiology of human NDDs [5–7, 129–132], neuroinflammatory disorders, cardiovascular disorders, and metabolic disorder linked advanced glycations end products [133–135], including progressive protein aggregation, glial reactivity, and impaired synaptic plasticity. Integrating these ligands into studies that directly interrogate mGluR–dependent synaptic plasticity, neuron–glia interactions, and region‐specific network dynamics in disease‐relevant circuits, alongside careful assessment of safety, tolerance, and potential interactions with existing standard‐of‐care therapies, will be essential to determine whether the robust cellular and molecular effects observed in preclinical models can be translated into clinically meaningful neuroprotective or disease‐modifying interventions.

## 12. Challenges and Future Research Directions

Despite substantial advances in understanding mGluR–dependent synaptic plasticity, several conceptual, methodological, and translational challenges remain that limit the integration of molecular, synaptic, and circuit‐level findings into a unified framework. A central challenge arises from the spatiotemporal complexity of mGluR organization, as highlighted by their perisynaptic and extrasynaptic localization across neuronal and glial compartments. While ultrastructural and super‐resolution studies have established subtype‐specific nanoscale distributions [18, 21, 22], these findings are largely derived from static or ex vivo preparations. Consequently, how dynamic receptor trafficking, activity‐dependent redistribution, and disease‐associated remodeling alter mGluR positioning in vivo remains poorly understood. Furthermore, subtype‐specific localization patterns across circuits, developmental stages, and pathological conditions are incompletely mapped, limiting our ability to link receptor distribution with functional plasticity outcomes.

At the level of intracellular signaling, a major limitation lies in the incomplete characterization of context‐dependent pathway engagement. mGluRs can activate diverse signaling cascades, including PLC/IP_3_/DAG, cAMP inhibition, ERK/MAPK pathways, and ion channel modulation, yet the rules governing how these pathways are selectively recruited remain unclear [23, 29]. Experimental evidence demonstrates that even within a single receptor subtype, signaling outcomes can vary depending on phosphorylation states, scaffold interactions, and cellular context [24, 25]. Additionally, splice variant‐specific signaling and cell type–specific coupling, particularly in neurons versus astrocytes, are insufficiently resolved. Most current insights are derived from reductionist systems such as heterologous expression models or acute slices, which do not fully capture the dynamic interplay between signaling pathways during behaviorally relevant plasticity. Addressing these limitations will require integrated approaches combining in vivo imaging, phosphoproteomics, and circuit‐specific manipulations to map signaling networks with high spatiotemporal resolution.

Another major challenge concerns the integration of neuron–glia interactions into models of synaptic plasticity. Astrocytic mGluRs, particularly mGluR1, mGluR3, and mGluR5, have emerged as critical regulators of gliotransmission, synaptogenesis, and plasticity thresholds [32, 33, 42]. However, the relative contribution of astrocytic versus neuronal mGluRs to LTP and LTD remains difficult to disentangle, particularly in vivo. Moreover, astrocytic signaling exhibits strong developmental and pathological reactivation patterns, such as the reexpression of mGluR5 in reactive astrocytes during epilepsy, neuropathic pain, and neurodegeneration [37, 43]. While these findings suggest a role in maladaptive plasticity, the precise mechanisms by which astrocytic mGluRs drive circuit‐level dysfunction remain unclear. Future studies must incorporate cell type–specific targeting strategies and real‐time imaging of astrocytic Ca^2+^ dynamics to determine how neuron–glia signaling contributes to adaptive versus pathological plasticity. The relationship between classical ionotropic plasticity and mGluR–dependent mechanisms also presents an unresolved challenge. Although ionotropic receptors establish the initial conditions for synaptic plasticity through Ca^2+^ influx and membrane depolarization [46, 47], mGluRs modulate these processes by regulating intracellular signaling, receptor trafficking, and plasticity thresholds. However, current models often treat these systems in isolation, and quantitative frameworks linking ionotropic and metabotropic signaling across different activity regimes are lacking. Variability in receptor subunit composition, extracellular ionic conditions, and scaffold availability further complicates this relationship [45, 48]. Bridging this gap will require multiscale models that integrate fast synaptic transmission with slower modulatory signaling, particularly in intact neural circuits during learning and behavior.

A critical translational challenge is the limited availability of reliable biomarkers and in vivo measures of mGluR–dependent plasticity in humans. While preclinical studies have identified receptor‐specific roles in neurodegenerative and neuropsychiatric disorders, including AD, PD, and schizophrenia [12–16], translation to clinical settings remains constrained by methodological heterogeneity and species differences [9, 10]. Current imaging approaches, such as PET–based receptor mapping, provide limited functional insight into plasticity dynamics. Moreover, disease stage–specific alterations in mGluR signaling are insufficiently characterized, making it difficult to determine when and how therapeutic interventions should be applied. Longitudinal studies integrating electrophysiology, imaging, and molecular profiling in patients will be essential to bridge this gap. From a therapeutic perspective, targeting mGluRs presents both opportunities and challenges. Although allosteric modulators and subtype‐selective ligands offer improved specificity compared to orthosteric drugs, achieving pathway‐selective modulation without disrupting physiological plasticity remains difficult. The same receptor can promote LTP, LTD, or metaplasticity depending on network state and prior activity [26, 49], raising concerns about unintended effects of global receptor modulation. Future strategies should focus on biased signaling approaches, circuit‐specific interventions, and combination therapies that account for network‐level dynamics rather than single‐receptor targeting.

Several key directions are likely to drive progress in this field. First, advances in in vivo imaging and single‐cell resolution techniques will enable direct observation of mGluR signaling and plasticity in intact circuits. Second, cell type–specific genetic and chemogenetic tools will allow precise dissection of neuronal versus astrocytic contributions. Third, integration of computational modeling with experimental data will help resolve the multiscale complexity of mGluR–dependent plasticity. Finally, the development of translational biomarkers and personalized therapeutic strategies will be essential for leveraging mGluR signaling in clinical contexts. While significant progress has been made in delineating the molecular and cellular mechanisms of mGluR–dependent plasticity, major gaps remain in linking these processes across spatial scales, cell types, and disease states. Addressing these challenges will require interdisciplinary approaches that integrate structural biology, systems neuroscience, and clinical research, ultimately enabling the development of precision therapies aimed at restoring adaptive plasticity while preventing maladaptive circuit remodeling.

## 13. Conclusion

mGluR–dependent synaptic plasticity emerges from this review as a fundamentally integrative and context‐sensitive regulatory system that extends beyond classical models of synaptic transmission. By linking extracellular glutamatergic signaling to intracellular biochemical networks, mGluRs orchestrate a wide spectrum of processes spanning receptor trafficking, calcium dynamics, protein synthesis, and neuron–glia interactions. Their strategic perisynaptic and extrasynaptic localization enables them to function as modulators of synaptic state rather than direct mediators of fast transmission, allowing them to shape both the threshold and persistence of LTP and LTD. Importantly, mGluR signaling operates across multiple spatial and temporal scales, from molecular and synaptic mechanisms to circuit‐level remodeling, positioning these receptors as key determinants of how neural systems adapt to physiological demands and environmental challenges. At the same time, the evidence synthesized in this review highlights that dysregulation of mGluR–dependent plasticity represents a convergent mechanism underlying maladaptive circuit remodeling across diverse brain disorders. The dual capacity of mGluRs to support both adaptive and pathological plasticity underscores the need for a nuanced, systems‐level understanding of their function. Future progress will depend on bridging gaps between molecular signaling, cell type–specific contributions, and in vivo circuit dynamics, while advancing translational strategies that selectively target beneficial signaling pathways without disrupting physiological network function. By framing mGluR–dependent plasticity as a dynamic regulator of neural circuit stability and flexibility, this review provides a conceptual foundation for the development of precision therapeutic approaches aimed at restoring functional plasticity in neurological and neuropsychiatric disease.

**Nomenclature**
AIDA: (RS)‐1‐aminoindan‐1,5‐dicarboxylic acid
Akt: Protein kinase B
ALS: Amyotrophic lateral sclerosis
AMN082: mGluR7 agonist
AMPAR: Alpha‐amino‐3‐hydroxy‐5‐methyl‐4‐isoxazolepropionic acid receptor
AP5/APV: D‐(−)‐2‐amino‐5‐phosphonovaleric acid
APP: Amyloid beta precursor protein
Arc: Activity‐regulated cytoskeletal‐associated protein
ASK1: Apoptosis signal‐regulating kinase 1
ApoE: Apolipoprotein E
BACHD: Bacterial artificial chromosome‐mediated transgenic mouse model
BAPTA: 1,2‐Bis(o‐aminophenoxy)ethane‐N,N,N′,N′‐tetraacetic acid
BCI‐838: mGluR2/3 antagonist (prodrug; antidepressant‐like effects)
BDNF: Brain‐derived neurotrophic factor
BLI: Biolayer interferometry
CA1: Cornu ammonis 1
CA3: Cornu ammonis 3
CaMKII: Calcium/calmodulin‐dependent protein kinase II
CB1Rs: Cannabinoid receptor type 1
CD4^+^ T: Cluster of differentiation 4 positive T lymphocytes
CDK2: Cyclin‐dependent kinase 2
CDPPB: 3‐Cyano‐N‐(1,3‐diphenyl‐1‐H‐pyrazol‐5‐yl)benzamide
CHPG: Chlorohydroxyphenylglycine
cAMP: Cyclic adenosine monophosphate
CNQX: 6‐Cyano‐7‐nitroquinoxaline‐2,3‐dione
CP‐AMPARs: Ca^2+^‐permeable AMPA receptors
CREB: Cyclic AMP response element‐binding protein
CXCL1: C‐X‐C motif chemokine ligand 1
CXCL2: C‐X‐C motif chemokine ligand 2
DBS: Deep brain stimulation
DCG‐IV: (2S,2R,3R)‐2‐(2,3‐dicarboxycyclopropyl)glycine
DCPG: (S)‐3,4‐dicarboxyphenylglycine
DFB: 3,3^′^‐Difluorobenzaldazine
DHPG: (S)‐3,5‐dihydroxyphenylglycine
DLPFC: Dorsolateral prefrontal cortex
DSI: Depolarization‐induced suppression of inhibition
DSE: Depolarization‐induced suppression of excitation
EAAC1: Excitatory amino acid carrier 1
EAAT2: Excitatory amino acid transporter 2
EAATs: Excitatory amino acid transporters
EGTA: Ethylene glycol‐bis(β‐aminoethyl ether)‐N,N,N′,N′‐tetraacetic acid
eLTP: Excitatory long‐term potentiation
ERK: Extracellular signal‐regulated kinase
EPSC: Excitatory postsynaptic current
EPSP: Excitatory postsynaptic potential
FA: Fractional anisotropy
FGF22: Fibroblast growth factor 22
FKBP5: FK506 binding protein 5
fEPSP: Field excitatory postsynaptic potential
FMR1: Fragile X mental retardation 1
FMRP: Fragile X mental retardation protein
FRET: Förster resonance energy transfer
Fyn: Src family tyrosine kinase
GABAAR: Gamma‐aminobutyric acid Type A receptor
GABAB: Gamma‐aminobutyric acid Type B receptor
GFAP: Glial fibrillary acidic protein
GLAST: Glutamate aspartate transporter
GLT‐1: Glutamate transporter 1
GPCR: G protein‐coupled receptor
GRM2: Glutamate metabotropic receptor 2
GRM3: Glutamate metabotropic receptor 3
HAMD: Hamilton depression rating scale
IGF‐1: Insulin‐like growth factor 1
IL‐1β: Interleukin‐1 beta
IL‐6: Interleukin‐6
IL‐23: Interleukin‐23
IL‐27: Interleukin‐27
ILD: Interaural level difference
IP_3_: Inositol 1,4,5‐trisphosphate
ITD: Interaural time difference
JNK: c‐Jun N‐terminal kinase
LFS: Low‐frequency stimulation
L‐AP4: L‐(+)‐2‐amino‐4‐phosphonobutyric acid
LTD: Long‐term depression
LTP: Long‐term potentiation
MAPK: Mitogen‐activated protein kinase
MCPG: α‐Methyl‐4‐carboxyphenylglycine
MD: Mean diffusivity
MEK: Mitogen‐activated protein kinase kinase
mEPSC: Miniature excitatory postsynaptic current
MPEP: 2‐Methyl‐6‐(phenylethynyl)pyridine
MPTP: 1‐Methyl‐4‐phenyl‐1,2,3,6‐tetrahydropyridine
MRS: Magnetic resonance spectroscopy
MTT: 3‐(4,5‐Dimethylthiazol‐2‐yl)‐2,5‐diphenyltetrazolium bromide
mTOR: Mammalian target of rapamycinm
RNA: Messenger RNA
NAMs: Negative allosteric modulators
NDDs: Neurodegenerative disorders
NGF: Nerve growth factor
NGFI‐A: Nerve growth factor‐inducible protein A
NMDA: N‐methyl‐D‐aspartate
NMDARs: N‐methyl‐D‐aspartate receptors
NRSF: Neuron‐restrictive silencer factor
NT‐3: Neurotrophin‐3
NPC1: Niemann–Pick disease Type C1 protein
P2X: P2X purinergic receptors
PAMs: Positive allosteric modulators
PANSS: Positive and negative syndrome scale
PD: Parkinson’s disease
PDK1: 3‐Phosphoinositide‐dependent protein kinase‐1
PET: Positron emission tomography
PHCCC: Positive allosteric modulator of mGluR4
PI3K: Phosphoinositide 3‐kinase
PKC: Protein kinase C
PKMζ: Protein kinase M zeta
PLC: Phospholipase C
preclinical MIA: Preclinical maternal immune activation
PSD‐95: Postsynaptic density protein 95
PSQI: Pittsburgh sleep quality index
PTP: Protein tyrosine phosphatases
REM sleep: Rapid eye movement sleep
REST: RE1‐silencing transcription factor
rTMS: Repetitive transcranial magnetic stimulation
SANS: Scale for the assessment of negative symptoms
SCN: Suprachiasmatic nucleus
SCS: Spinal cord stimulation
SF‐1: Steroidogenic factor 1
SHANK1: SH3 and multiple ankyrin repeat domains protein 1
SHANK3: SH3 and multiple ankyrin repeat domains protein 3
SMA: Spinal muscular atrophy
SNARE: Soluble N‐ethylmaleimide‐sensitive factor attachment protein receptor
Src: Src family tyrosine kinase
StAR: Steroidogenic acute regulatory protein
STEP: Striatal‐enriched protein phosphatase
TBOA: DL‐threo‐β‐benzyloxyaspartate
tDCS: Transcranial direct current stimulation
TH17: T helper 17 cells
TMS‐EEG: Transcranial magnetic stimulation–electroencephalography
TSC2: Tuberous sclerosis complex 2
TSP1: Thrombospondin‐1
TUNEL‐positive cells: Terminal deoxynucleotidyl transferase dUTP nick end labeling positive cells
TUS: Transcranial ultrasound stimulation
U‐73122: Phospholipase C inhibitor
VU0155041: Positive allosteric modulator of mGluR4
VEGF: Vascular endothelial growth factor
VNS: Vagus nerve stimulation
VDCCs: Voltage‐dependent calcium channels.

## Author Contributions

**Shaik Basha and Aradhika Vijeev**: conception, design, analysis, drafting of the original paper. **Spandana S. Nadig**, **Kripa Agarwal**, and **Vibhuti Meharchandani:** review drafting and editing. **Aparna Ramakrishna Pai:** original draft writing and editing. **Krishna Kishore Mahato:** guidance, conception, design, the drafting of the paper, the final approval of the version to be published.

## Funding

This study was supported by the Manipal Academy of Higher Education (MAHE), Manipal, Karnataka, India, the Indian Council of Medical Research (ICMR) (Grants 17x(3)/Adhoc/33/2022‐ITR and EM/Dev/SG/75/0782/2023), the Department of Biotechnology – Boost to University Interdisciplinary Life science Departments for Education and Research (DBT‐BUILDER) (Grant BT/INF/22/SP43065/2021), and the Department of Science and Technology – “Innovation in Science Pursuit for Inspired Research (DST‐INSPIRE)” (Grant IF:220005), Govt. of India.

## Disclosure

During the preparation of this work, the scientific writer used Grammarly and OpenAI (Chat‐GPT) for spell check and to improve language and readability. After using Grammarly and OpenAI, the scientific writer and authors reviewed and edited the content. All authors agree to be accountable for all aspects of the work and approved the final version of the manuscript.

## Conflicts of Interest

The authors declare no conflicts of interest.

## Data Availability

Data sharing is not applicable as no new data generated or the article describes entirely theoretical research. The data presented in this study are available in the referenced articles.
